# Missing data in ecology: Syntheses, clarifications, and considerations

**DOI:** 10.1002/ecm.70037

**Published:** 2025-11-05

**Authors:** Michael Dumelle, Rob Trangucci, Amanda M. Nahlik, Anthony R. Olsen, Kathryn M. Irvine, Karen Blocksom, Jay M. Ver Hoef, Claudio Fuentes

**Affiliations:** 1 United States Environmental Protection Agency, Office of Research and Development, Corvallis, Oregon, USA; 2 Department of Statistics, Oregon State University, Corvallis, Oregon, USA; 3 U.S. Geological Survey Northern Rocky Mountain Science Center, Bozeman, Montana, USA; 4 Marine Mammal Laboratory, Alaska Fisheries Science Center, NOAA Fisheries, Seattle, Washington, USA

**Keywords:** Bayesian modeling, complete case analysis, contingency variable, data augmentation, imputation, missing at random, missing completely at random, missing not at random, prediction, spatial regression model

## Abstract

In ecology and related sciences, missing data are common and occur in a variety of different contexts. When missing data are not handled properly, subsequent statistical estimates tend to be biased, inefficient, and lack proper confidence interval coverage. Missing data are often grouped into three categories: missing completely at random (MCAR), missing at random (MAR), and missing not at random (MNAR). We review each category and compare their benefits and drawbacks. We review several approaches to handling missing data including complete case analysis, imputation, inverse probability weighting, and data augmentation. We clarify what types of variables should accompany imputation methods and how those variables are influenced by the analysis methods. Additionally, we discuss missing data that lack a formal basis for measurement and hence are fundamentally different from MCAR, MAR, and MNAR missing data. Throughout, we introduce concepts and numeric examples using both simulated data and data from the United States Environmental Protection Agency’s 2016 National Wetland Condition Assessment. We conclude by providing five considerations for ecologists and other scientists handling missing data.

## INTRODUCTION

A data analysis aims to answer important scientific questions by studying relationships among variables, estimating population parameters, or making predictions. Ecological data often have some amount of missingness that makes these important scientific questions harder to answer. Moreover, mishandling missing data can yield statistical estimates that are biased, inefficient, and lack proper confidence interval (CI) coverage (Little & Rubin, 2019). Throughout this work, we synthesize methods for handling missing data, clarify some common misunderstandings, and give some considerations. Our intent is to provide ecologists and scientists in other disciplines (e.g., environmental science, geology, geochemistry, and soil science) with tools to more effectively handle missing data.

Missing data approaches have matured tremendously over the past few decades (Rubin, 1996). Specific missing data approaches (e.g., imputation) have been popularized in disciplines such as medicine (Austin et al., 2021; Bennett, 2001; Carpenter & Smuk, 2021; Little et al., 2012; Wood et al., 2004), psychology (Graham, 2009; Roth, 1994; Schlomer et al., 2010; Schoemann et al., 2024), social science (Little & Rubin, 1989; Newman, 2014; Saunders et al., 2006), epidemiology (Donders et al., 2006; Pedersen et al., 2017; Perkins et al., 2018), and survey research (Andridge & Little, 2010; Brick & Kalton, 1996; Laaksonen, 2018; Lumley, 2011; Carpenter et al., 2023). In ecology specifically, missing data approaches have been used to study biodiversity (Bowler et al., 2024; Kim et al., 2018; Taugourdeau et al., 2014), species trait indices (Johnson et al., 2021; Penone et al., 2014), species distribution models (Łopucki et al., 2022; Ter Braak et al., 1994), meta-analyses (Ellington et al., 2015), behavioral ecology (Nakagawa & Freckleton, 2011), plant ecology (Dray & Josse, 2015), water quality (Srebotnjak et al., 2012), forestry (Van Deusen, 1997), and animal movement (Scharf et al., 2017). However, modern missing data approaches still lack sufficient adoption in the ecological and related sciences (Nakagawa, 2015; Nakagawa & Freckleton, 2008).

We introduce missing data concepts and methods using data from the United States Environmental Protection Agency’s (USEPA) 2016 National Wetland Condition Assessment (2016 NWCA, Kentula & Paulsen, 2019). We begin by defining three types of missing data: missing completely at random (MCAR), missing at random (MAR), and missing not at random (MNAR). We then review several missing data approaches, including complete case analysis (CCA), imputation, inverse probability weighting, and data augmentation. We discuss relevant missing data diagnostics and clarify several nuances regarding which variables to use to inform imputations. We compare and contrast missing data approaches using a variety of numerical studies applied to both simulated and 2016 NWCA data. Finally, we provide five explicit missing data considerations that ecologists and other scientists can leverage to more effectively handle missing data and improve scientific understanding.

## THE NWCA DATA

We use a running example from the 2016 NWCA to elucidate different missing data approaches. More broadly, the NWCA measures US wetland condition on repeating, 5-year cycles (2011, 2016, 2021, and planned for 2026). For each cycle, approximately 1000 sample locations (i.e., 10,000 m^2^ wetland sites) are selected throughout the conterminous United States using a spatially balanced probability (i.e., random) design (Dumelle, Kincaid, et al., 2023; Olsen et al., 2019; Stevens & Olsen, 2004). Each site is sampled during a 1-day field visit during an Index Period (typically, April to October) for dozens of variables, each characterizing a distinct aspect of wetland condition. Chemical, physical, and biological samples are collected and analyzed using consistent field-based and laboratory-based protocols (Herlihy et al., 2019; McCauley et al., 2019; USEPA, 2015, 2016). Throughout, we introduce missing data concepts using several variables from the 2016 NWCA: vegetation type, the dominant vegetation type (herbaceous or woody); soil hardening, a measure of anthropogenic activities that affect soil permeability; and vegetation multimetric index (VMMI), a measure of overall vegetation quality. For each variable, Table 1 provides a brief summary, while Magee et al. (2019) and USEPA (2023) provide technical details. In total, 967 wetlands were sampled for these variables as part of the 2016 NWCA.

Vegetation type of the sampled sites was determined from United States Fish and Wildlife Survey National Wetland Inventory spatial layers (Wilen & Bates, 1995) and confirmed in the field. Seven Cowardin vegetation types (Cowardin, 1979) were aggregated into wetland classes describing the dominant vegetation type at the site: woody or herbaceous.

To collect soil hardening data, field crews used a set list of eight human-mediated physical alterations associated with these variables within the 0.5-ha wetland site assessment area (AA) and the 100-m buffer around the AA (USEPA, 2016). Physical alterations that indicated soil hardening included (i.e., reduced soil permeability), for example, soil compaction from cattle, non-paved trails, vehicle ruts, paved roads, and impervious surfaces. Each observed physical alteration was scored based on the proximity to the AA, ranging from 25 points (inside the AA) to 1 point (at the furthest edge of the buffer). The eight physical alteration scores associated with each of the three variables were summed to calculate the soil hardening index. The maximum score for the soil hardening index was 424 points (USEPA, 2023).

To collect VMMI data, five 100-m^2^ plots within the sampled wetland sites were surveyed by a field botanist for the presence, cover, and height class of all vascular plant species, tree species, nonvascular groups, and for ground surface attributes (USEPA, 2016). The index period coincides with peak vegetation to maximize observations and identification of plant species on site. Observed plant nomenclatures were standardized by name–site pairs to the United States Department of Agriculture Natural Resources Conservation Service PLANTS database (USDA-NRCS, 2020). Metrics describing species traits (life history, wetland indicator status, native status, and coefficients of conservatism) were described for each taxon, taxon-state, or taxon-region (depending on the metric). Candidate metrics were screened for range, repeatability, responsiveness, and redundancy. The strongest performing metrics (e.g., relative cover of native monocots, percent richness of native species, and relative cover of native species) were used to develop and calculate four VMMIs: one for each combination of (1) site type (i.e., tidal or inland) and (2) dominant vegetation type (i.e., woody or herbaceous). Different metrics were used in each VMMI, with tidal VMMIs comprised of six metrics and inland VMMIs comprised of four metrics. Metric scores were combined for each VMMI, which was ultimately scored on a continuous scale from 0 to 100, with higher values reflecting healthier vegetation (i.e., less disturbed conditions); see USEPA (2023) for further details.

## AN OVERVIEW OF MISSING DATA THEORY

### The goals of a statistical analysis

Tredennick et al. (2021) make explicit three potential components of a statistical analysis, each with separate goals. The first statistical analysis component is data exploration, which leverages approaches like data visualizations and summary statistics to characterize the distributions of variables through means and variances, and correlations (i.e., covariances), among others. Data exploration is often used to generate new scientific hypotheses that are more carefully evaluated later. The second statistical analysis component is inference, which involves estimating population parameters (e.g., slope parameters in a linear regression model) and characterizing uncertainty in those estimates to make inferential conclusions based on p values (Wasserstein & Lazar, 2016) or Bayes factors (Kass & Raftery, 1995) from null hypothesis tests. These conclusions may be causal or noncausal, depending on context (Holland, 1986). Based on parameter estimates and their corresponding uncertainties, CIs and credible intervals can be constructed to quantify a range of plausible values for the underlying parameter. The third statistical analysis component is prediction, which aims to predict the value of an unobserved random variable that is separate from the data used to construct a model. For example, one may build a linear regression model from data on a set of sites and use the fitted model to predict a value at a new site and quantify uncertainty in that prediction to construct a prediction interval. Missing data, ubiquitous in practice, make the goals of each statistical analysis component harder to achieve. However, even in the presence of missing data, it is still possible to achieve these goals as long as adequate methods are used to handle the missing data.

### Definitions, patterns, and mechanisms

Causes of missing data are highly variable but can be generally classified into three broad groups: MCAR, MAR, and MNAR (Rubin, 1976). Before making these definitions explicit, we describe some general notation that will be used henceforth. Let Y be an n×p matrix whose rows index each of the n units (i.e., sites) and whose columns index each of the p variables potentially observable for each unit. Each unit–variable combination is called an item, and hence there are np items in Y (p items for each of n units). The matrix Y represents all outcomes that could possibly be observed for each item. Next, let R be an n×p matrix that identifies the missing data pattern, that is, which items in Y are observed or missing (Little & Rubin, 2019). That is, an item in R has a value of one if the corresponding item in Y is observed (i.e., not missing) and a value of zero if the corresponding item in Y is missing (i.e., not observed). Just as Y represents all possible outcomes a researcher might observe, R represents the collection of all possible missingness patterns. Put another way, the quantities Y and R are random variables and are not fixed (shortly we talk about specific realizations of Y and R that do take on fixed values). The probabilistic relationship between Y and R (i.e., the items and the missingness indicators) is called the missing data mechanism (Little & Rubin, 2019). Colloquially, we often refer to missing data by their mechanism name—for example, by MAR data, we mean missing data with a MAR mechanism. The missing data patterns and mechanisms guide how the missing data are ultimately handled.

We may partition Y into two distinct groups based on $R:Y_{\text{obs}}$ and $Y_{\text{mis}}$. The group $Y_{\text{obs}}$ contains the items in Y that are observed, while the group $Y_{\text{mis}}$ contains the items in Y that are missing (i.e., not observed). Providing these definitions is important because notation varies throughout the literature—for example, Rubin (2004) places variables whose items are fully observed into a separate matrix called X. We simply assume that Y contains all possible data.

The matrices $Y,R,Y_{\text{obs}}$, and $Y_{\text{mis}}$ are very general and represent all possible observed items and missingness patterns. In practice, however, we only see one dataset with a single set of observed items and a single missingness pattern. We indicate the values $Y,R,Y_{\text{obs}}$, and $Y_{\text{mis}}$ which are realized (i.e., are assigned a particular value) for a single dataset as $y,r,y_{\text{obs}}$, and $y_{\text{mis}}$, respectively (Table 2). Shortly we provide an example of these quantities using wetland data, and in Appendix S1: Section S1, we provide an additional example along with R code to further elucidate these relationships. While we directly observe the values in $y_{\text{obs}}$, the values in $y_{\text{mis}}$ are hidden from us. However, they generally still exist in practice and hence could have been directly observed under other missingness patterns (i.e., other random outcomes in R). Shortly we explore specific scenarios where $y_{\text{mis}}$ is not directly observable.

Consider the following matrix y, which, for six units, contains the observable values of three 2016 NWCA variables (USEPA, 2023): vegetation type (VT), soil hardening (SH) scores, and VMMI scores (Table 1).


$$y=\left[\begin{array}{cccc} \text{Unit} & \text{VT} & \text{SH} & \text{VMMI}\\ 1 & \text{Woody} & 97.5 & 44.0\\ 2 & \text{Herbaceous} & 29.5 & 31.2\\ 3 & \text{Herbaceous} & 90.4 & 39.4\\ 4 & \text{Woody} & 79.6 & 25.0\\ 5 & \text{Herbaceous} & 33.6 & 41.2\\ 6 & \text{Woody} & 65.9 & 38.7 \end{array}\right].$$

Suppose that VT and SH are observed for each unit but VMMI is missing in the second and sixth units. This missingness pattern is represented by r, which is given by

$$r=\left[\begin{array}{cccc} \text{Unit} & \text{VT} & \text{SH} & \text{VMMI}\\ 1 & 1 & 1 & 1\\ 2 & 1 & 1 & 0\\ 3 & 1 & 1 & 1\\ 4 & 1 & 1 & 1\\ 5 & 1 & 1 & 1\\ 6 & 1 & 1 & 0 \end{array}\right].$$

Combining y and r yields the matrices

(1) $$y_{\text{obs}}=\left[\begin{array}{cccc} \text{Unit} & \text{VT} & \text{SH} & \text{VMMI}\\ 1 & \text{Woody} & 97.5 & 44.0\\ 2 & \text{Herbaceous} & 29.5 & *\\ 3 & \text{Herbaceous} & 90.4 & 39.4\\ 4 & \text{Woody} & 79.6 & 25.0\\ 5 & \text{Herbaceous} & 33.6 & 41.2\\ 6 & \text{Woody} & 65.9 & * \end{array}\right]\;\text{and}\;y_{\text{mis}}=\left[\begin{array}{cccc} \text{Unit} & \text{VT} & \text{SH} & \text{VMMI}\\ 1 & * & * & *\\ 2 & * & * & 31.2\\ 3 & * & * & *\\ 4 & * & * & *\\ 5 & * & * & *\\ 6 & * & * & 38.7 \end{array}\right].$$

This example highlights that $y_{\text{mis}}$ represents a single outcome, namely the outcome that the second element of VMMI is 31.2 and the sixth element is 38.7. These outcomes are not observed by the analyst, however. Instead, to the analyst, the second and sixth elements of VMMI could be anywhere in the interval [0, 100]. We will return to these $y,r,y_{\text{obs}}$, and $y_{\text{mis}}$ values shortly, after describing the MCAR, MAR, and MNAR paradigms.

Data are MCAR if the probability that R=r does not depend on any y. Put another way, the probability of missingness for each realized item is unrelated to the values of each item. More formally, MCAR data satisfy the following condition (Little, 2021):

$$\text{Pr}(R=r|Y=\tilde{y})=\text{Pr}\left(R=r|Y=y^{⋆}\right)$$

for any two realizations $\tilde{y}$ and $y^{⋆}$. The term $\text{Pr}(R=r|Y=\tilde{y})$ is read as “the probability that R=r given Y is set to a particular value, namely $\tilde{y}$.” When data are MCAR, the underlying distributions of the observed and missing data are the same (Example 1.13, Little & Rubin, 2019). A more stringent condition than MCAR is that all possible outcomes of R (the missingness patterns) are independent of all possible outcomes of Y (the datasets). When this condition is satisfied, the data are missing-always-completely-at-random (MACAR); this nuance is subtle (see Heitjan & Basu, 1996; Mealli & Rubin, 2015; Seaman et al., 2013, for more details).

A more general data assumption than MCAR is MAR. Data are MAR if the probability that R=r depends on y only through $y_{\text{obs}}$, the observed data. Put another way, the probability of the observed missingness pattern is related to the observed data values but unrelated to the missing data values. More formally, MAR data satisfy the following condition:

$$\text{Pr}\left(R=r|Y_{\text{obs}}=y_{\text{obs}},Y_{\text{mis}}={\tilde{y}}_{\text{mis}}\right)=Pr\left(R=r|Y_{\text{obs}}=y_{\text{obs}},Y_{\text{mis}}=y_{\text{mis}}^{*}\right)$$

for any two realizations ${\tilde{y}}_{\text{mis}}$ and $y_{\text{mis}}^{*}$. When data are MAR, the underlying distributions of the observed and missing data are the same given the values of the observed data (Example 1.13, Little & Rubin, 2019). The MAR assumption is powerful but often misunderstood (perhaps in part because of the term “random”). As with MCAR and MACAR, there is a stronger assumption whereby we enforce independence between Y and R given $Y_{\text{obs}}$, which is called missing-always-at-random, or MAAR (Mealli & Rubin, 2015).

The most general missing data assumption is MNAR. Data are MNAR if R depends on Y through $Y_{\text{mis}}$, the missing data. Put another way, the probability of missingness is related to the missing data values themselves. More formally, MNAR data satisfy the following condition:

(2) $$\text{Pr}\left(R=r|Y_{\text{obs}}=y_{\text{obs}},Y_{\text{mis}}={\tilde{y}}_{\text{mis}}\right)\;\neq \text{Pr}\left(R=r|Y_{\text{obs}}=y_{\text{obs}},Y_{\text{mis}}=y_{\text{mis}}^{*}\right)$$

for some values of ${\tilde{y}}_{\text{mis}}$ and $y_{\text{mis}}^{*}$ such that ${\tilde{y}}_{\text{mis}}\neq y_{\text{mis}}^{*}$. When data are MNAR, the underlying distributions of the observed and missing data are not the same. As with MACAR and MAAR, a stronger MNAR assumption implies missing-not-always-at-random, or MNAAR (Mealli & Rubin, 2015).

These missing data definitions are at first technical and esoteric, so making them more concrete is helpful to aid in their understanding. Distributions of hypothetical MCAR, MAR, and MNAR VMMI scores are shown in Figure 1. When VMMI scores are MCAR, the distributions of the observed and missing VMMI scores are the same. When VMMI scores are MAR given VT, the distributions of the observed and missing VMMI scores are the same within all woody wetlands, the same within all herbaceous wetlands, but different between woody and herbaceous wetlands. This pattern emerges because the probability of missingness is higher for woody wetlands than for herbaceous wetlands. Finally, when VMMI scores are MNAR, the distributions of the observed and missing VMMI scores are different for all wetlands, even after accounting for VT.

Unfortunately, assumptions about the missing data mechanisms are generally challenging, if not impossible, to evaluate directly (Schafer & Graham, 2002). Little (1988b) proposed a test to evaluate the plausibility of the MCAR assumption, while Enders (2022) reviews some approaches to compare MCAR and MAR assumptions. Van Buuren (2018) notes, however, that approaches that attempt to distinguish between MCAR, MAR, and MNAR assumptions are not widely used and their practical value is lacking.

The terms “ignorable” and “nonignorable” are important and are often used in the missing data literature. The missing data mechanism is called ignorable if the distribution Pr(R=r∣Y=y) can be safely ignored in our statistical model for Pr(Y=y). By Pr(Y=y), we colloquially mean the probability mass function (discrete Y) or the probability density function (continuous Y). MCAR and MAR data are ignorable because the mechanism yielding R does not depend on $Y_{\text{mis}}$, as long as the parameters that govern the missingness mechanism are distinct from the parameters that govern the data distribution (Little & Rubin, 2019). Intuitively, these parameters sets are distinct if they do not share information with one another; formally, these parameter sets are distinct if their joint parameter space is the product of their individual parameter spaces. It is important to recognize that the ignorable assumption simply implies that (after conditioning on the observed data) we can ignore the specific missingness mechanism, not that we can ignore the process of thoughtfully handling the missing data (Van Buuren, 2018). MNAR data are synonymously called nonignorable because the underlying missingness mechanism cannot be safely ignored in our statistical model (i.e., R and Y must be modeled together). Given that MCAR and MAR mechanisms are both ignorable, the distinction between them is often of little practical importance, as similar missing data approaches (e.g., imputation) can be used in either case. The distinction between ignorable and nonignorable missing data, however, is of critical importance (e.g., MCAR or MAR vs. MNAR).

### Directed acyclic graphs

As defined above, the definitions of MCAR, MAR, and MNAR can be somewhat inscrutable when applied to a given dataset. Even though each of the missingness mechanisms can be precisely defined in terms of the conditional distribution $\text{Pr}\left(R=r|Y_{\text{obs}}=y_{\text{obs}},Y_{\text{mis}}={\tilde{y}}_{\text{mis}}\right)$, it can be hard to interrogate whether these mechanisms are plausible given the setting and the data at hand. Directed acyclic graphs (DAGs) offer a way to define the reciprocal relationships between all variables in a missing data analysis using commonly understood graphical norms (Daniel et al., 2012; Mohan et al., 2013; Mohan & Pearl, 2021; Moreno-Betancur et al., 2018; Thoemmes & Mohan, 2015).

A DAG comprises nodes that represent random variables of interest and unidirectional arrows connecting these nodes to one another (Greenland et al., 1999; Morgan & Winship, 2014; Pearl, 2009a). The root of an arrow represents a cause, and the end of the arrow represents an effect of the cause. Crucially, these graphs cannot contain cycles, meaning that no node is a cause of itself. There is an exact correspondence between any DAG and the set of probability distributions encoding the conditional independence relationships represented by the DAG. These conditional independence relationships can be determined via a graphical criterion called d-separation (Pearl, 2009b). Another way to think about this correspondence is via nonparametric structural equation models (SEMs; Morgan & Winship, 2014). Traditional SEMs are a set of equations defining random variables (typically represented as uppercase letters) as linear functions of other variables and noise terms (typically represented as lowercase e subscripted by the random variable with which it is associated), whereas nonparametric SEMs do not restrict the set of equations to be linear (Kline, 2023; Morgan & Winship, 2014).

To make these concepts more concrete, we continue with our running example of missingness in VMMI, which we measure with the binary variable $R_{\text{VMMI}}$. Recall that $R_{\text{VMMI}}$ takes a value of 1 if VMMI is observed and 0 if VMMI is missing. All potential missingness mechanisms are shown in Figure 2. The graphs shown in Figure 2 offer clear contrasts, notably through which arrows are omitted when moving from MNAR to MAR to MCAR. The MCAR, MAR, and MNAR DAGs all encode the following functional relationship:

$$\text{VMMI}=f_{\text{VMMI}}\left(\text{VT},\text{SH},e_{\text{VMMI}}\right),$$

where $f_{\text{VMMI}}$ and $f_{R_{\text{VMMI}}}$ are unspecified functions associated with each outcome variable, and $e_{\text{VMMI}}$ and $e_{R_{\text{VMMI}}}$ are random variables with unspecified distributions. However, MCAR, MAR, and MNAR DAGs all have different distributions for $R_{\text{VMMI}}$:

$$R_{\text{VMMI}}(\text{MCAR})=f_{R_{\text{VMMI}}}\left(e_{R_{\text{VMMI}}}\right)$$

$$R_{\text{VMMI}}(\text{MAR})=f_{R_{\text{VMMI}}}\left(\text{VT},e_{R_{\text{VMMI}}}\right)$$

$$R_{\text{VMMI}}(\text{MNAR})=f_{R_{\text{VMMI}}}\left(\text{VMMI},\text{VT},e_{R_{\text{VMMI}}}\right)$$

While these equations are somewhat complex, the graphical depictions of these relationships offer a clearer way to communicate and critique their implicit assumptions (Mohan & Pearl, 2021).

One benefit of using a DAG to represent a missing data problem is that, given only the DAG, one can determine whether the quantity of interest is estimable under any distribution adhering to the DAG without bias from large-sample fully observed data—Mohan and Pearl (2021) call this property “recoverability” (i.e., nonparametric identifiability). Because DAGs express dependence relationships only and do not encode assumptions about the nature of those relationships (e.g., linear with Gaussian errors), these results will not depend on any functional or distributional assumptions. In order to determine whether a quantity is recoverable, one must verify that the observation indicator, R, is d-separated (i.e., conditionally independent) from the partially observed variables given the fully observed variables. This is equivalent to MAAR as defined above; it is important to keep in mind that missing data mechanisms as derived from DAGs correspond to the “always” versions of MCAR, MAR, and MNAR, or MACAR, MAAR, and MNAAR. This is because DAGs represent a collection of random variables rather than any particular realization of random variables.

Mohan and Pearl (2021) refer to MAR in the DAG framework as v-MAR to distinguish it from MAR, but we stick to MAR within this section for consistency. DAG recoverability results are algebraic, rather than statistical, so they have relatively few assumptions; thus, many practitioners turn to inverse probability weighting and doubly robust approaches to parameter estimation; we explore these techniques in detail in Other missing data approaches. Surprisingly, Mohan and Pearl (2021) show that there are examples of MNAR missingness that are learnable from complete-data units. The validity of the estimated quantity, however, requires that the dataset at hand has been generated from the posited DAG; any deviations therefrom can introduce bias. Furthermore, an analysis that is too narrowly focused on bias can risk ignoring variance, which may outweigh the benefits of reducing bias (e.g., the bias-variance trade-off; see Hastie et al. (2009)).

DAGs are a useful complement to the missing data framework explored previously The graphical displays corresponding to DAGs are helpful in framing missing data problems and can aid in communicating the dependence assumptions in an analysis. DAGs have also recently been used in a predictive capacity (Piccininni et al., 2020). To the extent that researchers are uncomfortable making assumptions about, say, linearity or specific distributions, DAGs offer an algorithm to determine whether quantities of interest are learnable from complete data only. Despite this benefit, DAGs are limited in that they do not suggest functional or parametric restrictions to make a non-recoverable quantity recoverable aside from changing the DAG itself (Heckman & Pinto, 2022; Imbens, 2020). Furthermore, DAGs are not a substitute for the distinctions between MCAR, MAR, and MNAR; these terms still apply. Thus, DAGs are not a substitute for missing data mechanisms or the techniques and algorithms that follow, but rather can clarify the relationships between variables in missing data analyses.

### Meaningful and nonmeaningful missingness

Little and Rubin (2019) make the following assumption when describing missing data: “Missing data are unobserved values that would be meaningful for analysis if observed; in other words, a missing value hides a meaningful value.” So far, we have assumed missing data have hidden, meaningful values, but this is not always true. Consider the following example.

Wetlands, because of their diverse makeup and hydrologic regimes across the conterminous United States (Mitsch et al., 2023), do not always have surface water. When wetlands have surface water, water chemistry variables like nitrogen and phosphorous can be sampled. When wetlands do not have surface water, however, these water chemistry variables cannot be sampled, as there is no basis by which a water chemistry variable can be measured. If an item corresponding to a water chemistry variable is missing, it is important to know whether surface water is present at the wetland. If surface water is present, a measurement does exist, though its value is hidden; thus the missing item is meaningful for analysis. If surface water is not present, however, no measurement can exist, and the missing item is nonmeaningful.

The distinction between meaningful and nonmeaningful missingness has received little formal attention in the ecological literature but exists in other disciplines. In causal inference studies aimed at measuring a survivor average treatment effect, whereby one wishes to measure the impact of a treatment on, say, blood pressure, this measurement is only meaningful if patients are not deceased (Frangakis & Rubin, 2002; Hudgens & Halloran, 2006; Tchetgen Tchetgen, 2014; Wang et al., 2017). In social sciences, nonmeaningful missingness is sometimes called “out of scope” of the population of interest (Schafer & Graham, 2002). Henceforth, we assume that the missing data approaches we discuss relate to meaningful missingness, though we briefly return to methods for characterizing nonmeaningful missingness in Contingency variables.

## AN OVERVIEW OF MISSING DATA APPROACHES

Throughout this section, we provide an introduction to several methods for handling missing data. Generally, these methods rely on a MCAR or MAR (i.e., ignorable) assumption in order to perform adequately (i.e., yield unbiased, efficient parameter estimates with correct CI coverage). We focus on methods specifically designed for MNAR (i.e., nonignorable) data in Specific approaches for MNAR data.

### Preventing and recording missing data

While not a missing data approach per se, it is helpful to consider ways to prevent and record missing data when designing a study (Little et al., 2012). Suggestions include simplifying data collection protocols (De Leeuw, 2001; Lin et al., 2012; O’neill & Temple, 2012), conducting pilot studies to identify sources of missing data (Kang, 2013), and mitigating sources of bias in assessment instruments (Terando et al., 2017). Missing data should also be recorded in a clear consistent manner that cannot be confused with a valid value (Fraser et al., 2009; Pearson, 2006). For example, it may be confusing to assign a value of zero to a missing item when this scenario cannot be distinguished from an observed item with a value of zero. In addition to recording whether an item is missing, it can be beneficial to also record the reason for missingness (if available), as this may inform subsequent handling of the missing data. So-called planned missing data designs explicitly build missing data into studies, often to balance variables that are easy (or cheap) to observe with others that are difficult (or expensive) to observe (for some commentary, see Gelman et al., 1998; Graham, 2012; Graham et al., 2006; Hossie et al., 2021; Noble & Nakagawa, 2021). Planned missing data designs are closely related to double sampling designs (Little & Rubin, 2019). In a double sampling design, a probability sample is selected and basic variables are collected. Then, a probability subsample is selected from the initial sample and additional variables are collected.

### Complete case analysis and related approaches

The first missing data approach we detail is CCA (i.e., listwise deletion, complete records analysis). CCA is an intuitive approach that discards units with at least one missing item prior to analysis. Units 2 and 6 from $y_{\text{obs}}$ in Equation (1) have missing VMMI items, so the relevant CCA subset is:

$$y_{\text{CCA}}=\left[\begin{array}{cccc} \text{Unit} & \text{VT} & \text{SH} & \text{VMMI}\\ 1 & \text{Herbaceous} & 90.4 & 39.4\\ 3 & \text{Woody} & 97.5 & 44.0\\ 4 & \text{Woody} & 79.6 & 25.0\\ 5 & \text{Herbaceous} & 33.6 & 41.2 \end{array}\right].$$

CCA is simple and straightforward but completely omits partially observed (and potentially useful) data. When data are MCAR, CCA yields unbiased (i.e., correct on average) parameter estimates and SEs appropriate for the reduced sample size (Van Buuren, 2018). When data are MAR or MNAR, CCA often yields biased parameter estimates and SEs; an example is when variables related to missingness and correlated with missing values are omitted from the analysis (Little & Rubin, 2019; Schafer & Graham, 2002). There are some scenarios, however, where CCA still yields unbiased estimators and appropriate SEs for MAR or MNAR data; an example is a linear regression with fully observed explanatory variables and a partially observed response variable (Little et al., 2024). CCA is attractive especially when the proportion of missing data is small, but Little and Rubin (2019) and Vach (2012) discourage the use of heuristic rules that determine whether CCA is appropriate, arguing that decisions be made uniquely for each dataset based on relevant context.

Available case analysis (ACA) is closely related to CCA. In ACA, only variables relevant to a specific analysis are kept, which can increase sample sizes for that analysis (Little & Rubin, 2019). Additionally, ACA can leverage correlations (i.e., covariances) among complete cases to inform analysis (this is sometimes called pairwise deletion). Little (1992) describes some challenges with ACA, namely that uncertainty quantification has some undesirable properties. ACA is intuitive but can be cumbersome when a dataset is used for many separate analyses (in which case ACA has to be repeatedly applied in varying contexts). This makes it difficult to compare SEs across analyses with varying sample sizes (Schafer & Graham, 2002).

### Imputation

Imputation methods replace (i.e., fill in, impute) missing items in Y with plausible values called imputations. Imputations are informed by $Y_{\text{obs}}$, and together, $Y_{\text{obs}}$ and the imputations yield a complete dataset that consists of observed and imputed items. Imputed items can be constructed in a deterministic (i.e., nonrandom) or stochastic (i.e., random) manner. Deterministic imputation methods always return the same set of imputations, while stochastic imputation methods build in some component of randomness into the imputation process (D’Agostino McGowan et al., 2024). Completing the dataset with a single set of random, imputed items is called “single imputation.” Single imputation methods sometimes incorporate additional randomness in the parameters of underlying models used to generate the imputations themselves, which we discuss shortly. After imputation, the complete dataset can be analyzed (e.g., using a linear regression model) as if there were no missing items.

The method (i.e., model, technique) used to generate imputations is called the imputation method. The method used to analyze the complete dataset is called the analysis method. Often, imputation and analysis methods have the same form (i.e., are linear regression models), but this is not required. For example, a binary variable could be imputed using a logistic regression model and then used as an explanatory variable in a linear regression model.

Shortly we review several imputation methods, detailing the benefits and drawbacks of each while revisiting $y_{\text{obs}}$ in Equation (1). Throughout, we first assume that items are missing only for a single variable, and then we generalize to settings where several variables have missing items. We discuss “multiple imputation,” whereby complete datasets from separate single imputation draws are pooled together, sharing information (Rubin, 1996). Multiple imputation is compared and contrasted against CCA, deterministic imputation, and single imputation in Figure 3.

### Mean imputation

A simple imputation method is a deterministic method called mean imputation (i.e., unconditional mean imputation). In mean imputation, a variable’s missing items are imputed as the mean of its observed items (Wilks, 1932). Imputing VMMI items two and six from $y_{\text{obs}}$ yields

$$y_{\text{Mean}}=\left[\begin{array}{cccc} \text{Unit} & \text{VT} & \text{SH} & \text{VMMI}\\ 1 & \text{Woody} & 97.5 & 44.0\\ 2 & \text{Herbaceous} & 29.5 & 37.4\\ 3 & \text{Herbaceous} & 90.4 & 39.4\\ 4 & \text{Woody} & 79.6 & 25.0\\ 5 & \text{Herbaceous} & 33.6 & 41.2\\ 6 & \text{Woody} & 65.9 & 37.4 \end{array}\right],$$

where the VMMI imputations (in bold) equal 37.4, the mean of the four observed VMMI items: 44.0, 39.4, 25.0, and 41.2. A benefit of mean imputation is that the mean of the observed items equals the mean of the imputed items. Mean imputation is also quite computationally efficient. Unfortunately, mean imputation tends to artificially inflate the strength of the relationships in the data, leading to biased parameter estimates and notably underestimated variances (Gleason & Staelin, 1975; Kromrey & Hines, 1994; Olinsky et al., 2003). Enders (2022) claims that “in no situation is mean imputation defensible, and you should absolutely avoid this approach.”

### Regression imputation

The next deterministic imputation method is regression imputation (i.e., conditional mean imputation). In regression imputation, a linear regression model is first fit using the completely observed units. Then, the fitted model is used to generate an imputation for each missing item (Buck, 1960). Suppose we generate a linear regression model that captures the effect of VT and SH on VMMI. This model is written as:

(3) $${\text{VMMI}}_{i}=\beta_{0}+\beta_{1}ℐ\left({\text{VT}}_{i}\right)+\beta_{2}{\text{SH}}_{i}+\epsilon_{i},$$

where i indexes each of the four completely observed units (i=1,3,4,5), $\beta_{0}$ represents the overall intercept, $\beta_{1}$ represents the shift in average VMMI for woody wetlands,

$$ℐ\left({\text{VT}}_{i}\right)=\left\{\begin{array}{ll} 1 & \text{if}\;{\text{VT}}_{i}=\text{Woody}\\ 0 & \text{if}\;{\text{VT}}_{i}=\text{Herbaceous} \end{array},\right.$$

$\beta_{2}$ represents the change in average VMMI for a unit change in SH score, and each $\epsilon_{i}$ are independent and identically distributed (i.e., i.i.d.) random errors with a mean of zero and a (positive) variance of $\sigma^{2}$. Fitting the model in Equation (3) using ordinary least squares yields the following parameter estimates: ${\hat{\beta }}_{0}=36.142;{\hat{\beta }}_{1}=-7.581$; and ${\hat{\beta }}_{2}=0.067$. These fitted values from the model are combined with the VT and SH measurements for units 2 and 6 to generate the VMMI imputations:

$${\text{VMMI-IMP}}_{2}=36.142-7.581(0)+0.067(29.5)=38.12\;\text{and}$$

$${\text{VMMI-IMP}}_{6}=36.142-7.581(1)+0.067(65.9)=32.98$$

Then, the complete dataset is

$$y_{\text{Reg}}=\left[\begin{array}{cccc} \text{Unit} & \text{VT} & \text{SH} & \text{VMMI}\\ 1 & \text{Woody} & 97.5 & 44.0\\ 2 & \text{Herbaceous} & 29.5 & 38.1\\ 3 & \text{Herbaceous} & 90.4 & 39.4\\ 4 & \text{Woody} & 79.6 & 25.0\\ 5 & \text{Herbaceous} & 33.6 & 41.2\\ 6 & \text{Woody} & 65.9 & 33.0 \end{array}\right],$$

While intuitive, regression imputation, like mean imputation, tends to artificially inflate the strength of the relationships in the data, biasing parameter estimates and notably underestimating variances (Gleason & Staelin, 1975; Kromrey & Hines, 1994; Little & Rubin, 2019; Olinsky et al., 2003). Because its intuitive appeal is met with lackluster performance, Van Buuren (2018) claims that “Regression imputation, as well as its modern incarnations in machine learning, is probably the most dangerous of all [missing data] methods described here [in this book].”

### Stochastic regression imputation

Stochastic regression imputation is a single imputation method that builds upon regression imputation by adding a random (i.e., stochastic) error to each regression imputation. The random error is simulated from an appropriate error distribution (often, a Gaussian distribution) with mean zero and variance $\sigma^{2}$. Suppose that we simulate two i.i.d. random errors from the Gaussian distribution with mean zero and variance 174.14 (the value 174.14 is the estimated residual variance from the fitted regression model). Further suppose that the random error realized for unit 2 is −11.0 while the random error realized for unit 6 is 21.1. The stochastic regression imputations are then:

$${\text{VMMI-IMP}}_{2}=36.142-7.581(0)+0.067(29.5)-11.83\;=26.29\;\text{and}$$

$${\text{VMMI-IMP}}_{6}=36.142-7.581(1)+0.067(65.9)+21.14\;=54.12.$$

The complete dataset is

$$y_{\text{StReg}}=\left[\begin{array}{cccc} \text{Unit} & \text{VT} & \text{SH} & \text{VMMI}\\ 1 & \text{Woody} & 97.5 & 44.0\\ 2 & \text{Herbaceous} & 29.5 & 26.3\\ 3 & \text{Herbaceous} & 90.4 & 39.4\\ 4 & \text{Woody} & 79.6 & 25.0\\ 5 & \text{Herbaceous} & 33.6 & 41.2\\ 6 & \text{Woody} & 65.9 & 54.1 \end{array}\right].$$

While initially seeming counterintuitive, stochastic regression imputation tends to perform much better than regression imputation because stochastic regression imputation incorporates uncertainty in the imputations themselves (Little & Rubin, 2019). Unfortunately, stochastic regression imputation is not perfect and ignores uncertainty in estimated model parameters, often yielding SEs that are too small and CIs that are too narrow.

### Bootstrap stochastic regression imputation

Bootstrap stochastic regression imputation (henceforth called bootstrap imputation) is a single imputation method that builds upon stochastic regression imputation by incorporating an additional source of randomness associated with the parameter estimates (Efron, 1994). Borrowing bootstrap resampling ideas (Efron & Tibshirani, 1994), bootstrap imputation applies stochastic regression imputation to a random, resampled set of observed data (i.e., a bootstrap sample) instead of the observed data. Suppose the bootstrap sample contained two copies of item one, zero copies of item three, one copy of item four, and one copy of item five:

$$y_{\text{BSample}}=\left[\begin{array}{cccc} \text{Unit} & \text{VT} & \text{SH} & \text{VMMI}\\ 1 & \text{Herbaceous} & 90.4 & 39.4\\ 1 & \text{Herbaceous} & 90.4 & 39.4\\ 4 & \text{Woody} & 79.6 & 25.0\\ 5 & \text{Herbaceous} & 33.6 & 41.2 \end{array}\right].$$

The ordinary least squares parameter estimates for this model are ${\hat{\beta }}_{0}=42.265,{\hat{\beta }}_{1}=-14.742$, and ${\hat{\beta }}_{2}=-0.032$ and the random errors realized are 5.14 and −2.76, respectively. Together, they yield the bootstrap imputations:

$${\text{VMMI-IMP}}_{2}=42.265-14.742(0)-0.032(29.5)+5.14\;=46.47\;\text{and}$$

$${\text{VMMI-IMP}}_{6}=42.265-14.742(1)-0.032(65.9)-2.76\;=22.67.$$

Then, the complete dataset is

$$y_{\text{Boot}}=\left[\begin{array}{cccc} \text{Unit} & \text{VT} & \text{SH} & \text{VMMI}\\ 1 & \text{Woody} & 97.5 & 44.0\\ 2 & \text{Herbaceous} & 29.5 & 46.5\\ 3 & \text{Herbaceous} & 90.4 & 39.4\\ 4 & \text{Woody} & 79.6 & 25.0\\ 5 & \text{Herbaceous} & 33.6 & 41.2\\ 6 & \text{Woody} & 65.9 & 22.7 \end{array}\right],$$

Because bootstrap imputation also incorporates parameter uncertainty, it can yield estimates with appropriate SEs and CI coverage (Little & Rubin, 2019; Schomaker & Heumann, 2018). Other imputation methods that borrow resampling ideas are Jackknife imputation (Miller, 1974) and Bayesian imputation (Box & Tiao, 2011; Rubin, 2004).

### Hot deck imputation

Several of the aforementioned imputation methods use a statistical model to generate imputations. A fundamentally different perspective is to use hot deck imputation (Kalton & Kasprzyk, 1986; Myers, 2011; Scheuren, 2005). Hot deck imputation imputes missing items by matching (i.e., copying) the value of an observed item from an appropriate donor unit. Donors are typically selected from the observed data, though it is possible to select from alternative data sources. Selecting donors from alternative data sources is called cold deck imputation. Cold deck imputation is far less common than hot deck imputation, in large part because finding suitable alternative data sources can be challenging. Because hot deck imputations match observed items, they are constrained within the range of the observed data. They avoid strong parametric assumptions and tend to be robust to data transformations like logarithms or exponentiation. However, they do not always perform well with small sample sizes or highly skewed data (Kleinke, 2017).

Hot deck imputation involves two steps. The first step is to define a closeness measure between the item requiring imputation and all other items (Andridge & Little, 2010). Suppose that for y, we define a closeness measure that (1) treats units with the same vegetation type as closer than units with different vegetation types and (2) after accounting for vegetation type, treats units with more similar soil hardening scores (measured by absolute deviation between the scores) as closer than units with more different soil hardening scores. In this example, unit 2 is closest to unit 5 because it has the same vegetation type and a more similar SH score than unit 3; and unit 6 is closest to unit 4 because it has the same vegetation type and a more similar SH score than unit 1.

The second hot deck imputation step is to choose a close donor to generate the imputation. A hot deck deterministic imputation method called nearest neighbor always selects the closest donor to generate the imputation (Chen & Shao, 2000; Rancourt et al., 1994). Using the aforementioned closeness measure for $y_{\text{obs}}$ and nearest neighbor, the complete dataset is

$$y_{\text{NN}}=\left[\begin{array}{cccc} \text{Unit} & \text{VT} & \text{SH} & \text{VMMI}\\ 1 & \text{Woody} & 97.5 & 44.0\\ 2 & \text{Herbaceous} & 29.5 & 41.2\\ 3 & \text{Herbaceous} & 90.4 & 39.4\\ 4 & \text{Woody} & 79.6 & 25.0\\ 5 & \text{Herbaceous} & 33.6 & 41.2\\ 6 & \text{Woody} & 65.9 & 25.0 \end{array}\right],$$

The imputed VMMI value for unit 2 matches the VMMI value for unit 5 (41.2), and the imputed VMMI value for unit 6 matches the imputed value for unit 4 (25.0). While intuitive and straightforward, nearest neighbor fails to incorporate randomness in the closeness measure or donor selection and hence performance suffers.

A widely used hot deck single imputation method is predictive mean matching (Little, 1988a; Rubin, 1986). Predictive mean matching finds the d donors closest donors and randomly selects one to generate the imputation. Using the aforementioned closeness measure for $y_{\text{obs}}$ and predictive mean matching with d=2, a potential complete dataset is

$$y_{\text{PMM}}=\left[\begin{array}{cccc} \text{Unit} & \text{VT} & \text{SH} & \text{VMMI}\\ 1 & \text{Woody} & 97.5 & 44.0\\ 2 & \text{Herbaceous} & 29.5 & 39.4\\ 3 & \text{Herbaceous} & 90.4 & 39.4\\ 4 & \text{Woody} & 79.6 & 25.0\\ 5 & \text{Herbaceous} & 33.6 & 41.2\\ 6 & \text{Woody} & 65.9 & 25.0 \end{array}\right],$$

where the VMMI value of unit 3 was chosen randomly over the VMMI value of unit 5 (for the VMMI imputation of unit 2) and the VMMI value of unit 4 was chosen randomly over the VMMI value of unit 1 (for the VMMI imputation of unit 6).

Predictive mean matching closeness can be determined using various rules. Two such rules are Type-0 matching, which uses a deterministic approach to determine closeness, and Type-1 matching, which uses an approach to determine closeness that has randomness (Van Buuren, 2018; White et al., 2011). We provide further details regarding Type-0 and Type-1 matching in Appendix S2: Section S1.

Generally, closeness rules that incorporate randomness are preferred to closeness rules that are deterministic (Van Buuren, 2018). Van Buuren (2018) recommends choosing d to be 3, 5, or 10, while Schenker and Taylor (1996) use an adaptive method to select d. Siddique and Belin (2008) discuss weighting among the d donors so that closer donors are more likely to be selected. Predictive mean matching has been shown to perform well for a variety of data types and missing data scenarios (Kleinke, 2017; Marshall et al., 2010; Morris et al., 2014; Vink et al., 2014).

### Multiple imputation

Singly imputed data (i.e., a complete dataset from a single imputation draw) represent one of many possible imputed datasets and hence fail to capture all the variability in the imputations themselves. Using singly imputed data in a subsequent analysis tends to yield parameter estimates whose corresponding SEs are too small and confidence errors too narrow, regardless of the imputation method used (Little & Rubin, 2019). To address this drawback, Rubin (1977) proposed a groundbreaking approach called multiple imputation. In multiple imputation, several singly imputed datasets are generated, an analysis is performed on each, and the results are appropriately pooled (Rubin, 1996). While the general concept behind multiple imputation is simplistically elegant, there are several important yet subtle questions lurking below the surface. Some examples are as follows: “How many imputed datasets should be used?”; “How should the results from different imputed datasets be pooled?”; and “Can the impact of missingness on an analysis be measured?” We explore answers to these types of questions next.

Rubin’s Rules (Rubin, 2004) provide a framework for using multiple imputation to pool results from an analysis method applied to the m singly imputed datasets. Let Q represent a parameter requiring estimation, which could be a simple mean, a slope parameter in a regression model, a population total, or some other quantity of interest. Suppose that an appropriate analysis of each of the m singly imputed datasets yields an estimate of Q, denoted by ${\hat{Q}}_{i}(i=1,2,\ldots ,m)$. Rubin’s Rules state that the multiple imputation estimator of Q, denoted $\hat{Q}$, is given by

(4) $$\hat{Q}=\frac{1}{m}\sum_{i=1}^{m}{\hat{Q}}_{i}.$$

Equation (4) plainly states that $\hat{Q}$ is the average of each ${\hat{Q}}_{i}$. While the estimator $\hat{Q}$ is simple and intuitive, calculating its variance is more challenging. Rubin’s Rules state that the variance of $\hat{Q}$, denoted $\hat{T}$, is given by

$$\hat{T}=\frac{1}{m}\sum_{i=1}^{m}{\hat{U}}_{i}+\left(1+\frac{1}{m}\right)\sum_{i=1}^{m}\frac{{\left({\hat{Q}}_{i}-\hat{Q}\right)}^{2}}{m-1}\;=\text{Var}(\text{Within})+\text{Var}(\text{Between})$$

where ${\hat{U}}_{i}$ is an estimate of the variance of ${\hat{Q}}_{i}$. The term $\frac{1}{m}\sum_{i=1}^{m}{\hat{U}}_{i}$ represents the variability within each ${\hat{Q}}_{i}$ while the term $\left(1+\frac{1}{m}\right)\sum_{i=1}^{m}\frac{{\left({\hat{Q}}_{i}-\hat{Q}\right)}^{2}}{m-1}$ represents the variability between each ${\hat{Q}}_{i}$. Henceforth, we call these terms Var(Within) and Var(Between), respectively.

Often $\hat{Q}$ is a sum of random quantities (e.g., a mean, a regression slope, a total). In this context, it is reasonable, based on the central limit theorem (Casella & Berger, 2024), to assume that $\hat{Q}$ is approximately normally distributed with mean Q and variance T. Under general conditions, this assumption implies that the test statistic

$$t_{v}^{*}=\frac{\hat{Q}-Q}{\sqrt{\hat{T}}}$$

is approximately t distributed with v df. Thus, p values for appropriate hypothesis tests can be obtained by comparing $t_{v}^{*}$ to the t-reference distribution. Moreover, a (1-α)% CI is given by

$$\left(\hat{Q}-t_{v,1-\alpha /2}\sqrt{\hat{T}},\hat{Q}+t_{v,1-\alpha /2}\sqrt{\hat{T}}\right),$$

where $t_{v,1-\alpha /2}$ is the critical value from the t-reference distribution. The df for these hypothesis tests and CIs depend on the number of singly imputed datasets (m) and the sample size within each imputed dataset. Various approaches to determining df in the presence of missing data are provided by Barnard and Rubin (1999), Lipsitz et al. (2002), Reiter (2007), and Wagstaff and Harel (2011).

Rubin (2004) derives two statistics that elucidate the impact missing data have on an analysis. The first statistic is the proportion of variability in $\hat{Q}$ that results from the missing data, denoted by λ. An estimate of λ, denoted by $\hat{\lambda }$, is given by

$$\hat{\lambda }=\frac{\text{Var}(\text{Between})}{\hat{T}},$$

the ratio of the between-imputation variability to the total variability. The statistic λ varies from zero to one; larger values indicate more variability in $\hat{Q}$ is attributable to the missing data. For example, a value of 0.5 implies that half of the variability in $\hat{Q}$ is attributable to the missing data.

The second statistic is the relative increase in variability in $\hat{Q}$ due to the missing data, denoted by r. An estimate of r, denoted by $\hat{r}$, is given by

$$\hat{r}=\frac{\text{Var}(\text{Between})}{\text{Var}(\text{Within})},$$

the ratio of the between-imputation variability to the within-imputation variability. For example, a value of one implies that the between-imputation is the same as the within-imputation variability. If half the variability is between-imputation variability, then roughly half the variability in $\hat{Q}$ is attributable to the missing data. This perspective reveals a connec-tion between $\hat{\lambda }$ and $\hat{r}$, and it can be shown that $\hat{r}=\hat{\lambda }/(1-\hat{\lambda })$. Rubin (2004) discusses other statistics that capture different aspects of imputation-related variability.

One reason the magnitude of between-imputation variability is important is because it can inform the choice of m, the number of singly imputed datasets. Choosing an appropriate m is a balance between performance and computational feasibility. As m increases, multiple imputation becomes more reliable, but the benefits rapidly decrease with large m (Rubin, 2004). Moreover, storing and analyzing m singly imputed datasets can be computationally cumbersome. Van Buuren (2018) mentions that classical advice is to set m to three, four, or five when there are low-to-moderate amounts of missing data and that substantive conclusions, especially when the primary interest is point estimates, are unlikely to change significantly as a result of raising m beyond five. Rubin (2004), Schafer (1997), and Schafer and Olsen (1998) argue that using m greater than 10 is rarely necessary. Bodner (2008) and Graham et al. (2007), however, recommend larger m; they advise setting m=50 when λ≤0.5 and m≥100 when λ≥0.7. White et al. (2011) provide a simple rule to set m≥100λ. For example, if approximately 34% of the data is missing, set m≥34. One way to balance all these perspectives is use m=100 as a default, adjusting as necessary depending on context.

### Missing data in more than one variable

So far, we have only discussed missing items in a single variable, but in many practical applications, items are missing in several variables simultaneously. Sometimes, missing items have a specific configuration that facilitates their handling (Little & Rubin, 2019), though this is not true in general. Suppose $y_{\text{obs}}$ in Equation (1) now also has missing items in the VT and SH variables and looks like

(5) $$y_{\text{obs}}=\left[\begin{array}{cccc} \text{Unit} & \text{VT} & \text{SH} & \text{VMMI}\\ 1 & \text{Woody} & 97.5 & 44.0\\ 2 & * & 29.5 & *\\ 3 & \text{Herbaceous} & 90.4 & 39.4\\ 4 & \text{Woody} & 79.6 & 25.0\\ 5 & \text{Herbaceous} & * & 41.2\\ 6 & \text{Woody} & 65.9 & * \end{array}\right],$$

which has missing items for all three variables. Fortunately, imputation methods naturally extend to accommodate missing items in several variables. For example, mean imputation applies a straightforward extension—simply take the mean of each variable. For categorical variables, one approach is to impute using the most common category (i.e., level).

Generally, however, imputation methods are challenging to extend to the multivariable case because they must account for relationships that exist among several variables. There are two main approaches to this extension: the joint approach and the conditional approach. In the joint approach, imputations for all missing items are generated simultaneously (Little & Rubin, 2019). Schafer (1997) uses a joint approach for data that are multivariate Gaussian, and Demirtas et al. (2008) show that even when this Gaussian assumption is violated, the method tends to work well. Allison (2005) describes a joint approach for data that are categorical.

The conditional approach leverages conditioning to capture relationships among variables instead of specifying a multivariate distribution. The conditional approach has many names (see Van Buuren, 2018, for a review) but is commonly called the fully conditional specification (FCS, Van Buuren et al., 2006) or chained equations (Azur et al., 2011; White et al., 2011). We adopt the chained equations nomenclature henceforth. In chained equations, univariate distributions are assumed for each variable that condition on the remaining variables. After determining suitable initial values for each missing item, missing items are updated with new imputations one variable at a time. Starting with $y_{\text{obs}}$ from Equation (5), we can use chained equations to impute a complete dataset. First, we use mean imputation to determine initial values:

$$\left[\begin{array}{cccc} \text{Unit} & \text{VT} & \text{SH} & \text{VMMI}\\ 1 & \text{Woody} & 97.5 & 44.0\\ 2 & \text{Woody} & 29.5 & 37.4\\ 3 & \text{Herbaceous} & 90.4 & 39.4\\ 4 & \text{Woody} & 79.6 & 25.0\\ 5 & \text{Herbaceous} & 72.6 & 41.2\\ 6 & \text{Woody} & 65.9 & 37.4 \end{array}\right].$$

Second, we assume the following relationship for VMMI given VT and SH,

$$[\text{VMMI}|\text{VT},\text{SH}]\sim \text{Gaussian}\left(\mu_{1},\sigma_{1}^{2}\right)$$

$$\mu_{1}\equiv \beta_{0,1}+\beta_{1,1}ℐ(\text{VT})+\beta_{2,1}\text{SH}$$

$$ℐ(\text{VT})=\left\{\begin{array}{ll} 1 & \text{if VT}=\text{Woody}\\ 0 & \text{if VT}=\text{Herbaceous} \end{array},\right.$$

and use stochastic regression imputation to update VMMI imputations:

$$\left[\begin{array}{cccc} \text{Unit} & \text{VT} & \text{SH} & \text{VMMI}\\ 1 & \text{Woody} & 97.5 & 44.0\\ 2 & \text{Woody} & 29.5 & 42.1\\ 3 & \text{Herbaceous} & 90.4 & 39.4\\ 4 & \text{Woody} & 79.6 & 25.0\\ 5 & \text{Herbaceous} & 72.6 & 41.2\\ 6 & \text{Woody} & 65.9 & 24.7 \end{array}\right].$$

The distributional notation [VMMI|VT, SH] simply means the distribution of VMMI given VT and SH. Third, we use nearest neighbor hot deck (determining closeness using VT and breaking ties with VMMI) to update SH imputations:

$$\left[\begin{array}{cccc} \text{Unit} & \text{VT} & \text{SH} & \text{VMMI}\\ 1 & \text{Woody} & 97.5 & 44.0\\ 2 & \text{Woody} & 29.5 & 42.1\\ 3 & \text{Herbaceous} & 90.4 & 39.4\\ 4 & \text{Woody} & 79.6 & 25.0\\ 5 & \text{Herbaceous} & 90.4 & 41.2\\ 6 & \text{Woody} & 65.9 & 24.7 \end{array}\right].$$

Fourth, we assume the following relationship for VT given SH and VMMI,

$$[\text{VT}|\text{SH},\text{VMMI}]\sim \text{Bernoulli}\left(\mu_{2}\right)$$

$$\mu_{2}\equiv \text{Probability that VT}=\text{Woody}$$

$$\text{log}\;\left(\frac{\mu_{2}}{1-\mu_{2}}\right)=\beta_{0,2}+\beta_{1,2}\text{SH}+\beta_{2,2}\text{VMMI},$$

and use logistic regression (with a cutoff probability of 0.5) to update VT imputations:

$$\left[\begin{array}{cccc} \text{Unit} & \text{VT} & \text{SH} & \text{VMMI}\\ 1 & \text{Woody} & 97.5 & 44.0\\ 2 & \text{Woody} & 29.5 & 42.1\\ 3 & \text{Herbaceous} & 90.4 & 39.4\\ 4 & \text{Woody} & 79.6 & 25.0\\ 5 & \text{Herbaceous} & 90.4 & 41.2\\ 6 & \text{Woody} & 65.9 & 24.7 \end{array}\right].$$

Here, the VT imputation stayed “Woody.” Fifth, we use stochastic regression imputation to update VMMI imputations again:

$$\left[\begin{array}{cccc} \text{Unit} & \text{VT} & \text{SH} & \text{VMMI}\\ 1 & \text{Woody} & 97.5 & 44.0\\ 2 & \text{Woody} & 29.5 & 51.4\\ 3 & \text{Herbaceous} & 90.4 & 39.4\\ 4 & \text{Woody} & 79.6 & 25.0\\ 5 & \text{Herbaceous} & 90.4 & 41.2\\ 6 & \text{Woody} & 65.9 & 33.8 \end{array}\right].$$

This process continues iteratively until a suitable number of imputations have been updated, at which point the complete dataset may look like

$$\left[\begin{array}{cccc} \text{Unit} & \text{VT} & \text{SH} & \text{VMMI}\\ 1 & \text{Woody} & 97.5 & 44.0\\ 2 & \text{Herbaceous} & 29.5 & 47.2\\ 3 & \text{Herbaceous} & 90.4 & 39.4\\ 4 & \text{Woody} & 79.6 & 25.0\\ 5 & \text{Herbaceous} & 29.5 & 41.2\\ 6 & \text{Woody} & 65.9 & 17.6 \end{array}\right].$$

For multiple imputation, this process is repeated m times and the analysis results are appropriately pooled.

### Including the analysis method response variable in an imputation method

It is not intuitively clear whether or not the response variable for the analysis method should be included in the imputation method. D’Agostino McGowan et al. (2024) provide a straightforward recommendation: Include the response variable if the imputation method is stochastic (e.g., single or multiple imputation); do not include the response variable if the imputation method is deterministic (e.g., regression imputation). D’Agostino McGowan et al. (2024) introduce this argument by noticing the connections between stochastic imputation and Bayesian modeling, which both have random components. They argue that in Bayesian modeling, the response variable is conditioned upon, and hence it is natural to include the response variable in a stochastic imputation method. Deterministic imputation does not share this Bayesian connection and hence the response variable should be ignored by the imputation method. D’Agostino McGowan et al. (2024) support these intuitions using mathematically rigorous arguments that leverage expectations, variances, and covariances among variables of interest. They clarify that appropriate analysis method SEs can be obtained when using deterministic imputation via a resampling approach (i.e., jackknife, bootstrap), while appropriate analysis method SEs can be obtained when using single imputation via the Robins and Wang (2000) asymptotic variance estimator. Several other authors have noted the benefits of including the analysis method’s response variable as part of (i.e., to inform) the stochastic imputation method (Hughes et al., 2019; Kenward & Carpenter, 2007; Kontopantelis et al., 2017; Moons et al., 2006).

### Inclusive versus restrictive imputation strategies

Collins et al. (2001) make explicit two approaches to specifying variables in an imputation method: the inclusive approach and the restrictive approach. The inclusive approach includes all variables in an imputation method that are also in the analysis method, while the restrictive approach omits at least one variable from the imputation method that is in the analysis method. For example, if the analysis method is a linear regression of VMMI on VT and SH, then an inclusive imputation method for missing VMMI items includes VT and SH (as well as potentially other variables not in the analysis method), while a restrictive imputation method for missing VMMI items omits either VT or SH (or both).

The inclusive strategy is more likely (than the restrictive strategy) to capture associations and important causes of missingness among variables used in the imputation method. D’Agostino McGowan et al. (2024) argue that the inclusive approach implies “congeniality,” which means there is a unifying Bayesian model that embeds the imputation and analysis methods (Meng, 1994). D’Agostino McGowan et al. (2024) state that not only should the imputation method include all variables in the analysis method, but that they should be in the same form (see also Von Hippel, 2009). For example, if a logged version of a variable is used in the analysis method, a logged version of that variable should also be used in the imputation method. When the analysis method includes interactions, those interactions should also be in the imputation method (Tilling et al., 2016). When variables are included in the imputation method but not the analysis method, they are called auxiliary variables (Collins et al., 2001). Auxiliary variables can be useful components of an inclusive strategy when they are related to variables being imputed but not relevant to the analysis method’s goals. Collins et al. (2001) argue that the inclusive strategy is at worst neutral and at best extremely beneficial, and that it can even improve outcomes for MNAR data.

A drawback of the inclusive strategy is that if auxiliary variables are unrelated to the variable being imputed, they can decrease efficiency in resulting estimates, though Collins et al. (2001) argue this efficiency loss tends to be minimal. Thoemmes and Rose (2014) identified that the inclusive strategy can introduce “collider” variables (Morgan & Winship, 2014; Pearl, 2009a). A variable is a collider variable if its inclusion in an imputation method actually changes the missing data from MAR to MNAR, and hence makes methods that rely on MAR infeasible. Other authors, including Rubin (2009), believe that collider bias is less of a concern. Collins et al. (2001) clarify that adding additional variables to the imputation method should be done carefully.

The restrictive strategy, while simpler than the inclusive strategy, can omit variables that explain missingness and the variables being imputed. The restrictive strategy can yield severely biased estimates with inaccurate SEs and poor CI coverage (Collins et al., 2001). In general, if a variable is in the analysis method, it should also inform the imputations in the imputation method.

### Imputation diagnostics

Diagnostic tools are useful to evaluate the adequacy of a statistical procedure. In imputation, there are several scenarios in which diagnostics can be useful. The first scenario is examining diagnostics in the underlying imputation method. For example, regression imputation uses the observed data to create a regression model, which is later leveraged to generate imputations. This regression model can be checked using standard regression diagnostics like residual plots (Fox, 2019; Gelman et al., 2005; Montgomery et al., 2021). The second scenario is examining diagnostics of the analysis method applied to the complete dataset. Diagnostics in these scenarios are important but specific to the assumed models in the imputation and analysis methods. A third scenario, which we focus on, is examining distributions of the imputations themselves as compared to the observed data.

Several authors have provided overviews of helpful diagnostic tools that can be used to assess the adequacy of the imputations (Abayomi et al., 2008; Eddings & Marchenko, 2012; Nguyen et al., 2017; Su et al., 2011). Van Buuren (2018) notes that visualizations are particularly useful for identifying discrepancies between observed and imputed data that can occur in the means, spread, scales, or relationships among variables. These visualizations can include histograms or densities of individual variables or scatterplots between variables that are separated by observed and imputed classification; these visualizations can also be conditioned on other variables (Bondarenko & Raghunathan, 2016). One such visualization is shown in Figure 4, which compares observed and imputed data for mean imputation, regression imputation, and bootstrap imputation of a hypothetical variable $z_{2}$ using a hypothetical variable $z_{1}$. Diagnostics of this type are especially helpful for visualizing the general behavior of the observed data relative to the imputed data.

### Evaluating imputation methods

Recall that in The goals of a statistical analysis, we discussed that often, a goal of a statistical analysis is to appropriately estimate population quantities (e.g., population mean, regression coefficient) and characterize uncertainty in those estimates. Hence, imputation methods should be evaluated on their capacity to achieve these goals, often by yielding estimates that are unbiased and efficient with correct CI coverage. It may seem natural to instead evaluate imputation methods by comparing imputed items to their true values, but recovering missing items is typically not the primary analysis goal. Schafer and Graham (2002) state that “With or without missing data, the goal of a statistical procedure should be to make valid and efficient inferences about a population of interest—not to recover missing observations… A missing value treatment cannot be separated from the modeling, estimation, or testing procedure from which it is embedded,” while Van Buuren (2018) states that “measures based on similarity between the true and imputed values do not separate valid from invalid imputation methods.” Little and Rubin (2019), Murray (2018), and Yuan (2010), among others, make similar clarifications. In Appendix S2: Section S1, we provide a numerical example that shows how evaluating imputation methods by their ability to recover true missing items can be misleading when the statistical analysis goals are parameter estimation, prediction, and uncertainty quantification.

### Imputation versus prediction

Imputation and prediction are not the same. They are different techniques that satisfy different goals and should be evaluated differently. Sometimes, as nomenclature varies across disciplines, the terms imputation and prediction are used synonymously (Eskelson et al., 2009; Ver Hoef & Temesgen, 2013). We discourage this practice and believe that it is helpful to make explicit the important distinction between imputation and prediction. Imputation refers to the practice of imputing missing items from units that are part of the dataset used for to construct the analysis method. Prediction refers to the practice of constructing best guesses of random variables that are separate from the units in the analysis method. Imputations should reflect the tendencies (i.e., means, variances, correlations) among the variables in the observed data used by the analysis method. The analysis method should be used to construct predictions that are (1) as close as possible to the true value of the realized random variable being predicted (e.g., a best linear unbiased prediction), and (2) accompanied with an appropriate measure of uncertainty (e.g., a prediction interval).

For many missing data approaches, prediction is as straightforward as using the analysis method to make predictions as if there were no missing data. For example, CCA removes missing data before fitting the analysis method which can then be used to generate predictions. For single imputation, the complete dataset is used by the analysis method to generate predictions. For multiple imputation, however, there are many imputed datasets and predictions that must be combined in some way to generate a single, final prediction. Miles (2016) clarifies two distinct approaches to this task that are called “Predict-Combine” (PC) and “Combine-Predict” (CP). The PC approach uses the analysis method from each multiply imputed dataset to generate separate predictions and then combines them using Rubin’s Rules, while the CP approach averages parameters from each analysis method and then generates a single prediction. There are many scenarios where PC and CP yield very similar (or equivalent) predictions. While CP does have some computational benefits, we prefer PC because it adheres to Rubin’s Rules. Figure 5 helps to elucidate the subtle distinction between PC and CP.

### Data augmentation

Unlike imputation, which separates the handling of missing data from the subsequent analysis, data augmentation approaches unite these processes (Tanner & Wong, 1987). Two commonly used data augmentation approaches are maximum likelihood and Bayesian modeling. Maximum likelihood approaches work by maximizing a likelihood function built from the observed and missing data. Bayesian approaches work by sampling from a posterior distribution of missing data and model parameters. Next, we broadly clarify the intuition behind each approach and provide references for some of the more technical details.

The first data augmentation approach is maximum likelihood. Little and Rubin (2019) describe a likelihood function that incorporates the observed data, missing data, and model parameters of scientific interest. When the missing data mechanism is ignorable (i.e., the data are MCAR or MAR), this likelihood is marginalized (i.e., averaged, integrated) over all possible values of the missing items. This is useful because then the likelihood can be maximized with respect to only the observed data and model parameters. Dempster et al. (1977) proposed a novel method to evaluate this likelihood function: the EM algorithm. The EM algorithm consists of two steps: an expectation (E) step and a maximization (M) step. The algorithm begins with a set of initial values chosen for the model parameters. Then, the E step augments the observed data with plausible values of specific functions of the missing items created using the current model parameters. After the E step, the M step maximizes the likelihood that includes both the observed data and missing data to update parameter estimates. The E step is then repeated, followed by another M step, yielding new parameter estimates. This process continues until convergence, which means that the parameters are not meaningfully changing from iteration to iteration of the algorithm. The EM algorithm has seen widespread use in a variety of applications across many scientific disciplines (McLachlan & Krishnan, 2007).

The second data augmentation approach is Bayesian modeling (Gelman et al., 1995; Johnson et al., 2022; McElreath, 2018), which has seen widespread use in ecology (Cressie et al., 2009; Hobbs & Hooten, 2015; Hooten & Hobbs, 2015). Bayesian models differ from frequentist models by assuming that underlying model parameters are random variables and using a prior distribution to formally incorporate prior information into the model. Combining the prior information and the observed data yields the posterior distribution of the model parameters. Typically these posterior distributions are sampled directly using a Markov chain Monte Carlo (MCMC) algorithm like Metropolis–Hastings (Chib & Greenberg, 1995; Hastings, 1970; Metropolis et al., 1953; Tierney, 1994), Gibbs sampling (Casella & George, 1992; Gelfand & Smith, 1990; Geman & Geman, 1984), or Hamiltonian Monte Carlo (Carpenter et al., 2017; Duane et al., 1987). For a review of various MCMC approaches, see Robert et al. (1999). In a Bayesian model, missing data can be treated as model parameters, combined with the observed data and prior information to yield posterior distributions of each missing item. Samples from the various posterior distributions of each missing item provide valuable insights into their marginal and joint behaviors. These samples can be loosely viewed as “imputations” in that they “fill-in” plausible values of the missing data. They can be also used as part of a formal imputation method like Bayesian regression imputation.

### Inverse probability weighting and related approaches

Statistical survey designs leverage probability (i.e., random) sampling to estimate population parameters like means and totals; this randomization-based framework is called the design-based approach to statistics (Särndal et al., 2003). The design-based approach, which is based on probability sampling, is different from a model-based approach, which is based on assumptions of an underlying model (e.g., a linear regression model) generating the data (Brus & De Gruijter, 1997; Dumelle et al., 2022; Särndal et al., 1978). The design-based approach assigns to each unit in the sample a weight which quantifies how representative that unit is of the broader population of interest (i.e., target population). These weights generally equal the inverse of the probability the unit was selected in the sample and are called inverse probability weights (IPWs, Haziza & Beaumont, 2017; Seaman & Vansteelandt, 2018; Seaman & White, 2013). IPWs may then be combined with data and a design-based estimator (e.g., the Horvitz–Thompson estimator; see Horvitz & Thompson, 1952) to estimate population parameters; this approach is called inverse probability weighting.

Usually, the sum of the IPWs equals the population (more rigorously, the sampling frame) size. For example, consider a simple random sample (SRS) of size n from a known population of size N in which each unit is selected with probability $\frac{n}{N}$. Each IPW equals $\frac{N}{n}$, the sum of which (over the n units selected in the sample) equals

$$\sum_{i=1}^{n}\frac{N}{n}=\frac{nN}{n}=N,$$

the known population size. Rescaling over multiple variables is often achieved using approaches like poststratification, raking, and calibration (Lumley, 2011). Model-assisted estimators leverage models to make design-based estimation more efficient. A particularly useful model-assisted estimator is the generalized regression (GREG) estimator, which builds upon a linear regression framework (Cassel et al., 1976; Särndal et al., 1989). McConville et al. (2020) provide a review of model-assisted estimation with an application to forestry data.

Units selected in a sample may not respond or be available for data collection, which leads to missing data. Fortunately, IPWs can be adjusted, or rescaled, to account for nonresponse (Little, 1988a; Seaman & White, 2014). Often the adjusted weights are rescaled to sum to the original population size. One approach is to multiply each weight by the inverse of the response probability (Kott, 2012). For example, let the SRS IPW for each sampled unit equal $\frac{N}{np}$, where p (the probability that unit i is sampled) is the ratio $p=\frac{n^{*}}{n}$, where $n^{*}$ is the number of sampled units (out of a possible n units). The sum of the adjusted weights for the $n^{*}$ sampled units is

$$\sum_{i=1}^{n^{*}}\frac{N}{np}=\frac{n^{*}N}{np}=\frac{npN}{np}=N,$$

the known population size. Another approach is to adjust weights within categories (i.e., cells) using a similar rescaling approach based on population totals within each category (Heeringa et al., 2017). When units are partially sampled (i.e., some items are sampled), weights can be adjusted separately for each variable based on its nonresponse rate.

Instead of applying nonresponse adjustments to IPWs, imputation may be performed and then IPWs used on the complete data (Seaman et al., 2012). Little (1986) studies certain links between IPWs and imputation, while Hartman and Huang (2024) and Reiter et al. (2006) highlight the importance of including variables that influence sampling probabilities (i.e., survey design variables) in an imputation method. There is debate about whether weighted data should be used in an imputation method when performing design-based analysis on the complete data (Gelman, 2007; Lohr, 2021).

Carpenter and Smuk (2021) acknowledge some drawbacks of IPWs, one being that weight adjustments and subsequent analyses are sensitive to large weights. Little et al. (2024) note that inverse probability weighting is often inefficient compared to other methods that more naturally incorporate auxiliary information (e.g., imputation). Assigning IPWs is generally more straightforward, however, than imputation and data augmentation approaches.

Closely related to inverse probability weighting are techniques like superpopulation modeling quasirandomization, doubly robust estimation, and subsampling. Strictly speaking, inverse probability weighting is only applicable to probability samples that rely on design-based inference for estimation. The other methods are applicable to nonprobability samples and work by emulating certain properties of probability sampling, helping to ensure that the analysis is representative of the broader population of interest (Boyd et al., 2023). Superpopulation modeling builds a model (e.g., a linear regression model) that describes a response variable as a function of explanatory variables and then uses the model to predict unsampled units (Valliant et al., 2000). These predictions are combined with the observed data to estimate (realized) population parameters. Mc Overton et al. (1993) and Ver Hoef (2008) provide some applications of superpopulation modeling in ecology. In the quasirandomization approach, inclusion probabilities are modeled and then used in conjunction with other estimation routines (e.g., Horvitz–Thompson) that rely on weights (Elliott & Valliant, 2017). One example is propensity score approaches (Austin, 2011; Guo & Fraser, 2014; Rosenbaum & Rubin, 1983; Rubin, 2001), which aim to match units that have similar items for several variables (i.e., covariates) to control for confounding factors while studying the effect of a treatment. Doubly robust estimation combines elements of the superpopulation model and quasirandomization procedure and offers protection against certain types of model misspecification (Cao et al., 2009; Kang & Schafer, 2007; Robins et al., 1994; Wu, 2022). Finally, subsampling aims to create a subset of a sample that is a “miniature” of the population. A sample is a miniature of the population if the distribution of units in the sample is proportional to the distribution of units in the population with respect to some characteristic(s) (Kruskal & Mosteller, 1979). For example, if there are 60% woody wetlands and 40% herbaceous wetlands in a population, a sample is a miniature if it contains 60% woody wetlands and 40% herbaceous wetlands. Beck et al. (2014) and Steen et al. (2020) provide some applications of subsampling in ecology. Boyd et al. (2024) provide a review of superpopulation modeling, quasirandomization, doubly robust estimation, and subsampling, as well as several other extensions, and apply each method to biodiversity data. Like inverse probability weighting, superpopulation modeling, quasirandomization, and doubly robust estimation have extensions to accommodate missing data. For superpopulation modeling examples, see Boyd et al. (2025); for quasirandomization (more specifically, propensity score matching) examples, see Cham and West (2016), Choi et al. (2019), D’Agostino and Rubin (2000), and Little and Vartivarian (2005); for doubly robust estimation examples, see Kim and Haziza (2014) and Robins (2000); and for a subsampling example, see Meng (2022).

### Specific approaches for MNAR data

Due to the unique character of MNAR data, namely that missingness depends on the values of hidden items, methods for MNAR missingness typically require making untestable assumptions about the relationship between the missing values and the probability of missingness (unless the quantity is recoverable under MNAR missingness and the DAG is specified correctly, as in Mohan & Pearl, 2021). Thus, while there are classes of methods for handling MNAR data, specific solutions require an understanding of the dataset in question and relevant assumptions, as well whether these assumptions apply parametric modeling (Little & Rubin, 2019) or conditional independence (Mohan & Pearl, 2021). The definition of MNAR missingness in Equation (2) implies that the missingness is nonignorable, which means that the distribution of R must be modeled jointly with Y:Pr(R=r,Y=y). This may be constructed in several different ways, though the two most prominent model classes are selection models and pattern mixture models. Selection models decompose the joint distribution into the product of the conditional distribution of the missingness mechanism given the data values and the distribution of the data values: Pr(R=r∣Y=y)Pr(Y=y). Pattern mixture models decompose the joint distribution into the product of the data values given the missingness mechanism and the distribution of the missingness mechanism: Pr(R=r)Pr(Y=y∣R=r). Pattern mixture models and selection models directly decompose the joint distribution, but an alternative approach, called shared parameter models, supposes that there is an unobservable variable, U, that makes the distribution factorize: Pr(R=r∣U=u)Pr(Y=y∣U=u). We will not explore shared parameter models in this manuscript, but they are discussed in Molenberghs et al. (2014). Little and Rubin (2019) recommend against using selection models because the assumptions behind these approaches can obfuscate how information on fully observed cases informs the distributions for the partially observed cases. They argue pattern mixture models make more explicit the connection between how the data and missingness mechanisms interact. Bailey (2024) provides a general and thorough review of methods for handling MNAR data.

To help make these MNAR concepts more concrete, consider our running example of wetland data with variables VT, SH, and VMMI, and missing items in each variable (see Missing data in more than one variable). In this example, we implicitly assumed that the units (or rows of $y_{\text{obs}}$) are independent of one another. We can then summarize the distribution of $R_{i}$, or the rows in the matrix R:

$$r=\left[\begin{array}{cccc} \text{Unit} & \text{VT} & \text{SH} & \text{VMMI}\\ 1 & 1 & 1 & 1\\ 2 & 0 & 1 & 0\\ 3 & 1 & 1 & 1\\ 4 & 1 & 1 & 1\\ 5 & 1 & 0 & 1\\ 6 & 1 & 1 & 0 \end{array}\right].$$

In this case, $r_{i}$ takes the values {1, 1, 1} for $i=1,3,4,r_{2}=\{0,1,0\}$, $r_{5}=\{1,0,1\}$, and $r_{6}=\{1,1,0\}$. The conditional distribution of $y_{i}|r_{i}$ in the pattern mixture model comprises four distributions, namely the distribution $y_{i}=\left({\text{VT}}_{i},{\text{SH}}_{i},{\text{VMMI}}_{i}\right)$ for each of the four values for $r_{i}$. The distribution $\text{Pr}\left(y_{i}|r_{i}=\{1,1,1\}\right)$ is completely informed by the observed items (i.e., there are no missing items). However, when considering $\text{Pr}\left(y_{i}|r_{i}=\{1,0,1\}\right)$ for instance, there is no information in the data to learn the distribution for ${\text{SH}}_{i}$, though we do have information about ${\text{VT}}_{i}$ and ${\text{VMMI}}_{i}$. If the missing data mechanism is assumed MAR, this implies that $\text{Pr}\left(y_{i}|r_{i}=\{1,0,1\}\right)=\text{Pr}\left(y_{i}|r_{i}=\{1,1,1\}\right)$, leading to the following distributional substitutions:

$$\text{Pr}\left({\text{SH}}_{i}|{\text{VT}}_{i}={\text{vt}}_{i},{\text{VMMI}}_{i}={\text{vmmi}}_{i},r_{i}=\{1,0,1\}\right)=\;\text{Pr}\left({\text{SH}}_{i}|{\text{VT}}_{i}={\text{vt}}_{i},{\text{VMMI}}_{i}={\text{vmmi}}_{i},r_{i}=\{1,1,1\}\right).$$

If the missing data mechanism is instead assumed MNAR, the data lack information from which to learn each distribution, which is one reason why MNAR data pose unique challenges.

One solution for handling MNAR data is a sensitivity analysis applied to the pattern mixture model. For example, one could make the following assumption:

$$[\text{SH}|\text{VT},\text{VMMI},r=\{1,1,1\}]\sim \text{Gaussian}\left(\mu_{1},\sigma_{1}^{2}\right)$$

$$\mu_{1}\equiv \beta_{0}+\beta_{1}ℐ(\text{VT})+\beta_{2}\text{VMMI}.$$

and then further assume that

$$[\text{SH}|\text{VT},\text{VMMI},r=\{1,0,1\}]\sim \text{Gaussian}\left(\mu_{2},\sigma_{2}^{2}\right)$$

$$\mu_{2}\equiv \mu_{1}+\delta .$$

A range of plausible values for δ and their analysis implications can be studied. The analysis is repeated with each value of δ and the range of outcomes is reported. Sensitivity analysis applied to pattern mixture models has many extensions like proxy-pattern mixture models, where proxies for the missing items are introduced (Andridge & Little, 2011, 2020; Little, 1994).

Despite Little and Rubin’s (2019) contention that pattern mixture models are more interpretable, selection models have found wide use in fields such as economics and public health. The canonical selection model is the Heckman model, introduced in Heckman (1976, 1979), which introduces dependence between the errors of the outcome equation and the missingness equation through a joint normal distribution. For the sake of simplicity, assume that we are modeling only VMMI and its missingness, denoted as ${\text{R}}_{\text{VMMI}}$, given VT and SH:

(6) $${\text{VMMI}}_{i}=\beta_{0}+\beta_{1}ℐ\left({\text{VT}}_{i}\right)+\beta_{2}{\text{SH}}_{i}+\eta_{i}$$

(7) $${\left(R_{\text{VMMI}}\right)}_{i}=\left\{\begin{array}{ll} 1 & \gamma_{0}+\gamma_{1}ℐ\left({\text{VT}}_{i}\right)+\gamma_{2}{\text{SH}}_{i}+\epsilon_{i}\geq 0\\ 0 & \gamma_{0}+\gamma_{1}ℐ\left({\text{VT}}_{i}\right)+\gamma_{2}{\text{SH}}_{i}+\epsilon_{i}<0 \end{array}\right.$$

(8) $$\left(\eta_{i},\epsilon_{i}\right)\overset{\text{iid}}{\sim }\text{MultivariateGaussian}\left((0,0),\left[\begin{array}{cc} \sigma_{\text{VMMI}}^{2} & \rho \sigma_{\text{VMMI}}\\ \rho \sigma_{\text{VMMI}} & 1 \end{array}\right]\right)$$

The probability model agrees with the DAG presented in Figure 6, though the DAG does not encode the assumptions related to the multivariate Gaussian errors or the linear equations. The variable U in the DAG (Figure 6) represents an observed common cause of VMMI and $R_{\text{VMMI}}$, which implies the correlation between $\eta_{i}$ and $\epsilon_{i}$ in Equation (8).

When the covariates in Equation (6) match those of Equation (7), the joint normality assumption is necessary to make the coefficients estimable in large samples without bias (i.e., recoverable). When there is a variable included in Equation (7) that is not included in Equation (6), one may relax the joint normality assumption (Chamberlain, 1986; Chib et al., 2009; Heckman et al., 2006; Newey et al., 1990).

It is important to note that the Heckman model and pattern mixture models can be seen as data augmentation approaches to missing data analysis. For example, the Heckman model may be fit using data augmentation through a fully Bayesian routine that includes latent, bivariate Gaussian errors for each unit with missing data. This demonstrates that data augmentation is not limited to only MAR problems. Multiple imputation can be used for MNAR problems as well, some of which employ modifications similar to the sensitivity parameter approach for the pattern mixture models discussed above (Van Buuren, 2018) and others that include the Heckman model as part of the imputation procedure (Galimard et al., 2016).

Mohan and Pearl (2021) include several examples of DAGs for MNAR problems that admit quantities that are recoverable without assumptions like normality or linearity. In these cases, simple ratio estimators are used in lieu of data augmentation or multiple imputation and it can be shown that these techniques will recover the correct quantity in large samples. Whether or not these estimators’ bias and variance are acceptable in small-to-moderate-sized samples, however, will depend on the data at hand.

An entirely different perspective is to add variables to the imputation method that are related to the missing items (and their mechanisms). The hope is that including these additional variables subsequently satisfies the MAR assumption. Even when the inclusion of these variables fails to strictly satisfy MAR, they can help mitigate the impact of failing to capture the specific MNAR mechanism of the data (Schafer & Graham, 2002). This practice does increase the risk of introducing collider bias (Thoemmes & Rose, 2014), however, so it should be carried out thoughtfully.

### Contingency variables

We clarified the difference between meaningful and nonmeaningful missingness in Meaningful and non-meaningful missingness. Briefly, a missing item is meaningful if a measurement does exist but its value is hidden. A missing item is nonmeaningful if no measurement can exist. For example, if there is no surface water at a wetland, then water chemistry variables (that require surface water) cannot be measured.

We call a variable a contingency variable if it is an indicator (i.e., binary) variable that partitions the data into two groups based on whether missing data are measurable (i.e., able to be measured) or not. If an item is measurable but missing, the missingness is meaningful and standard missing data theory may be applied to these items. If an item is not measurable, the missingness is nonmeaningful and standard missing data theory may not be applied to these items. Contingency variables are closely related to the idea of follow-up questions in surveys (Eckman et al., 2014), whereby participants are asked separate sets of questions based on their responses to previous answers. For example, “How well do you get along with your siblings?” is only a relevant question if the responder previously indicated they had siblings (Schafer & Graham, 2002).

Contingency variables may be of direct interest themselves or used to condition upon meaningful missingness (e.g., subset the data so that they only contain items that are measurable). For example, a researcher may estimate the proportion of wetlands with and without surface water, in which the contingency variable is of direct interest. Alternatively, a researcher may estimate the average nitrogen concentration in wetlands with surface water after subsetting the data so that they only contain wetlands with surface water. We explore several applications of contingency variables to 2016 NWCA data in Appendix S4: Section S1.

### Other missing data approaches

We briefly describe here several other missing data approaches from the literature. Van Buuren (2018) provides an overview of imputation methods specific to certain data types like binary, count, categorical, semicontinuous, hierarchical, longitudinal, multivariate, and censored data. Helsel (2011) discusses imputation in the context of censored ecological data. Deng et al. (2016) and Zhao and Long (2016) provide a bootstrap Lasso imputation procedure for high-dimensional data, Box and Tiao (2011) and Rubin (2004) show how a Bayesian linear regression model can be used to generate imputations, and Shah et al. (2014) study the utility of a random forest model to generate imputations. Another missing data problem is that of imperfect detection, whereby the presence or absence of a species cannot be detected with certainty (Conn et al., 2012; Kellner & Swihart, 2014; MacKenzie, 2005). Higham et al. (2021) study imperfect detection in the context of superpopulation modeling of ecological data. Finally, the missing data indicator method aims to control for the effect of missing data by building directly into a regression model (1) an indicator variable that represents missingness and (2) a variable that is the interaction between the indicator variable in (1) and another variable. This method has mixed results—some highlight its utility (Groenwold et al., 2012; Sullivan et al., 2018; White & Thompson, 2005), while some highlight its drawbacks (Donders et al., 2006; Groenwold et al., 2012; Jones, 1996; Knol et al., 2010; Van der Heijden et al., 2006).

## CASE STUDIES

### A comparison of various missing data approaches for MAR missing data

Here, we compare the performance of 13 unique missing data approaches for MAR missing data: CCA, three deterministic imputation methods, four single imputation methods, four multiple imputation methods, and a data augmentation approach (Table 3). The three deterministic imputation methods are mean imputation, regression imputation, and nearest neighbor imputation. The four single imputation methods are stochastic regression imputation, predictive mean matching (Type-0 matching, three donors), bootstrap regression imputation, and predictive mean matching (Type-1 matching, three donors). Recall that stochastic regression imputation and predictive mean matching (Type-0 matching) incorporate randomness in the imputations but not the underlying imputation method parameters, while bootstrap regression imputation and predictive mean matching (Type-1 matching) incorporate randomness in both the imputations and underlying imputation method parameters. The four multiple imputation methods are the multiple imputation version of aforementioned single imputation methods. The data augmentation method is a fully Bayesian model. Henceforth, we refer to each method using the abbreviations from Table 3.

We measured the inferential performance of each of the 13 missing data approaches by tracking various metrics across 2000 simulation trials. Each trial was conducted independent of other trials. Within each trial, 100 observations were independently generated from the model:

(9) $${\text{y}}_{i}=\beta_{0}+\beta_{1}ℐ\left({\text{x}}_{1,i}\right)+\beta_{2}{\text{x}}_{2,i}+\epsilon_{i},$$

where ${\text{y}}_{i}$ is the ith value of a general response variable, ${\text{x}}_{1,i}$ is the ith value of a general categorical variable that belongs to one of two equally-likely levels (“A” and “B”), $ℐ\left({\text{x}}_{1,i}\right)$ is an indicator variable defined as

$$ℐ\left({\text{x}}_{1,i}\right)=\left\{\begin{array}{ll} 1 & \text{if}\;{\text{x}}_{1,i}=\text{B}\\ 0 & \text{if}\;{\text{x}}_{1,i}=\text{A} \end{array},\right.$$

${\text{x}}_{2,i}$ is the ith value of a general continuous variable from a Gaussian distribution with mean zero and variance one, $\epsilon_{i}$ is the ith random error from a Gaussian distribution with mean zero and variance one, $\beta_{0}$ is an intercept parameter, and $\beta_{1},\beta_{2}$ are slope parameters that control the impact of $ℐ\left({\text{x}}_{1,i}\right)$ and ${\text{x}}_{2,i}$ on ${\text{y}}_{i}$, respectively. In each simulation trial, the true values of $\beta_{0},\beta_{1}$, and $\beta_{2}$ were fixed at one, implying Equation (9) can be synonymously rewritten as

$${\text{y}}_{i}=1+ℐ\left({\text{x}}_{1,i}\right)+{\text{x}}_{2,i}+\epsilon_{i}.$$

In each trial, we randomly assigned the 100 simulated observations simulated to distinct training and test sets (Gareth et al., 2013). The training set contained 99 observations and was used to estimate the β parameters of a linear regression model with the same form as in Equation (9). The test set contained one observation which was later used to evaluate the fitted linear regression model’s predictive capacity.

After simulating the training data, each item in y and $x_{2}$ was randomly and independently assigned to be observed or missing. The probability that each item was missing depended entirely on the observed value of $x_{1}$:

$$\text{Pr}\left({\text{R}}_{{\text{x}}_{2},i}=1|{\text{x}}_{1,i}=\text{A}\right)=0.7$$

$$\text{Pr}\left({\text{R}}_{{\text{x}}_{2},i}=1|{\text{x}}_{1,i}=\text{B}\right)=0.35$$

$$\text{Pr}\left({\text{R}}_{\text{y},i}=1|{\text{x}}_{1,i}=\text{A}\right)=0.35,\text{and}$$

$$\text{Pr}\left({\text{R}}_{\text{y},i}=1|{\text{x}}_{1,i}=\text{B}\right)=0.7.$$

for all i (units) in the complete data. The individual missingness probabilities themselves are not important—what is important is that $x_{1}$, when conditioned upon, completely characterizes the missingness mechanism in $x_{2}$ and y, and hence the data are MAR (given $x_{1}$). For CCA and the imputation methods, the β parameters were estimated using ordinary least squares (or synonymously, maximum likelihood). The imputation methods used y and $x_{1}$ to impute missing items in $x_{2}$ and $x_{1}$ and $x_{2}$ to impute missing items in y. For FBDA, default priors were assumed for the β parameters and each missing item (the prior distribution for missing $x_{2}$ items was an intercept-only model). We used the mice package (Van Buuren & Groothuis-Oudshoorn, 2011) for the imputation methods and the brms package (Bürkner, 2017) for FBDA. Both packages are part of the R programming language (R Core Team, 2025).

Inferential performance for the 13 missing data approaches was measured using three metrics: mean bias, root-mean-squared error (RMSE), mean SE, and mean 95% CI coverage. These metrics were calculated for both $\beta_{1}$ and $\beta_{2}$ slope parameters (we ignored the intercept, $\beta_{0}$, as this tends to be of little scientific interest). Mean bias (MBias) is the average deviation of $\hat{\beta }$ (an estimate) from β (the true value) across trials:

$$\text{MBias}(\hat{\beta })=\frac{1}{2000}\sum_{i=1}^{2000}\left({\hat{\beta }}_{i}-\beta \right)$$

RMSE is the square root of the average squared deviation of $\hat{\beta }$ from β across trials.


$$\text{RMSE}(\hat{\beta })=\sqrt{\frac{1}{2000}\sum_{i=1}^{2000}{\left({\hat{\beta }}_{i}-\beta \right)}^{2}}$$

The mean SE is the average SE of $\hat{\beta }$. Mean 95% CI coverage (Cover95) is the proportion of 95% Gaussian CIs that contain β:

$$\text{Cover}95(\hat{\beta })=\frac{1}{2000}\sum_{i=1}^{2000}ℐ\left({\text{Cover}}_{i}\right)$$

$$ℐ\left({\text{Cover}}_{i}\right)=\left\{\begin{array}{ll} 1 & \text{if}\;95\text{LB}\left({\hat{\beta }}_{i}\right)\leq \beta \leq 95\text{UB}\left({\hat{\beta }}_{i}\right)\\ 0 & \text{Otherwise} \end{array},\right.$$

where $95\text{LB}\left({\hat{\beta }}_{i}\right)\;\left(95\text{UB}\left({\hat{\beta }}_{i}\right)\right)$ is the 95% CI lower (upper) bound for β based on ${\hat{\beta }}_{i}$. For FBDA, $95\text{LB}\left({\hat{\beta }}_{i}\right)$ and $95\text{UB}\left({\hat{\beta }}_{i}\right)$ are actually bounds of 95% credible intervals (using quantiles 0.025 and 0.975), but we call them CIs for consistency with the other methods. We expect the 95% credible intervals to exhibit frequentist coverage properties given that weakly informative default priors were used, which implies inference should be primarily driven by the likelihood (Bürkner, 2017; Gelman et al., 1995; Jaynes & Kempthorne, 1976); it is also often desirable for Bayesian intervals to have nominal Frequentist coverage, and is thus a common way of evaluating Bayesian intervals (Little, 2006). Well-fitting models should have mean bias (MBias) close to zero (i.e., parameter estimates are correct on average) and “proper” coverage (Cover95). We define proper coverage as being between 0.94 and 0.96 (after rounding to two digits), a range of plausible empirical coverages given natural simulation variability. For unbiased models with proper coverage, lower RMSE is preferred, as this indicates more efficient (i.e., precise) estimation of β.

Table 4 provides MBias, RMSE, and Cover95 for the missing data approaches. All 13 methods except mean imputation had little to no MBias (relative to RMSE). Deterministic imputation methods (Mean, Reg, NN) performed worse (higher RMSE; lower Cover95) than single imputation methods (StReg-S, PMMT0-S, Boot-S, PMMT1-S). Single imputation methods performed worse (higher RMSE; lower Cover95) than their multiple imputation counterparts (StReg-M, PMMT0-M, Boot-M, PMMT1-M). The only methods with proper Cover95 were CCA, Boot-M, PMMT1-M, and FBDA. Among these, Boot-M, PMMT1-M, and FBDA had better (lower) RMSE than CCA (and hence were more efficient). There was little difference in performance among Boot-M, PMMT1-M, and FBDA.

We also evaluated predictive performance of the 13 missing data approaches. We used each fitted linear regression model to predict ${\text{y}}_{\text{test}}$, the true value of y from the test data. Predictions were derived for CCA, the deterministic imputation methods (Mean, Reg, NN), and the single imputation methods (StReg-S, PMMT0-S, Boot-S, PMMT1-S) using the single fitted model:

$$\text{Pred}\left({\text{y}}_{\text{test}}\right)={\tilde{\text{y}}}_{\text{test}}=\hat{\beta_{0}}+\hat{\beta_{1}}ℐ\left({\text{x}}_{1,\text{test}}\right)+\hat{\beta_{2}}{\text{x}}_{2,\text{test}},$$

where ${\text{x}}_{1,\text{test}}$ and ${\text{x}}_{2,\text{test}}$ are the ${\text{x}}_{1}$ and ${\text{x}}_{2}$ values for the test unit and the $\hat{\beta }$ are estimated parameters from the respective linear regression model. We derive the variance of $\text{Pred}{(\text{y}}_{\text{test}})$ in Appendix S5: Section S1. For the multiple imputation methods (StReg-M, PMMT0-M, Boot-M, PMMT1-M), predictions of ${\text{y}}_{\text{test}}$ were made separately for each complete dataset and then pooled using Rubin’s Rules to create a final prediction (i.e., PC).

Predictive performance metrics were mean prediction bias, root-mean-squared-prediction error (RMSPE), mean prediction SE, and mean 95% prediction interval coverage of ${\text{y}}_{\text{test}}$. Mean prediction bias (MPBias) is the average deviation of ${\tilde{\text{y}}}_{\text{test}}$ (a prediction) from ${\text{y}}_{\text{test}}$ (the true value) across trials:

$$\text{MPBias}\left({\tilde{\text{y}}}_{\text{test}}\right)=\frac{1}{2000}\sum_{i=1}^{2000}\left({\tilde{\text{y}}}_{\text{test},i}-{\text{y}}_{\text{test},i}\right).$$

RMSPE is the square root of the average squared deviation ${\tilde{\text{y}}}_{\text{test}}$ from ${\text{y}}_{\text{test}}$ across trials:

$$\text{RMSPE}(\tilde{\text{y}})=\sqrt{\frac{1}{2000}\sum_{i=1}^{2000}{\left({\tilde{\text{y}}}_{\text{test},i}-{\text{y}}_{\text{test},i}\right)}^{2}}.$$

The mean prediction SE is the average SE of $\tilde{\text{y}}$. Mean 95% prediction interval coverage (PCover95) is the proportion of 95% Gaussian prediction intervals that contain ${\text{y}}_{\text{test}}$:

$$\text{PCover}95\left({\tilde{\text{y}}}_{\text{test}}\right)=\frac{1}{2000}\sum_{i=1}^{2000}ℐ\left({\text{PCover}}_{i}\right)$$

$$ℐ\left({\text{PCover}}_{i}\right)=\left\{\begin{array}{ll} 1 & 95\text{LB}\left({\tilde{\text{y}}}_{\text{test},i}\right)\leq {\text{y}}_{\text{test},i}\leq 95\text{UB}\left({\tilde{\text{y}}}_{\text{test},i}\right)\\ 0 & \text{Otherwise} \end{array}\right..$$

As with the CIs from the inferential comparison, the FBDA prediction intervals are actually credible intervals, but we call them prediction intervals for consistency with other methods. We call prediction interval coverage proper when it is between 0.94 and 0.96.

Table 5 provides MPBias, RMSPE, and PCover95 for all 13 missing data approaches. All methods except mean imputation had little to no MPBias (relative to RMSPE). Deterministic imputation methods (Mean, Reg, NN) performed worse (higher RMSPE; lower PCover95) than the single imputation methods (StReg-S, PMMT0-S, Boot-S, PMMT1-S), although NN did have proper coverage. Single imputation methods performed worse (higher RMSPE; lower PCover95) than their multiple imputation counterparts (StReg-M, PMMT0-M, Boot-M, PMMT1-M). The multiple imputation methods and FBDA tended to have the best (lowest) RMSPE, followed closely by CCA and the single imputation methods. Overall, this simulation study suggests that the performance gap between the worst and best missing data approaches may be much narrower for prediction (Table 5) than for inference (Table 4).

In Appendix S2: Section S1, we compared the performance of an additional six imputation methods—the single and multiple imputation versions of bootstrap lasso, Bayesian linear regression, and random forest. We found that bootstrap lasso and Bayesian linear regression performed similarly as the other single and multiple imputation methods. However, random forest imputation was inefficient and lacked proper coverage, even for its single imputation implementation. Also in Appendix S2: Section S1, we compare mean, regression, and bootstrap imputation via their capacity to recover missing items and show why this evaluation metric can be misleading when the statistical analysis goals are parameter estimation, prediction, and uncertainty quantification.

### The impact of including the response variable in an imputation method for MAR missing data

We studied the impact of including the response variable in an imputation method for MAR missing data using two approaches: regression imputation (a deterministic approach) and bootstrap multiple imputation (a stochastic approach). Data were simulated using the same setup as in the previous case study. That is, within each of 2000 simulation trials, 100 observations were independently generated from the model in Equation (9), test and training sets were chosen, and missingness indicators were simulated. Previously, the response variable was included in the imputation method for $x_{2}$. Here, we recreate that scenario and compare it to another scenario where the response variable, y, was not included in the imputation method for $x_{2}$. In total, we compared four approaches: regression imputation including y in the imputation method for $x_{2}$ (Reg-Yes), regression imputation omitting y from the imputation method for $x_{2}$ (Reg-No), bootstrap multiple imputation including y for $x_{2}$ (Reg-M-Yes), and bootstrap multiple imputation omitting y for $x_{2}$ (Reg-M-No).

Of the four approaches, Boot-M-Yes is the only one that is unbiased with proper interval coverage for both slope coefficients ($\beta_{1}$ and $\beta_{2}$) and predictions $\left({\text{y}}_{\text{new}}\right)$ (Tables 6 and 7). Boot-M-Yes was also the most efficient (lowest RMSE, RMSPE) among the four approaches. Boot-M-No was unbiased for $\beta_{1}$ and predictions, slightly overcovering both, and was biased for $\beta_{2}$ with notably low coverage. Reg-No was unbiased for both slope coefficients and predictions and was more efficient with better coverage than Reg-Yes. While Reg-No coverage was better than Reg-Yes, Reg-No coverage still was never proper, though coverage likely could be improved by incorporating a resampling approach (D’Agostino McGowan et al., 2024). Reg-Yes was unbiased for $\beta_{1}$ and predictions, biased for $\beta_{2}$, and never had proper coverage. Overall, these results replicate those found by D’Agostino McGowan et al. (2024): It is beneficial to include the response variable if the imputation method is stochastic (e.g., single or multiple imputation); it is harmful to include the response variable if the imputation method is deterministic (e.g., regression imputation).

### A comparison of various missing data approaches for MNAR missing data

Our simulation study for MNAR missingness mirrors the simulation study for MAR missingness with one key difference: We introduce dependence between ${\text{r}}_{i}$ and ${\text{y}}_{i}$, as can be seen below in Equation (10). Recall the data-generating process for the MAR simulation study from above. Each outcome unit i was associated with two predictors, one binary prediction, ${\text{x}}_{1,i}$, and one continuous, ${\text{x}}_{2,i}$. Given these two predictors, and independent and identically distributed (i.i.d.) Gaussian error terms (with mean zero and variance one) $\eta_{i}$, we generated ${\text{y}}_{i}$ according to the following equation:

$${\text{y}}_{i}=\beta_{0}+\beta_{1}ℐ\left({\text{x}}_{1,i}\right)+\beta_{2}{\text{x}}_{2,i}+\eta_{i}.$$

Given ${\text{y}}_{i},\rho$, a parameter that controls the extent of $r_{i}$’s dependence on ${\text{y}}_{i}$, and a second i.i.d. Gaussian error term (with mean zero and variance 1-ρ) $\varepsilon_{i}$, independent of $\eta_{i}$, we then generated ${\text{r}}_{i}$, the binary observation indicator for ${\text{y}}_{i}$, from the following equation:

(10) $${\text{r}}_{i}|\left.{\text{y}}_{i}=\left\{\begin{array}{ll} 1 & \theta_{0}+\theta_{1}ℐ\left({\text{x}}_{1,i}\right)+\theta_{2}{\text{x}}_{2,i}+\rho {\text{y}}_{i}+\varepsilon_{i}\geq 0\\ 0 & \theta_{0}+\theta_{1}ℐ\left({\text{x}}_{1,i}\right)+\theta_{2}{\text{x}}_{2,i}+\rho {\text{y}}_{i}+\varepsilon_{i}<0 \end{array}\right.\right.$$

The correlation parameter ρ can take values between (−1, 1). If ρ=0, the problem is MAR. If ρ≠0, the missingness is MNAR. The magnitude of ρ controls the strength of MNAR missingness. The model above is the Heckman model introduced in Specific approaches for MNAR data, but expressed in a slightly different manner. This formulation makes the dependence of ${\text{r}}_{i}$ on ${\text{y}}_{i}$ explicit, and suggests a fully Bayesian data augmentation procedure for model fitting and prediction.

We simulated data under three different scenarios reflecting increasing strength of MNAR missingness. The scenarios were small, medium, and large, corresponding to ρ of 0.1, 0.5, and 0.9, respectively. We then fitted two models: one correctly formulated model, which is the Heckman selection model, and one model that incorrectly assumed that missingness was MAR. We evaluated performance using MBias, RMSE, and Cover95 of the slope parameters $\beta_{1}$ and $\beta_{2}$ as well as MPBias, RMSPE, and PCover95 of ${\text{y}}_{\text{test}}$ in the test data.

From Table 8, both the MAR and Heckman (MNAR) models had non-negligible bias for $\beta_{1}$ and $\beta_{2}$, though the (absolute) MBias was always lower than the RMSE. The absolute MBias also decreased as the strength of MNAR missingness decreased. The Heckman model RMSE was always lower than the MAR model RMSE, and the magnitude of this difference decreased as the strength of MNAR missingness decreased. When the MNAR missingness was large or moderate, the MAR Cover95 was noticeably low (between 0.25 and 0.89), while the Heckman model Cover95 was much more appropriate (between 0.91 and 0.98). When the MNAR missingness was small, the MAR model had proper Cover95 while the Heckman model slightly overcovered.

From Table 9, both the MAR and Heckman (MNAR) models had non-negligible MPBias for predictions when the strength of MNAR missingness was large or moderate, though the (absolute) MPBias was much lower than the RMSPE. The magnitude of the MPBias decreased as the strength of MNAR missingness decreased, and when the strength of MNAR missingness was small, so too was the absolute MPBias. The Heckman model RMSPE was always lower than the MAR model RMSPE, and the magnitude of this difference decreased as the strength of MNAR missingness decreased. When the MNAR missingness was large, the MAR coverage was noticeably low (0.79) while the Heckman PCover95 was much more appropriate (0.93). When the MNAR missingness was moderate or small, both the Heckman and MAR models had coverage that was either proper or close to proper (between 0.91 and 0.97).

In Appendix S3: Section S1, we also studied the performance of two pattern mixture models, one assuming a moderate amount of MNAR missingness, and another assuming a large amount of MNAR missingness. We found that the pattern mixture models had similar MBias (MPBias), RMSE (RMSPE), and Cover95 (PCover95) for fixed effects (predictions) as the MAR models but much more appropriate coverage (i.e., much closer to 0.95). When the strength of MNAR missingness was moderate, the moderate pattern mixture model outperformed the large pattern mixture model, while the opposite was true when the MNAR amount was large, as expected.

Broadly, these results show that when the strength of MNAR missingness is large or moderate, MNAR models can outperform MAR models, sometimes noticeably so. In our study, while there was some bias for the MNAR models, theoretically this bias should decrease to zero as long as the model is correctly specified and the sample size increases. When the strength of MNAR missingness is small, both the MNAR and MAR models can produce similar results.

### The effect of missing data on a spatial regression model

Many datasets in ecology are spatially structured. Spatially structured data pair site-specific information with their geographic location. Statistical models can formally leverage the spatial structure in these types of data by characterizing its spatial covariance (i.e., correlation, dependence), which can notably improve model performance (Cressie, 1993). However, the utility of missing data approaches for spatially structured data is not well studied, so next we provide an introductory look at this problem using spatial linear regression models. Before doing so, we describe some statistical modeling terminology.

A typical linear regression model can be written as

(11) $${\text{y}}_{i}=\beta_{0}+\beta_{1}{\text{x}}_{i,1}+\beta_{2}{\text{x}}_{i,2}+\cdots +\beta_{p}{\text{x}}_{p,i}+\epsilon_{i},$$

where ${\text{y}}_{i}$ is the response variable measured at site $i,{\text{x}}_{i,j}$ is the jth explanatory variable measured at site i, and $\epsilon_{i}$ is the independent random error at site i. Typically, $\epsilon_{i}$ is assumed to be a Gaussian (i.e., normal) random variable with mean zero and variance $\sigma_{\epsilon }^{2}$. Moreover, elements of ϵ at two distinct sites are assumed independent of one another. That is, no information from site i is borrowed by site k through $\epsilon_{i}$ and $\epsilon_{k}$. We call the model in Equation (11) the “nonspatial linear model” because of the independence among the errors.

A spatial linear regression model can be written as

(12) $${\text{y}}_{i}=\beta_{0}+\beta_{1}{\text{x}}_{i,1}+\beta_{2}{\text{x}}_{i,2}+\cdots +\beta_{p}{\text{x}}_{p,i}+\tau_{i}+\epsilon_{i},$$

where ${\text{y}}_{i},{\text{x}}_{i,j}$, and $\epsilon_{i}$ are defined the same as in Equation (11). The difference between the model in Equation (11) and the model in Equation (12) is the inclusion of $\tau_{i}$, which is the spatially dependent random error at site i. Typically, $\tau_{i}$ is assumed to be a Gaussian random variable with mean zero and variance $\sigma_{\tau }^{2}$. However, elements of τ at two distinct sites are spatially dependent, and the strength of dependence depends on the proximity of the sites. Put simply, information from site i is borrowed by site k through $\tau_{i}$ and $\tau_{k}$. We call the model in Equation (11) the “spatial linear model” because of the spatial dependence among the errors.

The spatial linear model has two sources of variability whose magnitudes are captured by $\sigma_{\tau }^{2}$ and $\sigma_{\epsilon }^{2}$. The term $\sigma_{\tau }^{2}$ captures coarse-scale variability that is spatially structured while the term $\sigma_{\epsilon }^{2}$ captures fine-scale variability (e.g., from measurement error or microscale variation) that is not spatially structured. In spatial statistics, $\sigma_{\tau }^{2}$ and $\sigma_{\epsilon }^{2}$ are called the partial sill and nugget, respectively. Spatial dependence is formally measured using a covariance function. One example of a spatial covariance function is the exponential function, defined as

$$\text{Cov}\left({\text{y}}_{i},{\text{y}}_{k}\right)=\left\{\begin{array}{ll} \sigma_{\tau }^{2}\text{exp}\left(-{\text{h}}_{ik}/\phi \right) & i\neq j\\ \sigma_{\tau }^{2}+\sigma_{\epsilon }^{2} & i=j \end{array},\right.$$

where ${\text{h}}_{ik}$ is the Euclidean (i.e., straight-line) distance between site i and site k. The term ϕ is called a range parameter, and it controls the distance-decay rate of the spatial covariance. As the distance between site i and site k increases, the spatial covariance between the sites decreases, which reflects the intuition that sites close together in geographic space tend to be more similar than sites far apart in geographic space (Tobler, 1970). Zimmerman and Ver Hoef (2024) provide a thorough introduction to spatial linear models for ecological and environmental data, outlining parameter estimation, hypothesis testing, prediction (i.e., Kriging) at new units (i.e., sites), and more.

Suppose that a researcher’s goal is to study the effect of soil hardening and vegetation type on VMMI using a spatial linear model. The structure of that spatial linear model is

(13) $${\text{VMMI}}_{i}=\beta_{0}+\beta_{1}ℐ\left({\text{VT}}_{i}\right)+\beta_{2}{\text{SH}}_{i}+\tau_{i}+\epsilon_{i}.$$

Using 2016 NWCA VMMI data (Figure 7), we fit spatial linear models having the form in Equation (13), with the intent to quantify the impact of missing data on the spatial linear model. First, 641 (two-thirds) of the 962 fully observed sites were placed into a training dataset used to fit models and the remaining 321 (one-third) sites were placed into a test dataset used to evaluate predictive performance. Then, a spatial linear model of the form in Equation (13) was fit to the training data and used to predict VMMI in the test data. We call this model the “SP-ALL” model because no training data were missing (i.e., the model was fit using all the data). Next, we simulated MAR missing data in the SH variable based on the VT variable. When VT was woody, each SH score was randomly assigned a missingness indicator with probability 0.3. When VT was herbaceous, each SH score was randomly assigned a missingness indicator with probability 0.6. Because the missingness in VT is explained via SH, the missing data are MAR (given SH). We used bootstrap multiple imputation (with 100 imputed datasets) to impute missing SH items in the training data and fit spatial linear models to each complete dataset, appropriately pooling parameter estimates and test data VMMI predictions using Rubin’s Rules. We call this model the “SP-MI” model. Finally, we used CCA to fit a spatial linear model to the training data using only sites with observed SH scores and this model was used to predict VMMI in the test data. We call this model the “SP-CCA” model. Bootstrap imputation for SH scores was performed using the mice R package (Van Buuren & Groothuis-Oudshoorn, 2011) with VMMI and VT informing the imputations. Spatial linear models were fit using the spmodel R package (Dumelle, Higham, & Ver Hoef, 2023). For each of the SP-ALL, SP-MI, and SP-CCA approaches, we evaluated the same fixed effect and predictive performance metrics from prior case studies. In summary, this analysis enabled us to compare the effectiveness of multiple imputation and CCA to a similarly specified spatial model that had access to all the data.

From Table 10, the SP-MI parameter estimates and SEs were more similar to SP-ALL than SP-CCA. All three approaches had negative parameter estimates for both slope parameters, indicating that (1) Woody wetlands $\left({\hat{\beta }}_{1}\right)$ are associated with lower average VMMI scores and (2) higher SH scores $\left({\hat{\beta }}_{2}\right)$ are associated with lower average VMMI scores. SP-MI SEs were nearly as small as the SP-ALL SEs, but SP-CCA SEs were larger, especially for ${\hat{\beta }}_{1}$ (the woody wetland parameter). All approaches had negligible mean prediction bias and proper prediction interval coverage (for a test data sample size of 321), but SP-MI and SP-ALL had notably lower (better) RMSPE than SP-CCA, and hence they were more efficient (Table 11). Together, these results suggest that SP-MI recovered most of the information lost from the missing data, while this is not true for SP-CCA. We expect this general takeaway may also occur for spatial data with different distance measures, such as those on stream networks (Dumelle et al., 2024; Peterson & Ver Hoef, 2010; Ver Hoef & Peterson, 2010).

Though unrelated to the study of missing data on spatial regression models, the SP-ALL model significantly outperformed its nonspatial counterpart, as measured by Akaike information criterion (AIC) (Akaike, 2003) and Bayesian information criterion (BIC) (Schwarz, 1978), where a lower score indicates a more effective predictive model. The SP-ALL AIC and BIC were 5532.7 and 5546.1, respectively; the AIC and BIC of its nonspatial counterpart were 5700.5 and 5705.0, respectively. Zimmerman and Ver Hoef (2024) provide more details about why this occurs and outline the significant improvements in model fit that can be achieved by incorporating spatial covariance into a regression (i.e., linear model, generalized linear model) framework for spatial data.

Recall that fortunately, the NWCA data were collected using a spatially balanced probability sample. Probability sampling guards against the dangers of sampling preferentially. Preferential sampling occurs when the observation locations and response variable are not independent (Diggle et al., 2010). For example, one may be interested in quantifying the fish abundance in a river segment but may (consciously or unconsciously) choose to sample locations where more fish are present. Preferential sampling generally leads to poor model fit, though it is possible to improve fit by conditioning on additional variable(s) such that the observations and locations are conditionally independent (Diggle et al., 2010), similar to how MAR assumptions rely on conditioning of the observed data.

This case study, while primarily introductory and illustrative, shows that at least in some contexts, multiple imputation can be an effective tool for spatial regression modeling with missing data. This simple example had clearly defined MAR missing data in the model’s single explanatory variable. In practice, missing data may have a more complex structure (e.g., MNAR) or be present in several variables simultaneously. In this example, while spatial covariance was incorporated into the regression model, it was ignored while generating imputations. Custom methods and software should be developed to perform spatial multiple imputation, which, we believe, is a promising area of future research.

## CONCLUSION

Missing data in ecology, and science more broadly, are common and easily mishandled. There are many approaches for handling missing data, each ranging in utility and complexity. Our hope is that this work provides researchers tools that can aid the understanding and handling of missing data. Concise and explicit considerations for handling data missing data in the ecological literature are lacking, so we end by providing five considerations for ecologists and other scientists to contemplate.

**Consideration 1.** *Record missing data clearly and unambiguously. Be explicit and transparent about the amount of missing data and which methods were used to handle them*.

Options for handling missing data that are not recorded clearly and unambiguously are limited. Missing data should be coded with a unique value that cannot be confused with another measured item. For example, missing items should not be coded as a zero if it is possible the measured item can have a true value of zero. Measure variables that are related to perceived causes of missingness, even if these variables are not of primary scientific interest, as they can be used to inform missing data approaches like imputation, data augmentation, or determining contingency variables.

Strive to be explicit and transparent about the sources of missingness, the amounts of missingness, and the methods used to handle missing data. Simply ignoring the missing data without further mention, while convenient, is neither explicit nor transparent, and contributes to a lack of reproducibility and replicability in science (Baker, 2016). Following this consideration helps to ensure that scientific conclusions and decision-making processes are valid (National Academies, 2019). Some scientific disciplines (e.g., clinical trials) have encouraged this practice and provided guidance (e.g., via CONSORT, Moher et al., 2010).

**Consideration 2.** *For meaningful missing data, critically evaluate whether the ignorable (MCAR, MAR) or nonignorable (MNAR) assumption is most appropriate given the context of the research question(s) and structure of the data*.

The assumptions regarding missing data heavily inform the choice of missing data approach and validity of the resulting analysis. Making correct assumptions about the missing data and using appropriate missing data approaches helps to ensure that scientific conclusions are reliable. The MAR assumption (i.e., an ignorable assumption) requires that the variables related to missingness are accounted for and is general enough to accommodate a variety of missing data contexts. For MAR missing data, many missing data approaches exist in readily available software packages. Moreover, when data are MNAR (i.e., a nonignorable assumption) but the strength of MNAR missingness is small, MAR approaches can provide sensible solutions (Schafer & Graham, 2002). Thus, the MAR assumption can serve as a starting point for ecologists—a practical balance between complexity and feasibility. However, there are certainly scenarios where the MAR assumption fails to adequately capture the nuances of MNAR data.

**Consideration 3.** *Familiarize yourself with multiple imputation and data augmentation, both of which are versatile tools suited to handle a variety of missing data problems. Also recognize that these tools are not always the best solution for every problem*.

Multiple imputation and data augmentation are effective and widespread methods for handling missing data (both ignorable and nonignorable), a conclusion reached empirically here and repeatedly reinforced through decades of research (e.g., Little & Rubin, 2019; Rubin, 1996; Van Buuren, 2018). Multiple imputation and data augmentation are so useful because they can incorporate uncertainty in the missing items and model parameters as well as provide a theoretical framework for pooling model results. Schafer and Graham (2002) find that under the same distributional assumptions among variables, as long as the sample size and number of multiple imputations is sufficiently large, then multiple imputation approximates maximum likelihood and a fully Bayesian analysis (both data augmentation approaches). Thus they conclude that in this context, the results from these procedures will be “essentially identical.” One potential advantage of multiple imputation over data augmentation is that for multiple imputation, the imputation and analysis methods can be formally separated. One potential advantage of data augmentation over multiple imputation is that data augmentation approaches can be more efficient (i.e., less variability in estimates and predictions), especially when the number of multiple imputations is small.

Multiple imputation and data augmentation, while incredibly useful, may not be the best solution to every problem. If the proportion of missing data is small and the model is correctly specified, CCA is an efficient, straightforward approach, especially when missing data are only in the response variable. IPW adjustments (and related approaches) are effective solutions, especially when leveraging design-based inference to weight each unit based on how representative they are in the broader population. Sometimes, while multiple imputation or data augmentation may be preferred theoretically, they are too computationally intensive and (stochastic) single imputation or deterministic imputation must be used instead. Single or deterministic imputation also yields a single complete dataset that is simple to share with the public. Generally, single imputation should be preferred to deterministic imputation, as the former incorporates randomness in the imputations themselves while the latter does not. For MNAR data, specialized models that clarify how the missing data mechanisms and patterns relate to one another (e.g., a Heckman model) are most effective.

**Consideration 4.** *Include all analysis method variables in the imputation method. Thoughtfully include auxiliary variables in the imputation method*.

All variables in the analysis method should be in the imputation method (Austin et al., 2021; D’Agostino McGowan et al., 2024; He, 2010; Murray, 2018; Tilling et al., 2016). If the data were collected via a probability sample, design variables (i.e., variables that affect sampling probabilities) should be included in the imputation method (Hartman & Huang, 2024; Reiter et al., 2006) and if relevant, the analysis method. Auxiliary (i.e., additional) variables should be included in imputation methods when they are relevant (i.e., correlated) to the variable being imputed, especially when those variables are also predictive of missingness (Daniel et al., 2012). However, careful thought should be given to whether or not to include an auxiliary variable, as it is possible that an auxiliary variable introduces collider bias (Thoemmes & Rose, 2014). In many cases, including auxiliary variables in the imputation method that are unrelated to the variable(s) requiring imputation has little effect on performance of the analysis method (Collins et al., 2001).

**Consideration 5.** *Stochastic imputation methods should include in the imputation method the eventual response variable from the analysis method. Deterministic imputation methods should omit from the imputation method the eventual response variable from the analysis method*.

Stochastic imputation methods (i.e., single or multiple imputation) have random components and benefit from including the response variable from the analysis method. Omitting the response from the imputation methods can yield analysis methods that are biased, inefficient, and lack proper CI coverage. Deterministic imputation methods do not have random components and benefit from omitting the response variable from the analysis method. Including the response in the imputation methods can increase bias while decreasing efficiency and CI coverage of the analysis method. D’Agostino McGowan et al. (2024) provide a rigorous description of these results, but the intuition is guided by the fact that the stochastic imputation methods emulate properties of Bayesian modeling by conditioning on the observed response variable, while deterministic imputation methods do not share this connection.

## Supplementary Material

Supplement1

Additional supporting information can be found online in the Supporting Information section at the end of this article.

## Figures and Tables

**FIGURE 1:**
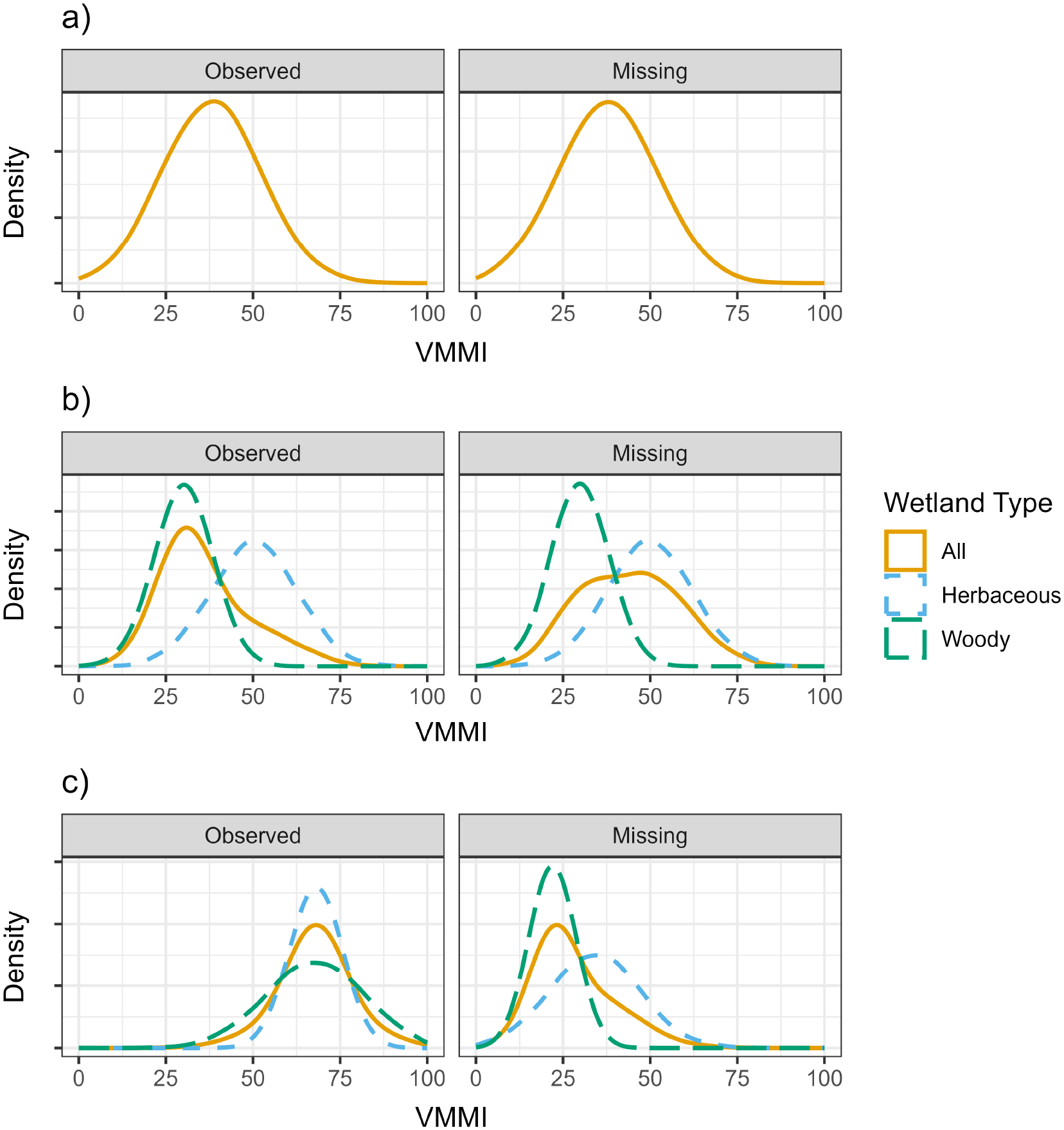
Missing completely at random (MCAR) (a), missing at random (MAR) (b), and missing not at random (MNAR) (c) distributions for hypothetical vegetation multimetric index (VMMI) data.

**FIGURE 2:**
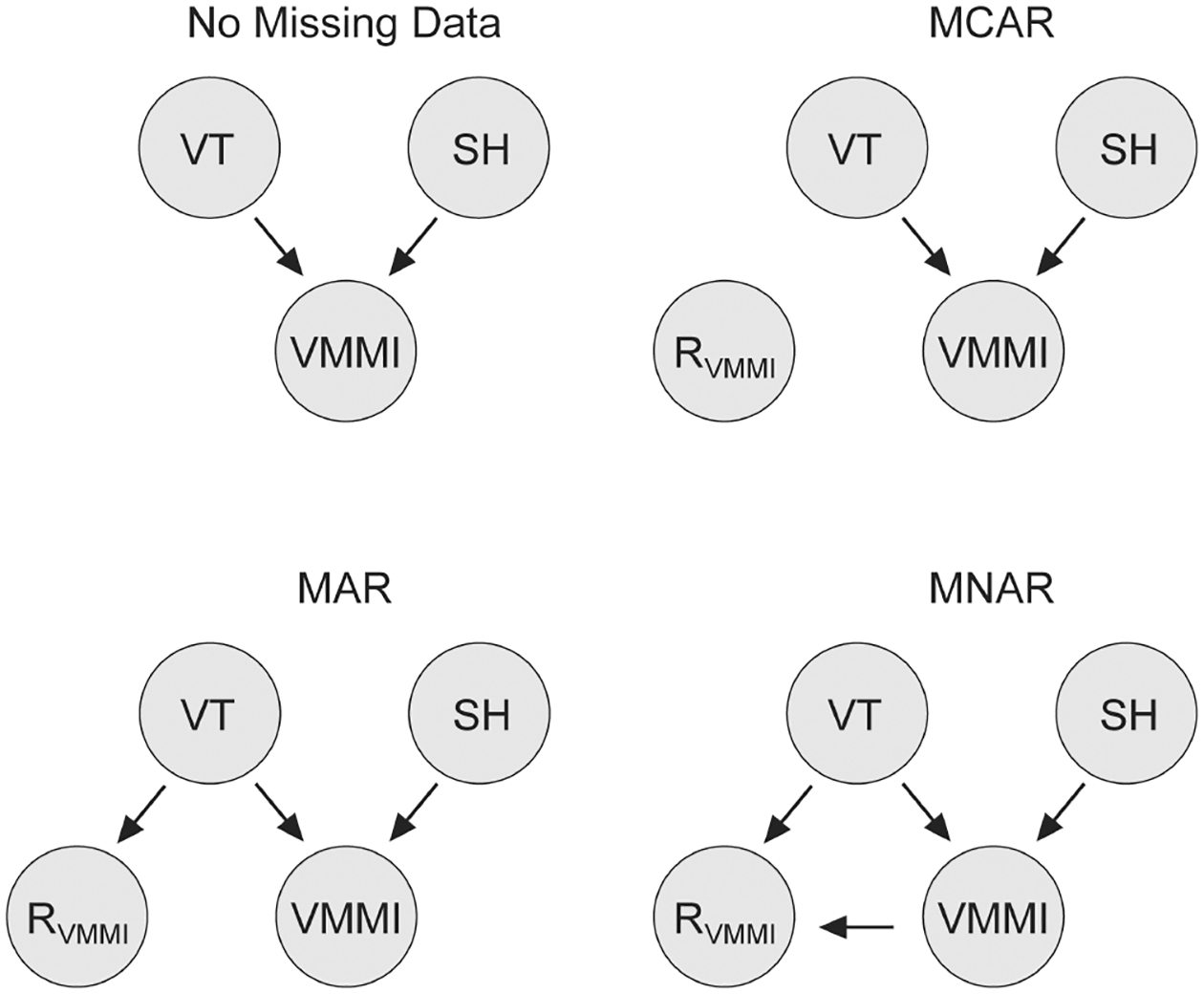
A directed acyclic graph (DAG) for the assumed relationships among vegetation type (VT), soil hardening (SH), vegetation multimetric index (VMMI), and $R_{\text{VMMI}}$ (missing VMMI data). In all scenarios, VT and SH affect VMMI. When missing VMMI data are missing completely at random (MCAR), $R_{\text{VMMI}}$ is unaffected by VT, SH, and VMMI. When missing VMMI data are missing at random (MAR), $R_{\text{VMMI}}$ is affected by VT. When missing VMMI data are missing not at random (MNAR), $R_{\text{VMMI}}$ is affected by VT and VMMI.

**FIGURE 3:**
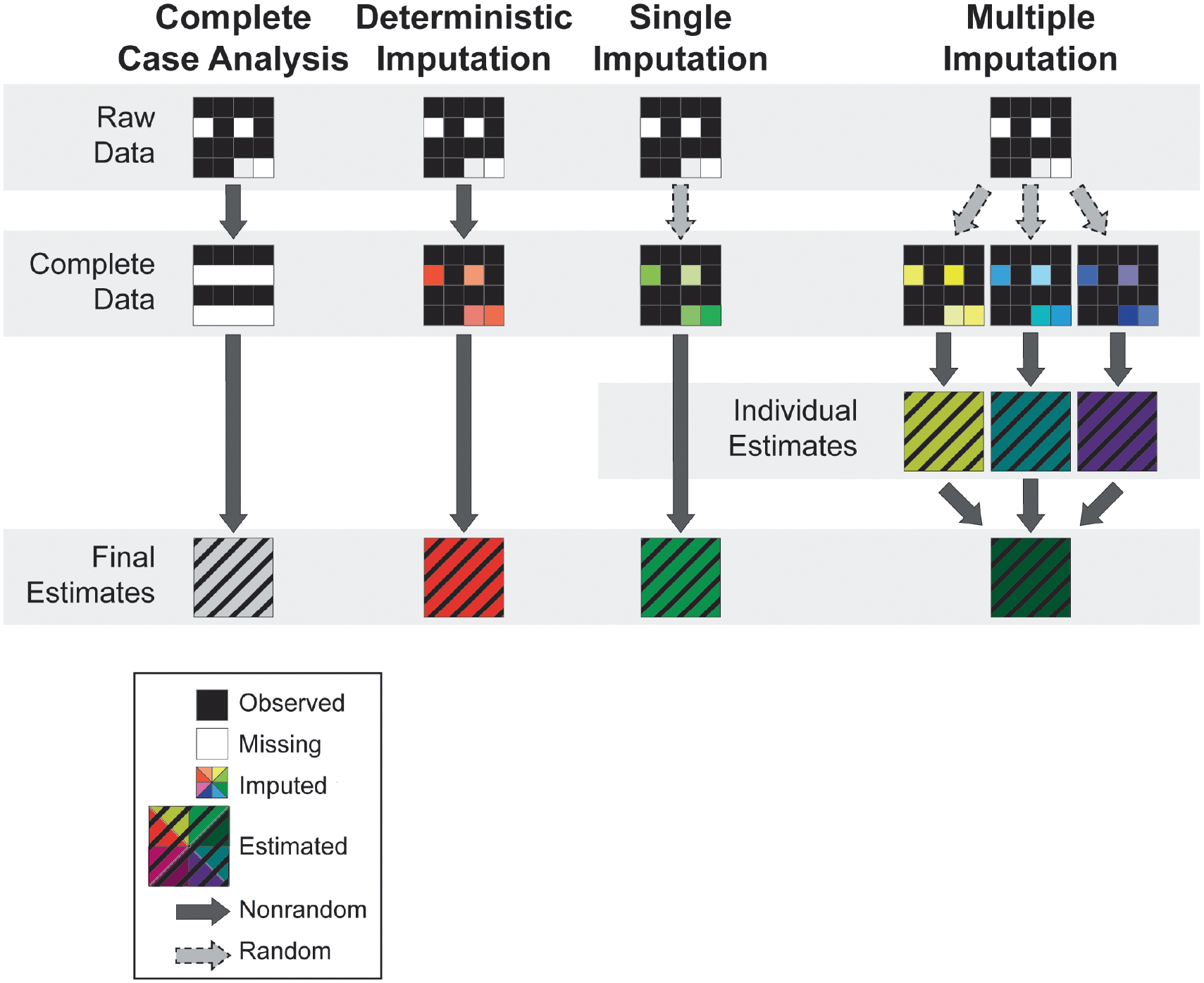
A flowchart for specific missing data approaches. Complete case analysis removes units with missing items and then implements an analysis method. Deterministic imputation fills in missing values in a nonrandom way and then implements an analysis method. Single imputation fills in missing values in a random way and then implements an analysis method. Multiple imputation pools the results from m separate single imputation steps. We label the transition from “Complete Data” to “Final Estimates” as nonrandom. We recognize that certain analysis techniques may include a random component (e.g., random initial values; Markov chain Monte Carlo [MCMC] sampling), but we implicitly assume that these perturbations make little difference in the overall analysis method conclusions.

**FIGURE 4:**
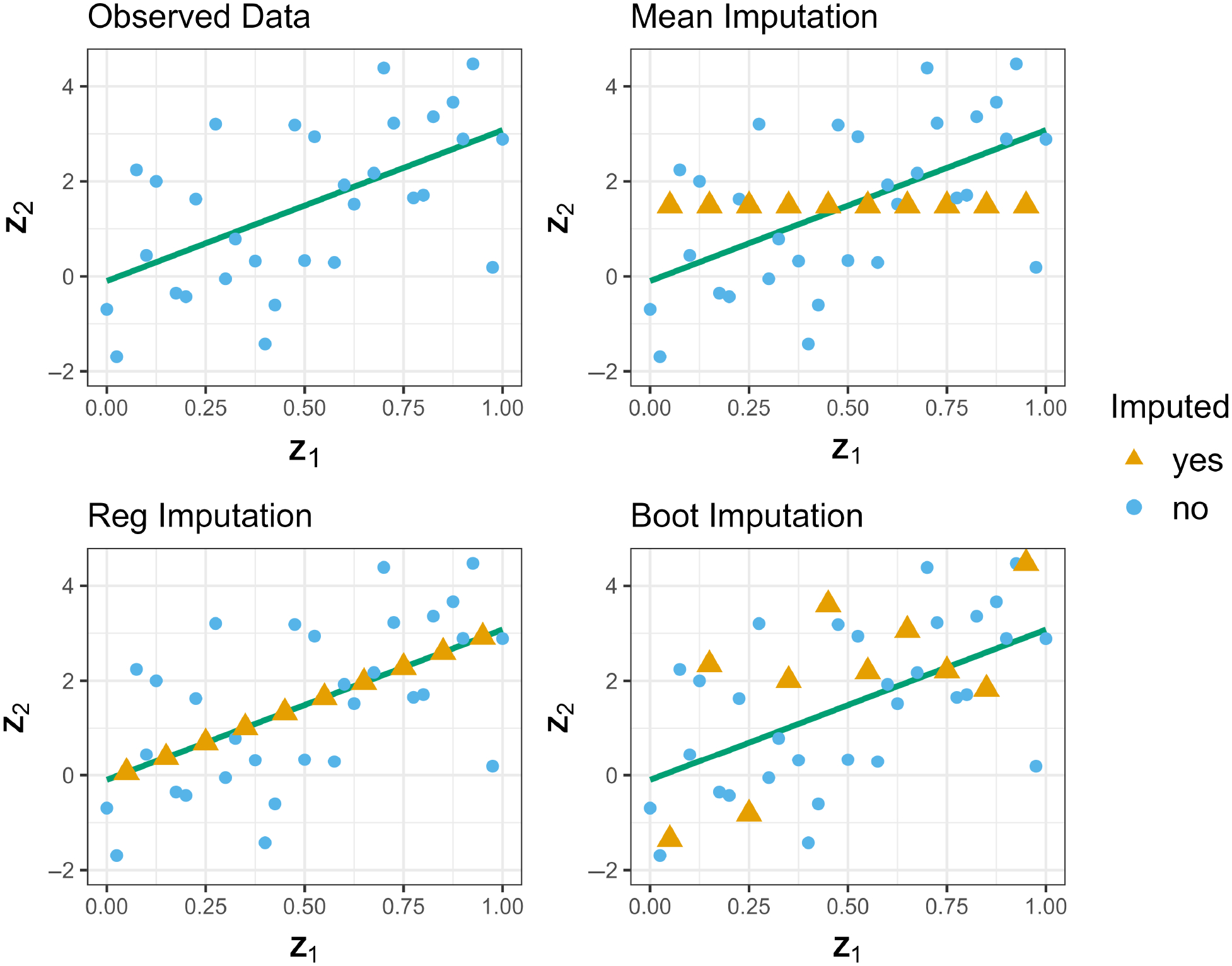
Observed and imputed data comparison. The hypothetical variable $z_{1}$ is fully observed and used to impute the hypothetical variable $z_{2}$ using mean (Mean), regression (Reg), and bootstrap (Boot) imputation. Observed items are represented by blue circles and imputed items are represented by orange triangles. The green, solid line is a linear regression slope characterizing the average effect of $z_{1}$ on $z_{2}$ using the observed data.

**FIGURE 5:**
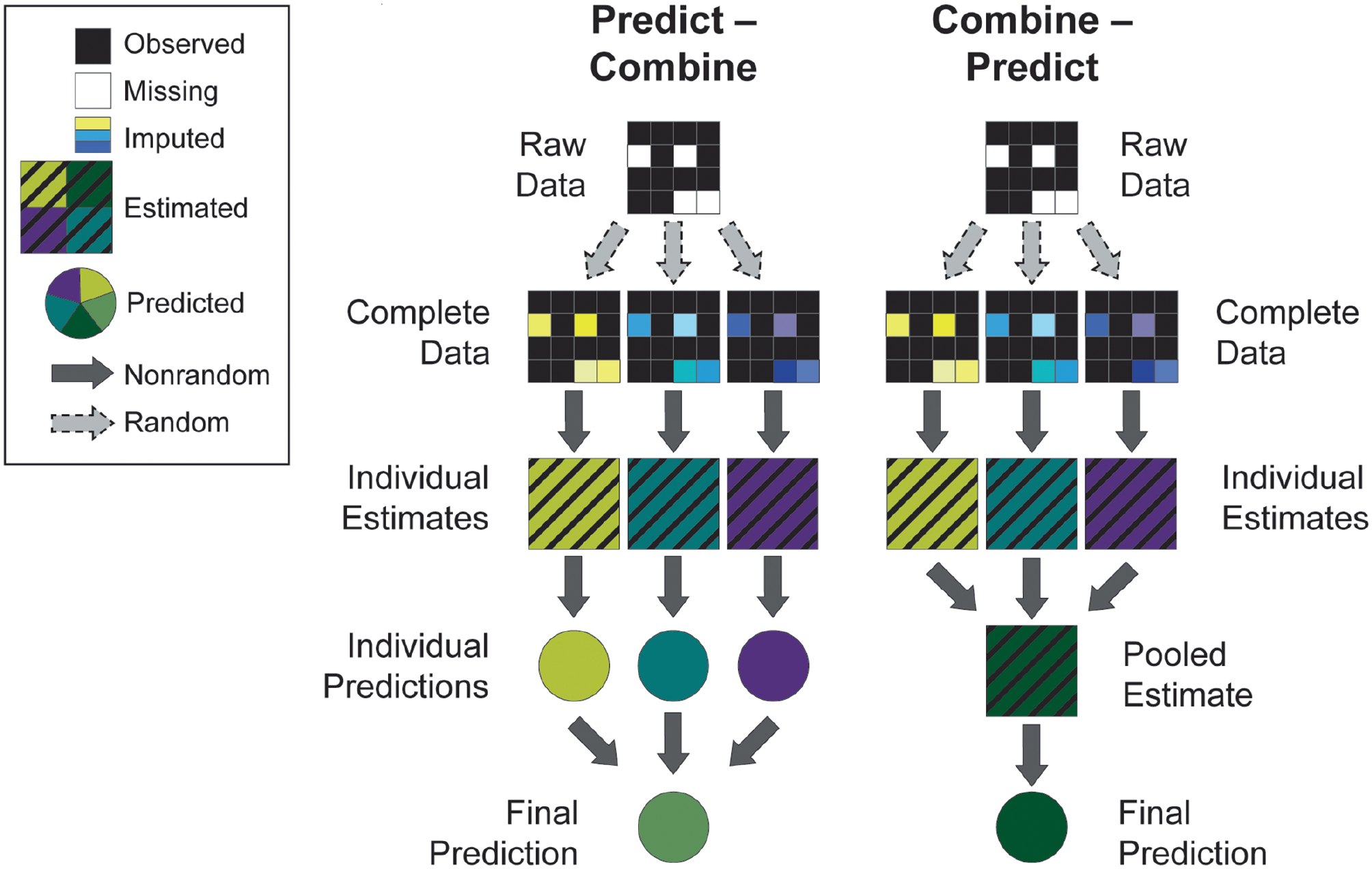
The Predict-Combine and Combine-Predict approaches to prediction using multiple imputation. In Predict-Combine, analysis methods are used to make predictions separately for each complete dataset and then pooled according to Rubin’s Rules to create a final prediction. In Combine-Predict, analysis methods are used and pooled across complete datasets, and then the pooled fit is used to create a final prediction.

**FIGURE 6:**
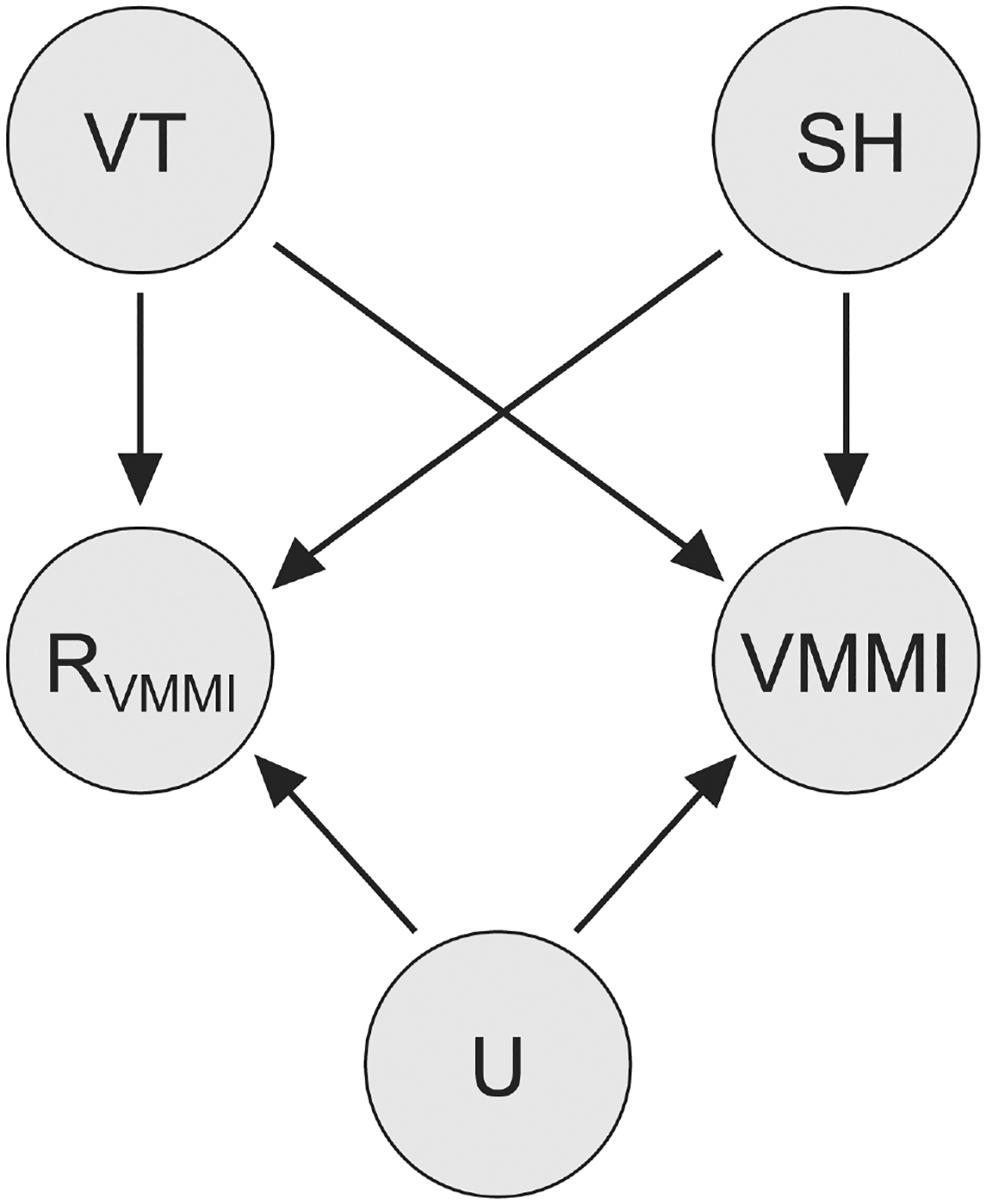
A directed acyclic graph (DAG) for the comparison of missing data methods for missing not at random (MNAR) data simulation study. SH, soil hardening; VMMI, vegetation multimetric index; VT, vegetation type. U, an observed common cause of VMMI and its missingness.

**FIGURE 7:**
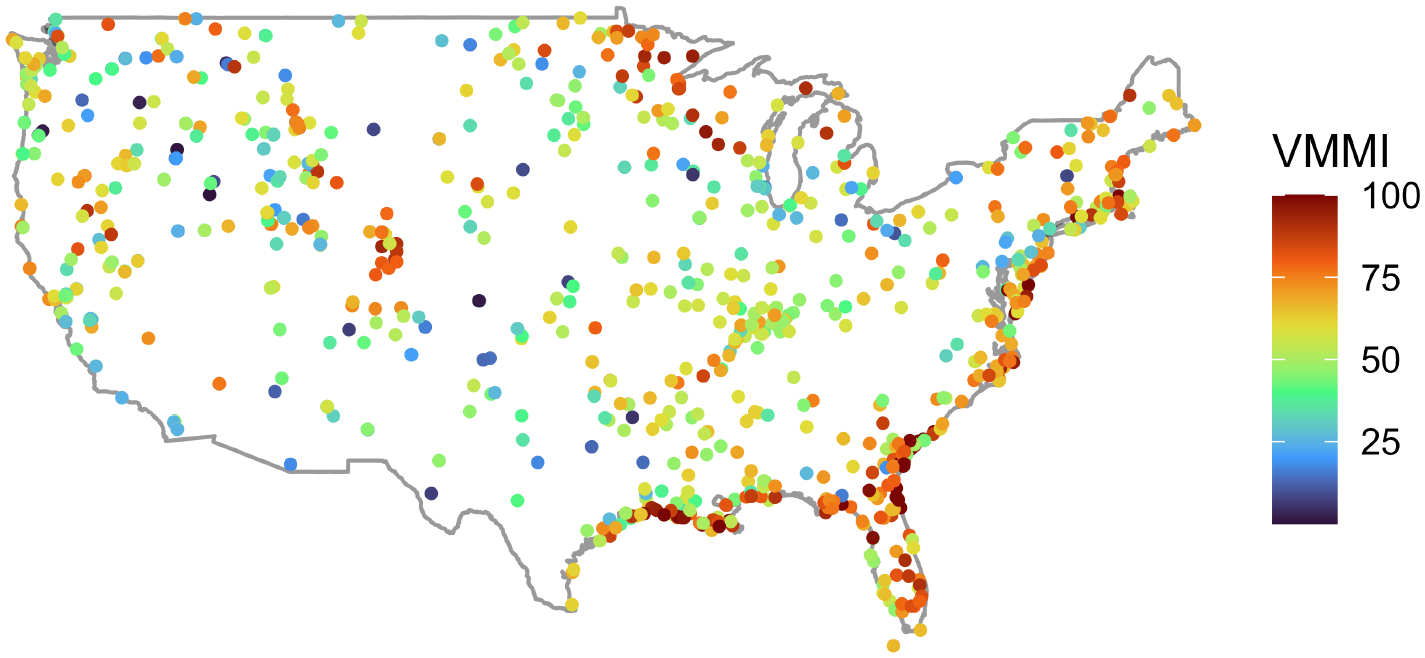
Spatial distribution of 2016 National Wetland Condition Assessment (NWCA) vegetation multimetric index (VMMI). VMMI scores range from 0 to 100. The larger the VMMI, the healthier the vegetation.

**TABLE 1:** 2016 National Wetland Condition Assessment (NWCA) variables, descriptions, and valid values. Brackets indicate a continuous range of values from the first to the last number.

Class	Name	Description	Abbreviation	Valid values
Vegetation	Type	Dominant wetland vegetation type	VT	Woody; herbaceous
Soil	Hardening	Soil hardening index	SH	[0, 424]
Vegetation	VMMI	Vegetation multimetric index	VMMI	[0, 100]

**TABLE 2:** Descriptions of missing data notation.

Notation	Description
Y	All possible values of each item
y	A single (realized) value of each item
R	All possible missingness patterns
r	A single (realized) missingness pattern
$Y_{\text{obs}}$	All possible observed subsets of Y
$y_{\text{obs}}$	A single (realized) observed subset of Y
$Y_{\text{mis}}$	All possible missing subsets of Y
$y_{\text{mis}}$	A single (realized) missing subset of Y

**TABLE 3:** Missing data approaches and abbreviations used in the simulation study.

Approach	Abbreviation	Imputations	Parameters
Complete case analysis	CCA	⋯	⋯
Mean imputation	Mean	Deterministic	Deterministic
Regression imputation	Reg	Deterministic	Deterministic
Nearest neighbor imputation	NN	Deterministic	Deterministic
Stochastic regression imputation (single)	StReg-S	Stochastic	Deterministic
Predictive mean matching Type-0 imputation (single)	PMMT0-S	Stochastic	Deterministic
Bootstrap regression imputation (Single)	Boot-S	Stochastic	Stochastic
Predictive mean matching Type-1 imputation (single)	PMMT1-S	Stochastic	Stochastic
Stochastic regression imputation (multiple)	StReg-M	Stochastic	Deterministic
Predictive mean matching Type-0 imputation (multiple)	PMMT0-M	Stochastic	Deterministic
Bootstrap regression imputation (multiple)	Boot-M	Stochastic	Stochastic
Predictive mean matching Type-1 imputation (multiple)	PMMT1-M	Stochastic	Stochastic
Fully Bayesian data augmentation	FBDA	Stochastic	Stochastic

*Note*: The “Imputations” column clarifies whether the imputation process is deterministic (i.e., nonrandom) or stochastic (i.e., random). The “Parameters” column clarifies whether the parameters used to fill out missing data items are deterministic or stochastic.

**TABLE 4:** For the study of missing at random approaches from A comparison of various missing data approaches for MAR missing data, missing data approach inferential performance across 2000 independent simulation trials.

	$\beta_{1}$				$\beta_{2}$			
Approach	MBias	RMSE	SE	Cover95	MBias	RMSE	SE	Cover95
**CCA**	0.00	0.49	0.50	**0.95**	−0.00	0.26	0.27	**0.95**
Mean	0.59	0.62	0.20	0.16	0.59	0.61	0.15	0.06
Reg	0.00	0.47	0.09	0.31	−0.31	0.40	0.06	0.12
NN	0.04	0.51	0.24	0.65	0.32	0.45	0.13	0.38
StReg-S	0.00	0.46	0.21	0.60	0.02	0.26	0.11	0.59
PMMT0-S	0.08	0.46	0.21	0.62	0.10	0.31	0.11	0.55
Boot-S	−0.00	0.54	0.20	0.52	0.00	0.28	0.10	0.55
PMMT1-S	0.07	0.52	0.21	0.56	0.12	0.33	0.11	0.53
StReg-M	−0.00	0.40	0.31	0.87	0.02	0.22	0.19	0.91
PMMT0-M	0.08	0.40	0.32	0.88	0.11	0.25	0.22	0.90
**Boot-M**	0.00	0.40	0.40	**0.95**	0.00	0.22	0.23	**0.95**
**PMMT1-M**	0.07	0.39	0.41	**0.96**	0.12	0.26	0.24	**0.94**
**FBDA**	0.00	0.38	0.39	**0.95**	0.04	0.22	0.24	**0.95**

*Note*: Mean bias (MBias), root-mean-squared error (RMSE), mean SE, and 95% CI coverage (Cover95) are reported separately for the $\beta_{1}$ and $\beta_{2}$ slope parameters. The “Approach” and “Cover95” columns are bolded when the Approach has proper Cover95 (i.e., between 0.94 and 0.96). Please refer to Table 3 for the expansions of “Approach” names.

**TABLE 5:** For the study of missing at random approaches from A comparison of various missing data approaches for MAR missing data, missing data approach prediction performance across 2000 independent simulation trials.

	Predictions			
Approach	MPBias	RMSPE	SE	PCover95
**CCA**	−0.01	1.08	1.18	**0.96**
Mean	−0.21	1.24	0.98	0.88
Reg	0.00	1.12	0.46	0.57
**NN**	−0.02	1.17	1.20	**0.96**
StReg-S	−0.00	1.09	1.03	0.92
PMMT0-S	−0.02	1.10	1.05	0.93
Boot-S	−0.02	1.11	1.00	0.90
PMMT1-S	−0.02	1.12	1.06	0.92
**StReg-M**	−0.01	1.06	1.05	**0.94**
**PMMT0-M**	−0.02	1.07	1.08	**0.94**
**Boot-M**	−0.01	1.06	1.05	**0.94**
**PMMT1-M**	−0.02	1.07	1.11	**0.95**
**FBDA**	−0.01	1.06	1.13	**0.95**

*Note*: Mean prediction bias (MPBias), root-mean-squared-prediction error (RMSPE), mean prediction SEs, and 95% prediction interval coverage (PCover95) are reported for predictions. The “Approach” and “PCover95” columns are bolded when the Approach has proper PCover95 (i.e., between 0.94 and 0.96). Please refer to Table 3 for the expansions of “Approach” names.

**TABLE 6:** For the study of including or omitting the analysis method response variable in the imputation method from The impact of including the response variable in an imputation method for MAR missing data, missing data approach inferential performance across 2000 independent simulation trials.

		$\beta_{1}$				$\beta_{2}$			
Approach	Include y	MBias	RMSE	SE	Cover95	MBias	RMSE	SE	Cover95
Reg	No	−0.00	0.45	0.18	0.58	0.00	0.26	0.13	0.69
Reg	Yes	0.01	0.47	0.09	0.31	−0.31	0.40	0.05	0.12
Boot-M	No	−0.00	0.42	0.45	0.97	0.69	0.70	0.22	0.03
**Boot-M**	**Yes**	0.00	0.40	0.39	**0.95**	0.00	0.22	0.22	**0.96**

*Note*: Mean bias (MBias), root-mean-squared error (RMSE), mean SEs, and 95% CI coverage (Cover95) are reported separately for the $\beta_{1}$ and $\beta_{2}$ slope parameters. The “Approach,” “Include y,” and “Cover95” columns are bolded when the Approach has proper Cover95 (i.e., between 0.94 and 0.96). Please refer to Table 3 for the expansions of “Approach” names.

**TABLE 7:** For the study of including or omitting the analysis method response variable in the imputation method from The impact of including the response variable in an imputation method for MAR missing data, missing data approach prediction performance across 2000 independent simulation trials.

		Predictions			
Approach	Include y	MPBias	RMSPE	SE	PCover95
Reg	No	−0.01	1.08	0.17	0.25
Reg	Yes	0.01	1.13	0.08	0.12
Boot-M	No	−0.02	1.25	1.43	0.97
**Boot-M**	**Yes**	−0.01	1.06	1.04	**0.94**

*Note*: Mean prediction bias (MPBias), root-mean-squared-prediction error (RMSPE), mean prediction SEs, and 95% prediction interval coverage (PCover95) are reported for predictions. The “Approach,” “Include y,” and “PCover95” columns are bolded when the Approach has proper PCover95 (i.e., between 0.94 and 0.96). Please refer to Table 3 for the expansions of “Approach” names.

**TABLE 8:** For the study of missing at random (MAR) and missing not at random (MNAR) approaches from A comparison of various missing data approaches for MNAR missing data, missing data approach inferential performance across 2000 independent simulation trials.

		$\beta_{1}$				$\beta_{2}$			
Approach	MNAR amount	MBias	RMSE	SE	Cover95	MBias	RMSE	SE	Cover95
MAR	Large	−0.37	0.43	0.24	0.68	−0.38	0.41	0.14	0.25
Heckman	Large	−0.18	0.30	0.28	0.92	−0.16	0.23	0.20	0.91
MAR	Moderate	−0.22	0.34	0.28	0.89	−0.21	0.27	0.17	0.75
**Heckman**	**Moderate**	−0.14	0.29	0.32	**0.96**	−0.10	0.20	0.24	0.98
**MAR**	**Small**	−0.07	0.27	0.29	**0.95**	−0.06	0.18	0.17	**0.94**
Heckman	Small	−0.05	0.26	0.33	0.98	−0.02	0.18	0.25	0.99

*Note*: Mean bias (MBias), root-mean-squared error (RMSE), and 95% CI coverage (Cover95) are reported separately for the $\beta_{1}$ and $\beta_{2}$ slope parameters. The “Approach” and “Cover95” columns are bolded when the Approach has proper Cover95 (i.e., between 0.94 and 0.96).

**TABLE 9:** For the study of missing at random (MAR) and missing not at random (MNAR) approaches from A comparison of various missing data approaches for MNAR missing data, missing data approach prediction performance across 2000 independent simulation trials.

		Predictions			
Approach	MNAR amount	MPBias	RMSPE	SE	PCover95
MAR	Large	0.68	1.32	0.85	0.79
Heckman	Large	0.23	1.11	1.02	0.93
MAR	Moderate	0.37	1.12	1.00	0.91
**Heckman**	**Moderate**	0.17	1.05	1.13	**0.96**
**MAR**	**Small**	0.08	1.03	1.05	**0.95**
Heckman	Small	0.00	1.02	1.17	0.97

*Note*: Mean prediction bias (MPBias), root-mean-squared-prediction error (RMSPE), mean prediction SEs, and 95% prediction interval coverage (PCover95) are reported for predictions. The “Approach,” “Include y,” and “PCover95” columns are bolded when the Approach has proper PCover95 (i.e., between 0.94 and 0.96).

**TABLE 10:** For the spatial model applied to the vegetation multimetric index (VMMI) data from The effect of missing data on a spatial regression model, slope parameter estimates (Est.) and SEs.

	SP-ALL		SP-MI		SP-CCA	
Parameter	Est.	SE	Est.	SE	Est.	SE
$\beta_{1}$ (woody wetland)	−10.505	1.568	−10.589	1.573	−13.801	2.268
$\beta_{2}$ (soil hardening)	−0.088	0.036	−0.130	0.042	−0.134	0.050

*Note*: The SP-ALL approach uses all the data. The SP-MI approach uses multiple imputation after simulating missingness. The SP-CCA approach uses complete case analysis after simulating missingness.

**TABLE 11:** For the spatial model applied to the vegetation multimetric index (VMMI) data from The effect of missing data on a spatial regression model, mean prediction bias (MPBias), root-mean-squared-prediction error (RMSPE), mean prediction SEs, and 95% prediction interval coverage (PCover95).

	Predictions			
Approach	MPBias	RMSPE	SE	PCover95
SP-ALL	0.222	16.728	17.010	0.956
SP-MI	−0.231	16.748	16.987	0.956
SP-CCA	1.950	17.592	18.003	0.966

*Note*: The SP-ALL approach uses all the data. The SP-MI approach uses multiple imputation after simulating missingness. The SP-CCA approach uses complete case analysis after simulating missingness.

## Data Availability

Data and code (Dumelle, 2025) are available on Zenodo at https://doi.org/10.5281/zenodo.17101244.

## References

[R1] Abayomi K, Gelman A, and Levy M. 2008. “Diagnostics for Multivariate Imputations.” Journal of the Royal Statistical Society: Series C: Applied Statistics 57: 273–291.

[R2] Akaike H 2003. “A New Look at the Statistical Model Identification.” IEEE Transactions on Automatic Control 19: 716–723.

[R3] Allison PD 2005. “Imputation of Categorical Variables with PROC MI.” SUGI 30 Proceedings 113: 1–14.

[R4] Andridge RR, and Little RJ. 2010. “A Review of Hot Deck Imputation for Survey Non-response.” International Statistical Review 78: 40–64. 21743766 10.1111/j.1751-5823.2010.00103.x PMC3130338

[R5] Andridge RR, and Little RJ. 2011. “Proxy Pattern-Mixture Analysis for Survey Nonresponse.” Journal of Official Statistics 27: 153.

[R6] Andridge RR, and Little RJ. 2020. “Proxy Pattern-Mixture Analysis for a Binary Variable Subject to Nonresponse.” Journal of Official Statistics 36: 703–728.

[R7] Austin PC 2011. “An Introduction to Propensity Score Methods for Reducing the Effects of Confounding in Observational Studies.” Multivariate Behavioral Research 46: 399–424. 21818162 10.1080/00273171.2011.568786 PMC3144483

[R8] Austin PC, White IR, Lee DS, and van Buuren S. 2021. “Missing Data in Clinical Research: A Tutorial on Multiple Imputation.” Canadian Journal of Cardiology 37: 1322–31. 33276049 10.1016/j.cjca.2020.11.010 PMC8499698

[R9] Azur MJ, Stuart EA, Frangakis C, and Leaf PJ. 2011. “Multiple Imputation by Chained Equations: What Is it and how Does it Work?” International Journal of Methods in Psychiatric Research 20: 40–49. 21499542 10.1002/mpr.329 PMC3074241

[R10] Bailey MA 2024. Polling at a Crossroads: Rethinking Modern Survey Research. Cambridge: Cambridge University Press.

[R11] Baker M 2016. “1,500 Scientists Lift the Lid on Reproducibility.” Nature 533: 452–54. 27225100 10.1038/533452a

[R12] Barnard J, and Rubin DB. 1999. “Small-Sample Degrees of Freedom with Multiple Imputation.” Biometrika 86: 948–955.

[R13] Beck J, Böller M, Erhardt A, and Schwanghart W. 2014. “Spatial Bias in the GBIF Database and its Effect on Modeling Species’ Geographic Distributions.” Ecological Informatics 19: 10–15.

[R14] Bennett DA 2001. “How Can I Deal with Missing Data in my Study?” Australian and New Zealand Journal of Public Health 25: 464–69. 11688629

[R15] Bodner TE 2008. “What Improves with Increased Missing Data Imputations?” Structural Equation Modeling: A Multidisciplinary Journal 15: 651–675.

[R16] Bondarenko I, and Raghunathan T. 2016. “Graphical and Numerical Diagnostic Tools to Assess Suitability of Multiple Imputations and Imputation Models.” Statistics in Medicine 35: 3007–20. 26952693 10.1002/sim.6926

[R17] Bowler DE, Boyd RJ, Callaghan CT, Robinson RA, Isaac NJ, and Pocock MJ. 2024. “Treating Gaps and Biases in Biodiversity Data as a Missing Data Problem.” Biological Reviews 100, no. 1: 50–67. 39114921 10.1111/brv.13127 PMC11718628

[R18] Box GE, and Tiao GC. 2011. Bayesian Inference in Statistical Analysis. Hoboken, NJ: John Wiley & Sons.

[R19] Boyd RJ, Botham M, Dennis E, Fox R, Harrower C, Middlebrook I, Roy DB, and Pescott OL. 2025. “Using Causal Diagrams and Superpopulation Models to Correct Geographic Biases in Biodiversity Monitoring Data.” Methods in Ecology and Evolution 16: 332–344.

[R20] Boyd RJ, Powney GD, and Pescott OL. 2023. “We Need to Talk about Nonprobability Samples.” Trends in Ecology & Evolution 38: 521–531. 36775795 10.1016/j.tree.2023.01.001

[R21] Boyd RJ, Stewart GB, and Pescott OL. 2024. “Descriptive Inference Using Large, Unrepresentative Nonprobability Samples: An Introduction for Ecologists.” Ecology 105: e4214. 38088061 10.1002/ecy.4214 PMC10929663

[R22] Brick JM, and Kalton G. 1996. “Handling Missing Data in Survey Research.” Statistical Methods in Medical Research 5: 215–238. 8931194 10.1177/096228029600500302

[R23] Brus D, and De Gruijter J. 1997. “Random Sampling or Geostatistical Modelling? Choosing between Design-Based and Model-Based Sampling Strategies for Soil (with Discussion).” Geoderma 80: 1–44.

[R24] Buck SF 1960. “A Method of Estimation of Missing Values in Multivariate Data Suitable for Use with an Electronic Computer.” Journal of the Royal Statistical Society. Series B, Statistical Methodology 22: 302–6.

[R25] Bürkner PC 2017. “brms: An R Package for Bayesian Multilevel Models Using Stan.” Journal of Statistical Software 80: 1–28.

[R26] Cao W, Tsiatis AA, and Davidian M. 2009. “Improving Efficiency and Robustness of the Doubly Robust Estimator for a Population Mean with Incomplete Data.” Biometrika 96: 723–734. 20161511 10.1093/biomet/asp033 PMC2798744

[R27] Carpenter B, Gelman A, Hoffman MD, Lee D, Goodrich B, Betancourt M, Brubaker MA, Guo J, Li P, and Riddell A. 2017. “Stan: A Probabilistic Programming Language.” Journal of Statistical Software 76, no. 1: 1–32. 36568334 10.18637/jss.v076.i01 PMC9788645

[R28] Carpenter JR, Bartlett JW, Morris TP, Wood AM, Quartagno M, and Kenward MG. 2023. Multiple Imputation and its Application. Hoboken, NJ: John Wiley & Sons.

[R29] Carpenter JR, and Smuk M. 2021. “Missing Data: A Statistical Framework for Practice.” Biometrical Journal 63: 915–947. 33624862 10.1002/bimj.202000196 PMC7615108

[R30] Casella G, and Berger R. 2024. Statistical Inference. Boca Raton, FL: CRC Press.

[R31] Casella G, and George EI. 1992. “Explaining the Gibbs Sampler.” The American Statistician 46: 167–174.

[R32] Cassel CM, Särndal CE, and Wretman JH. 1976. “Some Results on Generalized Difference Estimation and Generalized Regression Estimation for Finite Populations.” Biometrika 63: 615–620.

[R33] Cham H, and West SG. 2016. “Propensity Score Analysis with Missing Data.” Psychological Methods 21: 427–445. 26962757 10.1037/met0000076

[R34] Chamberlain G 1986. “Asymptotic Efficiency in Semi-Parametric Models with Censoring.” Journal of Econometrics 32: 189–218. 10.1016/0304-4076(86)90038-2.

[R35] Chen J, and Shao J. 2000. “Nearest Neighbor Imputation for Survey Data.” Journal of Official Statistics 16: 113.

[R36] Chib S, and Greenberg E. 1995. “Understanding the Metropolis-Hastings Algorithm.” The American Statistician 49: 327–335.

[R37] Chib S, Greenberg E, and Jeliazkov I. 2009. “Estimation of Semiparametric Models in the Presence of Endogeneity and Sample Selection.” Journal of Computational and Graphical Statistics 18: 321–348.

[R38] Choi J, Dekkers OM, and le Cessie S. 2019. “A Comparison of Different Methods to Handle Missing Data in the Context of Propensity Score Analysis.” European Journal of Epidemiology 34: 23–36. 30341708 10.1007/s10654-018-0447-z PMC6325992

[R39] Collins LM, Schafer JL, and Kam CM. 2001. “A Comparison of Inclusive and Restrictive Strategies in Modern Missing Data Procedures.” Psychological Methods 6: 330–351. 11778676

[R40] Conn PB, Johnson DS, London JM, and Boveng PL. 2012. “Accounting for Missing Data when Assessing Availability in Animal Population Surveys: An Application to Ice-Associated Seals in the Bering Sea.” Methods in Ecology and Evolution 3: 1039–46.

[R41] Cowardin L 1979. Classification of Wetlands and Deepwater Habitats of the United States. Jamestown, ND: Northern Prairie Wildlife Research Center.

[R42] Cressie N 1993. Statistics for Spatial Data. Hoboken, NJ: Wiley.

[R43] Cressie N, Calder CA, Clark JS, Ver Hoef JM, and Wikle CK. 2009. “Accounting for Uncertainty in Ecological Analysis: The Strengths and Limitations of Hierarchical Statistical Modeling.” Ecological Applications 19: 553–570. 19425416 10.1890/07-0744.1

[R44] D’Agostino RB Jr., and Rubin DB. 2000. “Estimating and Using Propensity Scores with Partially Missing Data.” Journal of the American Statistical Association 95: 749–759.

[R45] D’Agostino McGowan L, Lotspeich SC, and Hepler SA. 2024. “The “Why” behind Including “y” in your Imputation Model.” Statistical Methods in Medical Research 33: 996–1020. 38625810 10.1177/09622802241244608

[R46] Daniel RM, Kenward MG, Cousens SN, and De Stavola BL. 2012. “Using Causal Diagrams to Guide Analysis in Missing Data Problems.” Statistical Methods in Medical Research 21: 243–256. 21389091 10.1177/0962280210394469

[R47] De Leeuw ED 2001. “Reducing Missing Data in Surveys: An Overview of Methods.” Quality and Quantity 35: 147–160.

[R48] Demirtas H, Freels SA, and Yucel RM. 2008. “Plausibility of Multivariate Normality Assumption when Multiply Imputing Non-gaussian Continuous Outcomes: A Simulation Assessment.” Journal of Statistical Computation and Simulation 78: 69–84.

[R49] Dempster AP, Laird NM, and Rubin DB. 1977. “Maximum Likelihood from Incomplete Data Via the EM Algorithm.” Journal of the Royal Statistical Society, Series B: Statistical Methodology 39: 1–22.

[R50] Deng Y, Chang C, Ido MS, and Long Q. 2016. “Multiple Imputation for General Missing Data Patterns in the Presence of High-Dimensional Data.” Scientific Reports 6: 21689. 26868061 10.1038/srep21689 PMC4751511

[R51] Diggle PJ, Menezes R, and Su TL. 2010. “Geostatistical Inference under Preferential Sampling.” Journal of the Royal Statistical Society: Series C: Applied Statistics 59: 191–232.

[R52] Donders ART, Van Der Heijden GJ, Stijnen T, and Moons KG. 2006. “A Gentle Introduction to Imputation of Missing Values.” Journal of Clinical Epidemiology 59: 1087–91. 16980149 10.1016/j.jclinepi.2006.01.014

[R53] Dray S, and Josse J. 2015. “Principal Component Analysis with Missing Values: A Comparative Survey of Methods.” Plant Ecology 216: 657–667.

[R54] Duane S, Kennedy AD, Pendleton BJ, and Roweth D. 1987. “Hybrid Monte Carlo.” Physics Letters B 195: 216–222.

[R55] Dumelle M 2025. “USEPA/missing.data.in.ecology: Publication Release (0.0.1).” Zenodo. 10.5281/zenodo.17101244.

[R56] Dumelle M, Higham M, and Ver Hoef JM. 2023. “spmodel: Spatial Statistical Modeling and Prediction in R.” PLoS One 18: e0282524. 36893090 10.1371/journal.pone.0282524 PMC9997982

[R57] Dumelle M, Higham M, Ver Hoef JM, Olsen AR, and Madsen L. 2022. “A Comparison of Design-Based and Model-Based Approaches for Finite Population Spatial Sampling and Inference.” Methods in Ecology and Evolution 13: 2018–29. 36340863 10.1111/2041-210X.13919 PMC9628259

[R58] Dumelle M, Kincaid T, Olsen AR, and Weber M. 2023. “spsurvey: Spatial Sampling Design and Analysis in R.” Journal of Statistical Software 105: 1–29. 36798141 10.18637/jss.v105.i03 PMC9926341

[R59] Dumelle M, Peterson EE, Ver Hoef JM, Pearse A, and Isaak DJ. 2024. “SSN2: The Next Generation of Spatial Stream Network Modeling in R.” Journal of Open Source Software 9(99): 6389. 10.21105/joss.06389 PMC11346377 39193024

[R60] Eckman S, Kreuter F, Kirchner A, Jäckle A, Tourangeau R, and Presser S. 2014. “Assessing the Mechanisms of Misreporting to Filter Questions in Surveys.” Public Opinion Quarterly 78: 721–733.

[R61] Eddings W, and Marchenko Y. 2012. “Diagnostics for Multiple Imputation in Stata.” The Stata Journal 12: 353–367.

[R62] Efron B 1994. “Missing Data, Imputation, and the Bootstrap.” Journal of the American Statistical Association 89: 463–475.

[R63] Efron B, and Tibshirani RJ. 1994. An Introduction to the Bootstrap. Boca Raton, FL: CRC Press.

[R64] Ellington EH, Bastille-Rousseau G, Austin C, Landolt KN, Pond BA, Rees EE, Robar N, and Murray DL. 2015. “Using Multiple Imputation to Estimate Missing Data in Meta-Regression.” Methods in Ecology and Evolution 6: 153–163.

[R65] Elliott MR, and Valliant R. 2017. “Inference for Nonprobability Samples.” Statistical Science 32: 249–264.

[R66] Enders CK 2022. Applied Missing Data Analysis. New York, NY: Guilford Publications.

[R67] Eskelson BN, Temesgen H, Lemay V, Barrett TM, Crookston NL, and Hudak AT. 2009. “The Roles of Nearest Neighbor Methods in Imputing Missing Data in Forest Inventory and Monitoring Databases.” Scandinavian Journal of Forest Research 24: 235–246.

[R68] Fox J 2019. Regression Diagnostics: An Introduction. Thousand Oaks, CA: Sage Publications.

[R69] Frangakis CE, and Rubin DB. 2002. “Principal Stratification in Causal Inference.” Biometrics 58: 21–29. 11890317 10.1111/j.0006-341x.2002.00021.x PMC4137767

[R70] Fraser GE, Yan R, Butler TL, Jaceldo-Siegl K, Beeson WL, and Chan J. 2009. “Missing Data in a Long Food Frequency Questionnaire: Are Imputed Zeroes Correct?” Epidemiology 20: 289–294. 19177024 10.1097/EDE.0b013e31819642c4 PMC2745716

[R71] Galimard JE, Chevret S, Protopopescu C, and Resche-Rigon M. 2016. “A Multiple Imputation Approach for MNAR Mechanisms Compatible with Heckman’s Model.” Statistics in Medicine 35: 2907–20. 26893215 10.1002/sim.6902

[R72] Gareth J, Daniela W, Trevor H, and Robert T. 2013. An Introduction to Statistical Learning: With Applications in R New York, NY: Springer.

[R73] Gelfand AE, and Smith AF. 1990. “Sampling-Based Approaches to Calculating Marginal Densities.” Journal of the American Statistical Association 85: 398–409.

[R74] Gelman A 2007. “Struggles with Survey Weighting and Regression Modeling.” Statistical Science 22: 153–164.

[R75] Gelman A, Carlin JB, Stern HS, and Rubin DB. 1995. Bayesian Data Analysis. Boca Raton, FL: Chapman and Hall/CRC.

[R76] Gelman A, King G, and Liu C. 1998. “Not Asked and Not Answered: Multiple Imputation for Multiple Surveys.” Journal of the American Statistical Association 93: 846–857.

[R77] Gelman A, Van Mechelen I, Verbeke G, Heitjan DF, and Meulders M. 2005. “Multiple Imputation for Model Checking: Completed-Data Plots with Missing and Latent Data.” Biometrics 61: 74–85. 15737080 10.1111/j.0006-341X.2005.031010.x

[R78] Geman S, and Geman D. 1984. “Stochastic Relaxation, Gibbs Distributions, and the Bayesian Restoration of Images.” IEEE Transactions on Pattern Analysis and Machine Intelligence PAMI-6: 721–741. 10.1109/tpami.1984.4767596 22499653

[R79] Gleason TC, and Staelin R. 1975. “A Proposal for Handling Missing Data.” Psychometrika 40: 229–252.

[R80] Graham JW 2009. “Missing Data Analysis: Making it Work in the Real World.” Annual Review of Psychology 60: 549–576. 10.1146/annurev.psych.58.110405.085530 18652544

[R81] Graham JW 2012. Missing Data: Analysis and Design. New York, NY: Springer Science & Business Media.

[R82] Graham JW, Olchowski AE, and Gilreath TD. 2007. “How Many Imputations Are Really Needed? some Practical Clarifications of Multiple Imputation Theory.” Prevention Science 8: 206–213. 17549635 10.1007/s11121-007-0070-9

[R83] Graham JW, Taylor BJ, Olchowski AE, and Cumsille PE. 2006. “Planned Missing Data Designs in Psychological Research.” Psychological Methods 11: 323–343. 17154750 10.1037/1082-989X.11.4.323

[R84] Greenland S, Pearl J, and Robins JM. 1999. “Causal Diagrams for Epidemiologic Research.” Epidemiology 10: 37–48. 9888278

[R85] Groenwold RH, White IR, Donders ART, Carpenter JR, Altman DG, and Moons KG. 2012. “Missing Covariate Data in Clinical Research: When and when Not to Use the Missing-Indicator Method for Analysis.” Canadian Medical Association Journal 184: 1265–69. 22371511 10.1503/cmaj.110977 PMC3414599

[R86] Guo S, and Fraser MW. 2014. Propensity Score Analysis: Statistical Methods and Applications. Thousand Oaks, CA: SAGE Publications.

[R87] Hartman E, and Huang M. 2024. “Sensitivity Analysis for Survey Weights.” Political Analysis 32: 1–16.

[R88] Hastie T, Tibshirani R, and Friedman J. 2009. Elements of Statistical Learning: Data Mining, Inference, and Prediction. New York, NY: Springer.

[R89] Hastings WK 1970. “Monte Carlo Sampling Methods Using Markov Chains and their Applications.” Biometrika 57: 97–109.

[R90] Haziza D, and Beaumont JF. 2017. “Construction of Weights in Surveys: A Review.” Statistical Science 32: 206–226.

[R91] He Y 2010. “Missing Data Analysis Using Multiple Imputation: Getting to the Heart of the Matter.” Circulation. Cardiovascular Quality and Outcomes 3: 98–105. 20123676 10.1161/CIRCOUTCOMES.109.875658 PMC2818781

[R92] Heckman JJ 1976. “The Common Structure of Statistical Models of Truncation, Sample Selection and Limited Dependent Variables and a Simple Estimator for Such Models.” Annals of Economic and Social Measurement 5(4): 475–492.

[R93] Heckman JJ 1979. “Sample Selection Bias as a Specification Error.” Econometrica 47: 153–161. 10.2307/1912352.

[R94] Heckman JJ, and Pinto R. 2022. “The Econometric Model for Causal Policy Analysis.” Annual Review of Economics 14: 893–923. 10.1146/annurev-economics-051520-015456 PMC9894266 36741238

[R95] Heckman JJ, Urzua S, and Vytlacil E. 2006. “Understanding Instrumental Variables in Models with Essential Heterogeneity.” The Review of Economics and Statistics 88: 389–432.

[R96] Heeringa SG, West BT, and Berglund PA. 2017. Applied Survey Data Analysis. Boca Raton, FL: Chapman and Hall/CRC.

[R97] Heitjan DF, and Basu S. 1996. “Distinguishing “Missing at Random” and “Missing Completely at Random”.” The American Statistician 50: 207–213.

[R98] Helsel DR 2011. Statistics for Censored Environmental Data Using Minitab and R. Hoboken, NJ: John Wiley & Sons.

[R99] Herlihy AT, Kentula ME, Magee TK, Lomnicky GA, Nahlik AM, and Serenbetz G. 2019. “Striving for Consistency in the National Wetland Condition Assessment: Developing a Reference Condition Approach for Assessing Wetlands at a Continental Scale.” Environmental Monitoring and Assessment 191: 1–20. 10.1007/s10661-019-7325-3 PMC6586693 31222681

[R100] Higham M, Ver Hoef J, Madsen L, and Aderman A. 2021. “Adjusting a Finite Population Block Kriging Estimator for Imperfect Detection.” Environmetrics 32: e2654.

[R101] Hobbs NT, and Hooten MB. 2015. Bayesian Models: A Statistical Primer for Ecologists. Princeton, NJ: Princeton University Press.

[R102] Holland PW 1986. “Statistics and Causal Inference.” Journal of the American Statistical Association 81: 945–960.

[R103] Hooten MB, and Hobbs NT. 2015. “A Guide to Bayesian Model Selection for Ecologists.” Ecological Monographs 85: 3–28.

[R104] Horvitz DG, and Thompson DJ. 1952. “A Generalization of Sampling without Replacement from a Finite Universe.” Journal of the American Statistical Association 47: 663–685.

[R105] Hossie TJ, Gobin J, and Murray DL. 2021. “Confronting Missing Ecological Data in the Age of Pandemic Lockdown.” Frontiers in Ecology and Evolution 9: 669477.

[R106] Hudgens MG, and Halloran ME. 2006. “Causal Vaccine Effects on Binary Postinfection Outcomes.” Journal of the American Statistical Association 101: 51–64. 19096723 10.1198/016214505000000970 PMC2603579

[R107] Hughes RA, Heron J, Sterne JA, and Tilling K. 2019. “Accounting for Missing Data in Statistical Analyses: Multiple Imputation Is Not Always the Answer.” International Journal of Epidemiology 48: 1294–1304. 30879056 10.1093/ije/dyz032 PMC6693809

[R108] Imbens GW 2020. “Potential Outcome and Directed Acyclic Graph Approaches to Causality: Relevance for Empirical Practice in Economics.” Journal of Economic Literature 58: 1129–79.

[R109] Jaynes ET, and Kempthorne O. 1976. “Confidence Intervals Vs Bayesian Intervals.” In Foundations of Probability Theory, Statistical Inference, and Statistical Theories of Science: Proceedings of an International Research Colloquium Held at the University of Western Ontario, London, Canada, 10–13 May 1973 Volume II Foundations and Philosophy of Statistical Inference, eds. Harper WL and Hooker CA, 175–257. Dordrecht: Springer Netherlands. 10.1007/978-94-010-1436-6_6.

[R110] Johnson AA, Ott MQ, and Dogucu M. 2022. Bayes Rules!: An Introduction to Applied Bayesian Modeling. Boca Raton, FL: Chapman and Hall/CRC.

[R111] Johnson TF, Isaac NJ, Paviolo A, and González-Suárez M. 2021. “Handling Missing Values in Trait Data.” Global Ecology and Biogeography 30: 51–62.

[R112] Jones MP 1996. “Indicator and Stratification Methods for Missing Explanatory Variables in Multiple Linear Regression.” Journal of the American Statistical Association 91: 222–230.

[R113] Kalton G, and Kasprzyk D. 1986. “The Treatment of Missing Survey Data.” Survey Methodology 12: 1–16.

[R114] Kang H 2013. “The Prevention and Handling of the Missing Data.” Korean Journal of Anesthesiology 64: 402–6. 23741561 10.4097/kjae.2013.64.5.402 PMC3668100

[R115] Kang JD, and Schafer JL. 2007. “Demystifying Double Robustness: A Comparison of Alternative Strategies for Estimating a Population Mean from Incomplete Data.” Statistical Science 22: 523–539. 10.1214/07-STS227 PMC2397555 18516239

[R116] Kass RE, and Raftery AE. 1995. “Bayes Factors.” Journal of the American Statistical Association 90: 773–795.

[R117] Kellner KF, and Swihart RK. 2014. “Accounting for Imperfect Detection in Ecology: A Quantitative Review.” PLoS One 9: e111436. 25356904 10.1371/journal.pone.0111436 PMC4214722

[R118] Kentula ME, and Paulsen SG. 2019. “The 2011 National Wetland Condition Assessment: Overview and an Invitation.” Environmental Monitoring and Assessment 191: 325. 31222397 10.1007/s10661-019-7316-4 PMC6586703

[R119] Kenward MG, and Carpenter J. 2007. “Multiple Imputation: Current Perspectives.” Statistical Methods in Medical Research 16: 199–218. 17621468 10.1177/0962280206075304

[R120] Kim JK, and Haziza D. 2014. “Doubly Robust Inference with Missing Data in Survey Sampling.” Statistica Sinica 24: 375–394.

[R121] Kim SW, Blomberg SP, and Pandolfi JM. 2018. “Transcending Data Gaps: A Framework to Reduce Inferential Errors in Ecological Analyses.” Ecology Letters 21: 1200–1210. 29797760 10.1111/ele.13089

[R122] Kleinke K 2017. “Multiple Imputation under Violated Distributional Assumptions: A Systematic Evaluation of the Assumed Robustness of Predictive Mean Matching.” Journal of Educational and Behavioral Statistics 42: 371–404.

[R123] Kline RB 2023. Principles and Practice of Structural Equation Modeling. New York, NY: Guilford Publications.

[R124] Knol MJ, Janssen KJ, Donders ART, Egberts AC, Heerdink ER, Grobbee DE, Moons KG, and Geerlings MI. 2010. “Unpredictable Bias when Using the Missing Indicator Method or Complete Case Analysis for Missing Confounder Values: An Empirical Example.” Journal of Clinical Epidemiology 63: 728–736. 20346625 10.1016/j.jclinepi.2009.08.028

[R125] Kontopantelis E, White IR, Sperrin M, and Buchan I. 2017. “Outcome-Sensitive Multiple Imputation: A Simulation Study.” BMC Medical Research Methodology 17: 1–13. 28068910 10.1186/s12874-016-0281-5 PMC5220613

[R126] Kott PS 2012. “Why one Should Incorporate the Design Weights when Adjusting for Unit Nonresponse Using Response Homogeneity Groups.” Survey Methodology 38: 95–99.

[R127] Kromrey JD, and Hines CV. 1994. “Nonrandomly Missing Data in Multiple Regression: An Empirical Comparison of Common Missing-Data Treatments.” Educational and Psychological Measurement 54: 573–593.

[R128] Kruskal W, and Mosteller F. 1979. “Representative Sampling, III: The Current Statistical Literature.” International Statistical Review 47, no. 3: 245–265.

[R129] Laaksonen S 2018. Survey Methodology and Missing Data. Survey Methodology and Missing Data: Tools and Techniques for Practitioners. Berlin: Springer International Publishing.

[R130] Lin JY, Lu Y, and Tu X. 2012. “How to Avoid Missing Data and the Problems they Pose: Design Considerations.” Shanghai Archives of Psychiatry 24: 181. 25324625 10.3969/j.issn.1002-0829.2012.03.010 PMC4198852

[R131] Lipsitz S, Parzen M, and Zhao LP. 2002. “A Degrees-of-Freedom Approximation in Multiple Imputation.” Journal of Statistical Computation and Simulation 72: 309–318.

[R132] Little RJ 1986. “Survey Nonresponse Adjustments for Estimates of Means.” International Statistical Review 54, no. 2: 139–157.

[R133] Little RJ 1988a. “Missing Data Adjustments in Large Surveys.” Journal of Business & Economic Statistics 6: 287–296.

[R134] Little RJ 1988b. “A Test of Missing Completely at Random for Multivariate Data with Missing Values.” Journal of the American Statistical Association 83: 1198–1202.

[R135] Little RJ 1992. “Regression with Missing X’s: A Review.” Journal of the American Statistical Association 87: 1227–37.

[R136] Little RJ 2006. “Calibrated Bayes: A Bayes/Frequentist Roadmap.” The American Statistician 60: 213–223. 10.1198/000313006X117837.

[R137] Little RJ 2021. “Missing Data Assumptions.” Annual Review of Statistics and Its Application 8: 89–107. 10.1146/annurev-statistics-040720-031104.

[R138] Little RJ, Carpenter JR, and Lee KJ. 2024. “A Comparison of Three Popular Methods for Handling Missing Data: Complete-Case Analysis, Inverse Probability Weighting, and Multiple Imputation.” Sociological Methods & Research 53: 1105–35.

[R139] Little RJ, D’agostino R, Cohen ML, Dickersin K, Emerson SS, Farrar JT, Frangakis C, 2012. “The Prevention and Treatment of Missing Data in Clinical Trials.” New England Journal of Medicine 367: 1355–60. 23034025 10.1056/NEJMsr1203730 PMC3771340

[R140] Little RJ, and Rubin DB. 1989. “The Analysis of Social Science Data with Missing Values.” Sociological Methods & Research 18: 292–326.

[R141] Little RJ, and Rubin DB. 2019. Statistical Analysis with Missing Data, 3rd ed. Hoboken, NJ: John Wiley & Sons.

[R142] Little RJ, and Vartivarian S. 2005. “Does Weighting for Nonresponse Increase the Variance of Survey Means?” Survey Methodology 31: 161.

[R143] Little RJA 1994. “A Class of Pattern-Mixture Models for Normal Incomplete Data.” Biometrika 81: 471–483. 10.2307/2337120.

[R144] Lohr SL 2021. Sampling: Design and Analysis. Boca Raton, FL: CRC Press.

[R145] Łopucki R, Kiersztyn A, Pitucha G, and Kitowski I. 2022. “Handling Missing Data in Ecological Studies: Ignoring Gaps in the Dataset Can Distort the Inference.” Ecological Modelling 468: 109964.

[R146] Lumley T 2011. Complex Surveys: A Guide to Analysis Using R. Hoboken, NJ: John Wiley & Sons.

[R147] MacKenzie DI 2005. “What Are the Issues with Presence-Absence Data for Wildlife Managers?” The Journal of Wildlife Management 69: 849–860.

[R148] Magee TK, Blocksom KA, and Fennessy MS. 2019. “A National-Scale Vegetation Multimetric Index (VMMI) as an Indicator of Wetland Condition across the Conterminous United States.” Environmental Monitoring and Assessment 191: 322. 31222469 10.1007/s10661-019-7324-4 PMC6586711

[R149] Marshall A, Altman DG, Royston P, and Holder RL. 2010. “Comparison of Techniques for Handling Missing Covariate Data within Prognostic Modelling Studies: A Simulation Study.” BMC Medical Research Methodology 10: 1–16. 20085642 10.1186/1471-2288-10-7 PMC2824146

[R150] Mc Overton J, Young T, and Overton W. 1993. “Using Found Data to Augment a Probability Sample: Procedure and Case Study.” Environmental Monitoring and Assessment 26: 65–83. 24225898 10.1007/BF00555062

[R151] McCauley D, Arnold W, Saxton J, and Turner C. 2019. “Applying Adaptive Management and Lessons Learned from National Assessments to Address Logistical Challenges in the National Wetland Condition Assessment.” Environmental Monitoring and Assessment 191: 329. 31222449 10.1007/s10661-019-7320-8 PMC6586718

[R152] McConville KS, Moisen GG, and Frescino TS. 2020. “A Tutorial on Model-Assisted Estimation with Application to Forest Inventory.” Forests 11: 244.

[R153] McElreath R 2018. Statistical Rethinking: A Bayesian Course with Examples in R and Stan. Boca Raton, FL: Chapman and Hall/CRC.

[R154] McLachlan GJ, and Krishnan T. 2007. The EM Algorithm and Extensions. Hoboken, NJ: John Wiley & Sons.

[R155] Mealli F, and Rubin DB. 2015. “Clarifying Missing at Random and Related Definitions, and Implications when Coupled with Exchangeability.” Biometrika 102: 995–1000. 10.1093/biomet/asv035.

[R156] Meng XL 1994. “Multiple-Imputation Inferences with Uncongenial Sources of Input.” Statistical Science 9, no. 4: 538–558.

[R157] Meng XL 2022. “Comments on ‘Statistical Inference with Non-Probability Survey Samples’ – miniaturizing Data Defect Correlation: A Versatile Strategy for Handling Non-probability Samples.” Survey Methodology 48, no. 2: 339–360.

[R158] Metropolis N, Rosenbluth AW, Rosenbluth MN, Teller AH, and Teller E. 1953. “Equation of State Calculations by Fast Computing Machines.” The Journal of Chemical Physics 21: 1087–92. 10.1063/5.0309018 41342507

[R159] Miles A 2016. “Obtaining Predictions from Models Fit to Multiply Imputed Data.” Sociological Methods & Research 45: 175–185.

[R160] Miller RG 1974. “The Jackknife—A Review.” Biometrika 61: 1–15.

[R161] Mitsch WJ, Gosselink JG, Anderson CJ, and Fennessy MS. 2023. Wetlands, 6th ed. Hoboken, NJ: John Wiley & Sons.

[R162] Mohan K, and Pearl J. 2021. “Graphical Models for Processing Missing Data.” Journal of the American Statistical Association 116: 1023–37.

[R163] Mohan K, Pearl J, and Tian J. 2013. “Graphical Models for Inference with Missing Data.” Advances in Neural Information Processing Systems 1: 1277–85.

[R164] Moher D, Hopewell S, Schulz KF, Montori V, Gøtzsche PC, Devereaux PJ, Elbourne D, Egger M, and Altman DG. 2010. “Consort 2010 Explanation and Elaboration: Updated Guidelines for Reporting Parallel Group Randomised Trials.” BMJ 340: c869. 20332511 10.1136/bmj.c869 PMC2844943

[R165] Molenberghs G, Fitzmaurice G, Kenward MG, Tsiatis A, and Verbeke G. 2014. Handbook of Missing Data Methodology. Boca Raton, FL: CRC Press.

[R166] Montgomery DC, Peck EA, and Vining GG. 2021. Introduction to Linear Regression Analysis. Hoboken, NJ: John Wiley & Sons.

[R167] Moons KG, Donders RA, Stijnen T, and Harrell FE Jr. 2006. “Using the Outcome for Imputation of Missing Predictor Values Was Preferred.” Journal of Clinical Epidemiology 59: 1092–1101. 16980150 10.1016/j.jclinepi.2006.01.009

[R168] Moreno-Betancur M, Lee KJ, Leacy FP, White IR, Simpson JA, and Carlin JB. 2018. “Canonical Causal Diagrams to Guide the Treatment of Missing Data in Epidemiologic Studies.” American Journal of Epidemiology 187: 2705–15. 30124749 10.1093/aje/kwy173 PMC6269242

[R169] Morgan SL, and Winship C. 2014. Counterfactuals and Causal Inference: Methods and Principles for Social Research. Cambridge: Cambridge University Press.

[R170] Morris TP, White IR, and Royston P. 2014. “Tuning Multiple Imputation by Predictive Mean Matching and Local Residual Draws.” BMC Medical Research Methodology 14: 1–13. 24903709 10.1186/1471-2288-14-75 PMC4051964

[R171] Murray JS 2018. “Multiple Imputation: A Review of Practical and Theoretical Findings.” Statistical Science 33: 142–159.

[R172] Myers TA 2011. “Goodbye, Listwise Deletion: Presenting Hot Deck Imputation as an Easy and Effective Tool for Handling Missing Data.” Communication Methods and Measures 5: 297–310.

[R173] Nakagawa S 2015. “Missing Data: Mechanisms, Methods and Messages.” In Ecological Statistics: Contemporary Theory and Application, edited by Fox GA, Negrete-Yankelevich S, Sosa VJ, 81–105. Oxford: Oxford University Press.

[R174] Nakagawa S, and Freckleton RP. 2008. “Missing Inaction: The Dangers of Ignoring Missing Data.” Trends in Ecology & Evolution 23: 592–96. 18823677 10.1016/j.tree.2008.06.014

[R175] Nakagawa S, and Freckleton RP. 2011. “Model Averaging, Missing Data and Multiple Imputation: A Case Study for Behavioural Ecology.” Behavioral Ecology and Sociobiology 65: 103–116.

[R176] National Academies. 2019. Reproducibility and Replicability in Science. Washington, DC: The National Academies Press. 10.17226/25303. 31596559

[R177] Newey WK, Powell JL, and Walker JR. 1990. “Semiparametric Estimation of Selection Models: Some Empirical Results.” The American Economic Review 80: 324–28.

[R178] Newman DA 2014. “Missing data: Five practical guidelines.” Organizational Research Methods 17: 372–411.

[R179] Nguyen CD, Carlin JB, and Lee KJ. 2017. “Model Checking in Multiple Imputation: An Overview and Case Study.” Emerging Themes in Epidemiology 14: 1–12. 28852415 10.1186/s12982-017-0062-6 PMC5569512

[R180] Noble DW, and Nakagawa S. 2021. “Planned Missing Data Designs and Methods: Options for Strengthening Inference, Increasing Research Efficiency and Improving Animal Welfare in Ecological and Evolutionary Research.” Evolutionary Applications 14: 1958–68. 34429741 10.1111/eva.13273 PMC8372070

[R181] Olinsky A, Chen S, and Harlow L. 2003. “The Comparative Efficacy of Imputation Methods for Missing Data in Structural Equation Modeling.” European Journal of Operational Research 151: 53–79.

[R182] Olsen AR, Kincaid TM, Kentula ME, and Weber MH. 2019. “Survey Design to Assess Condition of Wetlands in the United States.” Environmental Monitoring and Assessment 191: 268. 31222669 10.1007/s10661-019-7322-6 PMC6586691

[R183] O’neill R, and Temple R. 2012. “The Prevention and Treatment of Missing Data in Clinical Trials: An FDA Perspective on the Importance of Dealing with it.” Clinical Pharmacology and Therapeutics 91: 550–54. 22318615 10.1038/clpt.2011.340

[R184] Pearl J 2009a. “Causal Inference in Statistics: An Overview.” Statistics Surveys 3: 96–146.

[R185] Pearl J 2009b. Causality. Cambridge: Cambridge University Press.

[R186] Pearson RK 2006. “The Problem of Disguised Missing Data.” ACM SIGKDD Explorations Newsletter 8: 83–92.

[R187] Pedersen AB, Mikkelsen EM, Cronin-Fenton D, Kristensen NR, Pham TM, Pedersen L, and Petersen I. 2017. “Missing Data and Multiple Imputation in Clinical Epidemiological Research.” Clinical Epidemiology 9: 157–166. 28352203 10.2147/CLEP.S129785 PMC5358992

[R188] Penone C, Davidson AD, Shoemaker KT, Di Marco M, Rondinini C, Brooks TM, Young BE, Graham CH, and Costa GC. 2014. “Imputation of Missing Data in Life-History Trait Datasets: Which Approach Performs the Best?” Methods in Ecology and Evolution 5: 961–970.

[R189] Perkins NJ, Cole SR, Harel O, Tchetgen Tchetgen EJ, Sun B, Mitchell EM, and Schisterman EF. 2018. “Principled Approaches to Missing Data in Epidemiologic Studies.” American Journal of Epidemiology 187: 568–575. 29165572 10.1093/aje/kwx348 PMC5860376

[R190] Peterson EE, and Ver Hoef JM. 2010. “A Mixed-Model Moving-Average Approach to Geostatistical Modeling in Stream Networks.” Ecology 91: 644–651. 20426324 10.1890/08-1668.1

[R191] Piccininni M, Konigorski S, Rohmann JL, and Kurth T. 2020. “Directed Acyclic Graphs and Causal Thinking in Clinical Risk Prediction Modeling.” BMC Medical Research Methodology 20: 179. 32615926 10.1186/s12874-020-01058-z PMC7331263

[R192] R Core Team. 2025. R: A Language and Environment for Statistical Computing. Vienna: R Foundation for Statistical Computing.

[R193] Rancourt E, Särndal C, and Lee H. 1994. “Estimation of the Variance in the Presence of Nearest Neighbor Imputation.” In Proceedings of the Section on Survey Research Methods, 888–893. American Statistical Association.

[R194] Reiter JP 2007. “Small-Sample Degrees of Freedom for Multi-Component Significance Tests with Multiple Imputation for Missing Data.” Biometrika 94: 502–8.

[R195] Reiter JP, Raghunathan TE, and Kinney SK. 2006. “The Importance of Modeling the Sampling Design in Multiple Imputation for Missing Data.” Survey Methodology 32: 143.

[R196] Robert CP, Casella G, and Casella G. 1999. Monte Carlo Statistical Methods. New York, NY: Springer.

[R197] Robins JM 2000. “Robust Estimation in Sequentially Ignorable Missing Data and Causal Inference Models.” In Proceedings of the American Statistical Association, Indianapolis, IN, 6–10.

[R198] Robins JM, Rotnitzky A, and Zhao LP. 1994. “Estimation of Regression Coefficients when some Regressors Are Not Always Observed.” Journal of the American Statistical Association 89: 846–866.

[R199] Robins JM, and Wang N. 2000. “Inference for Imputation Estimators.” Biometrika 87: 113–124.

[R200] Rosenbaum PR, and Rubin DB. 1983. “The Central Role of the Propensity Score in Observational Studies for Causal Effects.” Biometrika 70: 41–55.

[R201] Roth PL 1994. “Missing Data: A Conceptual Review for Applied Psychologists.” Personnel Psychology 47: 537–560.

[R202] Rubin DB 1976. “Inference and Missing Data.” Biometrika 63: 581–592.

[R203] Rubin DB 1977. “Formalizing Subjective Notions about the Effect of Nonrespondents in Sample Surveys.” Journal of the American Statistical Association 72: 538–543.

[R204] Rubin DB 1986. “Statistical Matching Using File Concatenation with Adjusted Weights and Multiple Imputations.” Journal of Business & Economic Statistics 4: 87–94.

[R205] Rubin DB 1996. “Multiple Imputation after 18+ Years.” Journal of the American Statistical Association 91: 473–489.

[R206] Rubin DB 2001. “Using Propensity Scores to Help Design Observational Studies: Application to the Tobacco Litigation.” Health Services and Outcomes Research Methodology 2: 169–188.

[R207] Rubin DB 2004. Multiple Imputation for Nonresponse in Surveys. Hoboken, NJ: John Wiley & Sons.

[R208] Rubin DB 2009. “Should Observational Studies be Designed to Allow Lack of Balance in Covariate Distributions across Treatment Groups?” Statistics in Medicine 28: 1420–23.

[R209] Särndal CE, Swensson B, and Wretman J. 2003. Model Assisted Survey Sampling. New York, NY: Springer Science & Business Media.

[R210] Särndal CE, Swensson B, and Wretman JH. 1989. “The Weighted Residual Technique for Estimating the Variance of the General Regression Estimator of the Finite Population Total.” Biometrika 76: 527–537.

[R211] Särndal CE, Thomsen I, Hoem JM, Lindley D, Barndorff-Nielsen O, and Dalenius T. 1978. “Design-Based and Model-Based Inference in Survey Sampling [with Discussion and Reply].” Scandinavian Journal of Statistics 5, no. 1: 27–52.

[R212] Saunders JA, Morrow-Howell N, Spitznagel E, Doré P, Proctor EK, and Pescarino R. 2006. “Imputing Missing Data: A Comparison of Methods for Social Work Researchers.” Social Work Research 30: 19–31.

[R213] Schafer JL 1997. Analysis of Incomplete Multivariate Data. Boca Raton, FL: CRC Press.

[R214] Schafer JL, and Graham JW. 2002. “Missing Data: Our View of the State of the Art.” Psychological Methods 7: 147–177. 12090408

[R215] Schafer JL, and Olsen MK. 1998. “Multiple Imputation for Multivariate Missing-Data Problems: A Data Analyst’s Perspective.” Multivariate Behavioral Research 33: 545–571. 26753828 10.1207/s15327906mbr3304_5

[R216] Scharf H, Hooten MB, and Johnson DS. 2017. “Imputation Approaches for Animal Movement Modeling.” Journal of Agricultural, Biological and Environmental Statistics 22: 335–352.

[R217] Schenker N, and Taylor JM. 1996. “Partially Parametric Techniques for Multiple Imputation.” Computational Statistics & Data Analysis 22: 425–446.

[R218] Scheuren F 2005. “Multiple Imputation: How it Began and Continues.” The American Statistician 59: 315–19.

[R219] Schlomer GL, Bauman S, and Card NA. 2010. “Best Practices for Missing Data Management in Counseling Psychology.” Journal of Counseling Psychology 57: 1–10. 21133556 10.1037/a0018082

[R220] Schoemann AM, Moore EWG, and Yagiz G. 2024. “How and why to Follow Best Practices for Testing Mediation Models with Missing Data.” International Journal of Psychology 60, no. 1: e13257. 39420243 10.1002/ijop.13257 PMC11625877

[R221] Schomaker M, and Heumann C. 2018. “Bootstrap Inference when Using Multiple Imputation.” Statistics in Medicine 37: 2252–66. 29682776 10.1002/sim.7654 PMC5986623

[R222] Schwarz G 1978. “Estimating the Dimension of a Model.” The Annals of Statistics 6, no. 2: 461–64.

[R223] Seaman S, Galati J, Jackson D, and Carlin J. 2013. “What Is Meant by “Missing at Random”?” Statistical Science 28: 257–268.

[R224] Seaman S, and White I. 2014. “Inverse Probability Weighting with Missing Predictors of Treatment Assignment or Missingness.” Communications in Statistics - Theory and Methods 43: 3499–3515.

[R225] Seaman SR, and Vansteelandt S. 2018. “Introduction to Double Robust Methods for Incomplete Data.” Statistical Science: A Review Journal of the Institute of Mathematical Statistics 33: 184. 29731541 10.1214/18-STS647 PMC5935236

[R226] Seaman SR, and White IR. 2013. “Review of Inverse Probability Weighting for Dealing with Missing Data.” Statistical Methods in Medical Research 22: 278–295. 21220355 10.1177/0962280210395740

[R227] Seaman SR, White IR, Copas AJ, and Li L. 2012. “Combining Multiple Imputation and Inverse-Probability Weighting.” Biometrics 68: 129–137. 22050039 10.1111/j.1541-0420.2011.01666.x PMC3412287

[R228] Shah AD, Bartlett JW, Carpenter J, Nicholas O, and Hemingway H. 2014. “Comparison of Random Forest and Parametric Imputation Models for Imputing Missing Data Using MICE: A CALIBER Study.” American Journal of Epidemiology 179: 764–774. 24589914 10.1093/aje/kwt312 PMC3939843

[R229] Siddique J, and Belin TR. 2008. “Multiple Imputation Using an Iterative Hot-Deck with Distance-Based Donor Selection.” Statistics in Medicine 27: 83–102. 17634973 10.1002/sim.3001

[R230] Srebotnjak T, Carr G, de Sherbinin A, and Rickwood C. 2012. “A Global Water Quality Index and Hot-Deck Imputation of Missing Data.” Ecological Indicators 17: 108–119.

[R231] Steen VA, Tingley MW, Paton PW, and Elphick CS. 2020. “Spatial Thinning and Class Balancing: Key Choices Lead to Variation in the Performance of Species Distribution Models with Citizen Science Data.” Methods in Ecology and Evolution 12: 216–226.

[R232] Stevens DL Jr., and Olsen AR. 2004. “Spatially Balanced Sampling of Natural Resources.” Journal of the American Statistical Association 99: 262–278.

[R233] Su YS, Gelman A, Hill J, and Yajima M. 2011. “Multiple Imputation with Diagnostics (mi) in R: Opening Windows into the Black Box.” Journal of Statistical Software 45: 1–31.

[R234] Sullivan TR, White IR, Salter AB, Ryan P, and Lee KJ. 2018. “Should Multiple Imputation be the Method of Choice for Handling Missing Data in Randomized Trials?” Statistical Methods in Medical Research 27: 2610–26. 28034175 10.1177/0962280216683570 PMC5393436

[R235] Tanner MA, and Wong WH. 1987. “The Calculation of Posterior Distributions by Data Augmentation.” Journal of the American Statistical Association 82: 528–540.

[R236] Taugourdeau S, Villerd J, Plantureux S, Huguenin-Elie O, and Amiaud B. 2014. “Filling the Gap in Functional Trait Databases: Use of Ecological Hypotheses to Replace Missing Data.” Ecology and Evolution 4: 944–958. 24772273 10.1002/ece3.989 PMC3997312

[R237] Tchetgen Tchetgen EJ 2014. “Identification and Estimation of Survivor Average Causal Effects.” Statistics in Medicine 33: 3601–28. 24889022 10.1002/sim.6181 PMC4131726

[R238] Ter Braak C, Van Strien A, Meijer R, and Verstrael T. 1994. “Analysis of Monitoring Data with Many Missing Values: Which Method?” In Bird Numbers 1992. Distribution, Monitoring and Ecological Aspects: Proceedings 12th International Conference of IBCC and EOAC, Noordwijkerhout, The Netherlands, 663–673.

[R239] Terando AJ, Youngsteadt E, Meineke EK, and Prado SG. 2017. “Ad Hoc Instrumentation Methods in Ecological Studies Produce Highly Biased Temperature Measurements.” Ecology and Evolution 7: 9890–9904. 29238523 10.1002/ece3.3499 PMC5723608

[R240] Thoemmes F, and Mohan K. 2015. “Graphical Representation of Missing Data Problems.” Structural Equation Modeling: A Multidisciplinary Journal 22: 631–642.

[R241] Thoemmes F, and Rose N. 2014. “A Cautious Note on Auxiliary Variables that Can Increase Bias in Missing Data Problems.” Multivariate Behavioral Research 49: 443–459. 26732358 10.1080/00273171.2014.931799

[R242] Tierney L 1994. “Markov Chains for Exploring Posterior Distributions.” The Annals of Statistics 22, no. 4: 1701–28.

[R243] Tilling K, Williamson EJ, Spratt M, Sterne JA, and Carpenter JR. 2016. “Appropriate Inclusion of Interactions Was Needed to Avoid Bias in Multiple Imputation.” Journal of Clinical Epidemiology 80: 107–115. 27445178 10.1016/j.jclinepi.2016.07.004 PMC5176003

[R244] Tobler WR 1970. “A Computer Movie Simulating Urban Growth in the Detroit Region.” Economic Geography 46: 234–240.

[R245] Tredennick AT, Hooker G, Ellner SP, and Adler PB. 2021. “A Practical Guide to Selecting Models for Exploration, Inference, and Prediction in Ecology.” Ecology 102: e03336. 33710619 10.1002/ecy.3336 PMC8187274

[R246] USDA-NRCS. 2020. “The Plants Database.” http://plants.usda.gov.

[R247] USEPA. 2015. National Wetland Condition Assessment 2016: Laboratory Operations Manual. Washington, DC: U.S. Environmental Protection Agency.

[R248] USEPA. 2016. National Wetland Condition Assessment 2016: Field Operations Manual. Washington, DC: U.S. Environmental Protection Agency. https://www.epa.gov/sites/default/files/2017-08/documents/nwca2016_fom_v1_1a_full_0.pdf.

[R249] USEPA. 2023. National Wetland Condition Assessment: 2016 Technical Support Document. Washington, DC: U.S. Environmental Protection Agency. https://www.epa.gov/system/files/documents/2023-04/NWCA%202016%20Technical%20Support%20Document_20230216.pdf.

[R250] Vach W 2012. Logistic Regression with Missing Values in the Covariates. New York, NY: Springer Science & Business Media.

[R251] Valliant R, Dorfman AH, and Royall RM. 2000. Finite Population Sampling and Inference: A Prediction Approach. New York, NY: Wiley.

[R252] Van Buuren S 2018. Flexible Imputation of Missing Data. Boca Raton, FL: CRC Press.

[R253] Van Buuren S, Brand JP, Groothuis-Oudshoorn CG, and Rubin DB. 2006. “Fully Conditional Specification in Multivariate Imputation.” Journal of Statistical Computation and Simulation 76: 1049–64.

[R254] Van Buuren S, and Groothuis-Oudshoorn K. 2011. “mice: Multivariate Imputation by Chained Equations in R.” Journal of Statistical Software 45: 1–67.

[R255] Van der Heijden GJ, Donders ART, Stijnen T, and Moons KG. 2006. “Imputation of Missing Values Is Superior to Complete Case Analysis and the Missing-Indicator Method in Multivariable Diagnostic Research: A Clinical Example.” Journal of Clinical Epidemiology 59: 1102–9. 16980151 10.1016/j.jclinepi.2006.01.015

[R256] Van Deusen PC 1997. “Annual Forest Inventory Statistical Concepts with Emphasis on Multiple Imputation.” Canadian Journal of Forest Research 27: 379–384.

[R257] Ver Hoef JM 2008. “Spatial Methods for Plot-Based Sampling of Wildlife Populations.” Environmental and Ecological Statistics 15: 3–13.

[R258] Ver Hoef JM, and Peterson EE. 2010. “A Moving Average Approach for Spatial Statistical Models of Stream Networks.” Journal of the American Statistical Association 105: 6–18.

[R259] Ver Hoef JM, and Temesgen H. 2013. “A Comparison of the Spatial Linear Model to Nearest Neighbor (k-NN) Methods for Forestry Applications.” PLoS One 8: e59129. 23527110 10.1371/journal.pone.0059129 PMC3602606

[R260] Vink G, Frank LE, Pannekoek J, and Van Buuren S. 2014. “Predictive Mean Matching Imputation of Semicontinuous Variables.” Statistica Neerlandica 68: 61–90.

[R261] Von Hippel PT 2009. “How to Impute Interactions, Squares, and Other Transformed Variables.” Sociological Methodology 39: 265–291.

[R262] Wagstaff DA, and Harel O. 2011. “A Closer Examination of Three Small-Sample Approximations to the Multiple-Imputation Degrees of Freedom.” The Stata Journal 11: 403–419.

[R263] Wang L, Zhou XH, and Richardson TS. 2017. “Identification and Estimation of Causal Effects with Outcomes Truncated by Death.” Biometrika 104: 597–612. 29430035 10.1093/biomet/asx034 PMC5793679

[R264] Wasserstein RL, and Lazar NA. 2016. “The ASA Statement on p-Values: context, Process, and Purpose.” The American Statistician 70: 129–133.

[R265] White IR, Royston P, and Wood AM. 2011. “Multiple Imputation Using Chained Equations: Issues and Guidance for Practice.” Statistics in Medicine 30: 377–399. 21225900 10.1002/sim.4067

[R266] White IR, and Thompson SG. 2005. “Adjusting for Partially Missing Baseline Measurements in Randomized Trials.” Statistics in Medicine 24: 993–1007. 15570623 10.1002/sim.1981

[R267] Wilen BO, and Bates M. 1995. “The US Fish and Wildlife Service’s National Wetlands Inventory Project.” In Classification and Inventory of the World’s Wetlands, edited by Finlayson CM, van der Valk AG, 153–169. New York, NY: Springer.

[R268] Wilks SS 1932. “Moments and Distributions of Estimates of Population Parameters from Fragmentary Samples.” The Annals of Mathematical Statistics 3: 163–195.

[R269] Wood AM, White IR, and Thompson SG. 2004. “Are Missing Outcome Data Adequately Handled? A Review of Published Randomized Controlled Trials in Major Medical Journals.” Clinical Trials 1: 368–376. 16279275 10.1191/1740774504cn032oa

[R270] Wu C 2022. “Statistical Inference with Non-probability Survey Samples.” Survey Methodology 48: 283–311.

[R271] Yuan YC 2010. Multiple Imputation for Missing Data: Concepts and New Development (Version 9.0). Rockville, MD: SAS Institute Inc.

[R272] Zhao Y, and Long Q. 2016. “Multiple Imputation in the Presence of High-Dimensional Data.” Statistical Methods in Medical Research 25: 2021–35. 24275026 10.1177/0962280213511027

[R273] Zimmerman DL, and Ver Hoef JM. 2024. Spatial Linear Models for Environmental Data. Boca Raton, FL: CRC Press.

