# Supplementary material for: Missing data in ecology: Syntheses, clarifications, and considerations
Source: Ecol Monogr. Author manuscript; Available in PMC 2026 Nov 5. (PMC12973031; doi:10.1002/ecm.70037)
Supplement: Supplementary file 1 [file NIHMS2149586-supplement-Supplement1.pdf]

Appendix S1

Missing data in ecology: Syntheses, clarifications, and considerations

Michael Dumelle, Rob Trangucci, Amanda M. Nahlik, Anthony R. Olsen, Kathryn M. Irvine, Karen Blocksom, Jay M. Ver Hoef, Claudio Fuentes

Ecological Monographs

Section S1 Relationship between $\mathbf{Y}$, $\mathbf{y}$, $\mathbf{R}$, and $\mathbf{r}$

The matrices $\mathbf{Y}$, $\mathbf{R}$, $\mathbf{Y}_{obs}$, and $\mathbf{Y}_{mis}$ are very general and represent all possible observed items as well as missingness patterns. In practice, however, we only see one data set with a single set of observed items and a single missingness pattern. We indicate the values $\mathbf{Y}$, $\mathbf{R}$, $\mathbf{Y}_{obs}$, and $\mathbf{Y}_{mis}$ which are realized (i.e., seen) for a single data set as $\mathbf{y}$, $\mathbf{r}$, $\mathbf{y}_{obs}$, and $\mathbf{y}_{mis}$, respectively. To make these descriptions more concrete, we provide an additional example here that relies on familiarity with the Gaussian (i.e., normal) and Poisson distributions of random variables and some elementary `R` code for simulating these variables.

Suppose an analyst is studying two variables $\mathbf{Y}_1$ and $\mathbf{Y}_2$ for each of six units. Further suppose that each element of $\mathbf{Y}_1$ is an independent and identically distributed (i.e., i.i.d.) Gaussian random variable with a mean of zero and a variance of one, and that each element of $\mathbf{Y}_2$ is an i.i.d. Poisson random variable with a mean (i.e., rate) of two. The matrix $\mathbf{Y}$ has six rows (one for each unit) and two columns (one for each variable, $\mathbf{Y}_1$ and $\mathbf{Y}_2$), totaling 12 items. The matrix $\mathbf{Y}$ represents all possible realizations of these 12 items. A single realization of both $\mathbf{Y}_1$ and $\mathbf{Y}_2$ yield $\mathbf{y}_1$ and $\mathbf{y}_2$, respectively, which together compose $\mathbf{y}$. The relationship between $\mathbf{Y}$ and $\mathbf{y}$ is made more concrete via the following `R` example. We first create a function called `bold_y` that simulate a single realization of $\mathbf{y}_1$ and $\mathbf{y}_2$ and places them into the $\mathbf{y}$ matrix:

```r
bold_y <- function() {
  y_1 <- rnorm(n = 6, mean = 0, sd = 1)
  y_2 <- rpois(n = 6, lambda = 2)
  y <- matrix(c(y_1, y_2), nrow = 6, ncol = 2)
  return(y)
}
```

A single realization of $\mathbf{y}$ looks like:

```r
set.seed(1)
y_draw_one <- bold_y()
print(y_draw_one)
#>            [,1] [,2]
#> [1,] -0.6264538    3
#> [2,]  0.1836433    1
#> [3,] -0.8356286    3
#> [4,]  1.5952808    2
#> [5,]  0.3295078    3
#> [6,] -0.8204684    6
```

Another realization of $\mathbf{y}$ looks like:

```r
y_draw_two <- bold_y()
print(y_draw_two)
#>            [,1] [,2]
#> [1,] -0.3053884    2
#> [2,]  1.5117812    2
#> [3,]  0.3898432    2
#> [4,] -0.6212406    1
#> [5,] -2.2146999    3
```

```
#> [6,]  1.1249309    2
```

We can approximate the distribution of $\mathbf{Y}$ using the `approx_bold_Y` function, which simulates `s` different $\mathbf{y}$'s

```
approx_bold_Y <- function(s) {
  Y <- replicate(s, bold_y())
  return(Y)
}
```

The larger the `s`, the closer the approximation to $\mathbf{Y}$.

The following code generates 10,000 realizations of $\mathbf{y}$ and places them into an an array. The dimensions of the array are $6 \times 2 \times 10,000$:

```
set.seed(1)
approx_bold_Y_draw <- approx_bold_Y(s = 10000)
```

The first realization (6×2 array element) of $\mathbf{y}$ matches `y_draw_one`:

```
print(approx_bold_Y_draw[, , 1])
#>            [,1] [,2]
#> [1,] -0.6264538    3
#> [2,]  0.1836433    1
#> [3,] -0.8356286    3
#> [4,]  1.5952808    2
#> [5,]  0.3295078    3
#> [6,] -0.8204684    6
```

The 10,000th realization of of $\mathbf{y}$ is

```
print(approx_bold_Y_draw[, , 10000])
#>            [,1] [,2]
```

```
#> [1,]  1.2816568    3
#> [2,] -0.8675607    1
#> [3,]  0.4449571    2
#> [4,]  1.3997355    4
#> [5,] -0.2384143    2
#> [6,]  0.1294678    4
```

The first 40 realizations of $y_{1,1}$ are

```
head(approx_bold_Y_draw[1, 1, ], n = 40)
#>  [1] -0.62645381 -0.30538839  0.82122120 -1.47075238 -0.39428995 -0.70749516
#>  [7]  1.43302370  0.02800216  0.61072635 -0.13517862 -0.54252003 -0.47340064
#> [13]  0.38418536 -0.27911330 -0.07356440 -1.53644982 -1.11592011 -0.92936215
#> [19]  1.05848305  0.10580237 -1.23132342 -0.92610950  0.41197471  0.54132734
#> [25] -0.30874057  1.80314191 -0.05972328 -0.87870761 -0.06965481 -0.38923718
#> [31] -0.25893258  0.91980368  0.43370215  0.95513668  0.08296573  1.06916146
#> [37]  0.48202950  1.36443493  0.24621084  0.67726849
```

A histogram of all 10,000 realizations of $y_{1,1}$ may be obtained from

```
# output omitted
hist(approx_bold_Y_draw[1, 1, ])
```

The matrices $\mathbf{R}_1$, $\mathbf{R}_2$, $\mathbf{R}$, $\mathbf{r}_1$, $\mathbf{r}_2$, and $\mathbf{r}$ are similarly structured except that they represent i.i.d. Bernoulli random variables with outcomes of one (observed) or zero (missing). Let $\mathbf{R}_1$ be an i.i.d. Bernoulli random variable with probability of observance 0.9 and $\mathbf{R}_2$ be an i.i.d. Bernoulli random variable with probability of observance 0.7. We first create a function called `bold_r` that simulates a single realization of $\mathbf{r}_1$ and $\mathbf{r}_2$ and places them into the $\mathbf{r}$ matrix.

```
bold_r <- function() {
    r_1 <- rbinom(n = 6, size = 1, prob = 0.9)
```

```r
    r_2 <- rbinom(n = 6, size = 1, prob = 0.7)
    r <- matrix(c(r_1, r_2), nrow = 6, ncol = 2)
    return(r)
}
```

A single realization of **r** looks like:

```r
set.seed(1)
r_draw_one <- bold_r()
print(r_draw_one)
#>      [,1] [,2]
#> [1,]    1    0
#> [2,]    1    1
#> [3,]    1    1
#> [4,]    0    1
#> [5,]    1    1
#> [6,]    1    1
```

Another realization of **r** looks like:

```r
r_draw_two <- bold_r()
print(r_draw_two)
#>      [,1] [,2]
#> [1,]    1    1
#> [2,]    1    0
#> [3,]    1    0
#> [4,]    1    1
#> [5,]    1    1
#> [6,]    0    1
```

We can approximate the distribution of $\mathbf{R}$ using the `approx_bold_R` function, which simulates `s` different $\mathbf{r}$'s

```
approx_bold_R <- function(s) {
    R <- replicate(s, bold_r())
    return(R)
}
```

The larger the `s`, the closer the approximation to $\mathbf{R}$.

The following code generates 10,000 realizations of $\mathbf{r}$ and places them into an an array. The dimensions of the array are $6 \times 2 \times 10,000$:

```
set.seed(1)
approx_bold_R_draw <- approx_bold_R(s = 10000)
```

The first realization (6×2 array element) of $\mathbf{r}$ matches `r_draw_one`:

```
print(approx_bold_R_draw[, , 1])
#>      [,1] [,2]
#> [1,]    1    0
#> [2,]    1    1
#> [3,]    1    1
#> [4,]    0    1
#> [5,]    1    1
#> [6,]    1    1
```

The 10,000th realization of of $\mathbf{r}$ is

```
print(approx_bold_R_draw[, , 10000])
#>      [,1] [,2]
#> [1,]    1    1
#> [2,]    1    0
```

```
#> [3,]    1    1
#> [4,]    1    1
#> [5,]    1    1
#> [6,]    1    0
```

The first 40 realizations of $r_{1,1}$ are

```
head(approx_bold_R_draw[1, 1, ], n = 40)
#>  [1] 1 1 1 1 1 0 1 1 1 0 0 1 1 1 1 1 1 1 1 1 1 1 1 1 1 1 1 1 1 1 1 0 1 1 1 1 1 1
#> [39] 1 1
```

A histogram of all 10,000 realizations of $r_{1,1}$ may be obtained from

```
# output omitted
hist(approx_bold_R_draw[1, 1, ])
```

Subsetting each $\mathbf{y}$ by its corresponding $\mathbf{r}$ yields $\mathbf{y}_{obs}$ (for $\mathbf{r}$ elements that equal one) and $\mathbf{y}_{mis}$ (for $\mathbf{r}$ elements that equal zero).

Appendix S2

Missing data in ecology: Syntheses, clarifications, and considerations

Michael Dumelle, Rob Trangucci, Amanda M. Nahlik, Anthony R. Olsen, Kathryn M. Irvine, Karen Blocksom, Jay M. Ver Hoef, Claudio Fuentes

Ecological Monographs

Section S1 A Comparison of Various Missing Data Approaches for MAR Missing Data

In the main text, we compared 13 missing data approaches for MAR missing data. Here we compare 19, adding both the single and multiple imputation versions of Bayesian Regression Imputation (Rubin, 2004; Box and Tiao, 2011), Lasso Imputation (Deng et al., 2016; Zhao and Long, 2016), and Random Forest Imputation (Shah et al., 2014). Table S1 provides details for all 19 approaches.

Inferential Performance

Table S2 provides inferential performance for all 19 approaches.

Prediction Performance

Table S3 provides prediction performance for all 19 approaches.

Type-0 and Type-1 Predictive Mean Matching

Predictive mean matching chooses a donor for imputation by comparing the closeness between the fitted value of an item for each unit in the complete data and the fitted value for an item requiring imputation. The fitted value for the ith item from the complete data is $\hat{y}_i$ and the fitted value for the item from the new unit is $\hat{y}_{imp}$.

Approach	Abbreviation	Imputations	Parameters
Complete Case Analysis	CCA	-	-
Mean Imputation	Mean	Deterministic	Deterministic
Regression Imputation	Reg	Deterministic	Deterministic
Nearest Neighbor Imputation	NN	Deterministic	Deterministic
Stochastic Regression Imputation (Single)	StReg-S	Stochastic	Deterministic
Predictive Mean Matching Type-0 Imputation (Single)	PMMT0-S	Stochastic	Deterministic
Bootstrap Regression Imputation (Single)	Boot-S	Stochastic	Stochastic
Predictive Mean Matching Type-1 Imputation (Single)	PMMT1-S	Stochastic	Stochastic
Bayesian Regression Imputation (Single)	BReg-S	Stochastic	Stochastic
Lasso Regression Imputation (Single)	LReg-S	Stochastic	Stochastic
Stochastic Forest Imputation (Single)	RF-S	Stochastic	Stochastic
Stochastic Regression Imputation (Multiple)	StReg-M	Stochastic	Deterministic
Predictive Mean Matching Type-0 Imputation (Multiple)	PMMT0-M	Stochastic	Deterministic
Bootstrap Regression Imputation (Multiple)	Boot-M	Stochastic	Stochastic
Predictive Mean Matching Type-1 Imputation (Multiple)	PMMT1-M	Stochastic	Stochastic
Bayesian Regression Imputation (Multiple)	BReg-M	Stochastic	Stochastic
Lasso Regression Imputation (Multiple)	LReg-M	Stochastic	Stochastic
Stochastic Forest Imputation (Multiple)	RF-M	Stochastic	Stochastic
Fully Bayesian Data Augmentation	FBDA	Stochastic	Stochastic

Table S1: Missing data approaches and abbreviations used for the comparison of various missing data approaches for MAR missing data in the main text. The "Imputations" column clarifies whether the imputation process is deterministic (i.e., nonrandom) or stochastic (i.e., random). The "Parameters" column clarifies whether the parameters used to fill in missing data items are deterministic or stochastic.

Type-0 and Type-1 predictive mean matching use a linear regression formulation to determine $\hat{\mathrm{y}}_i$ and $\hat{\mathrm{y}}_{imp}$. In Type-0 matching,

$$\hat{\mathrm{y}}_i = \mathbf{x}_i \hat{\boldsymbol{\beta}} \text{ and } \hat{\mathrm{y}}_{imp} = \mathbf{x}_{imp} \hat{\boldsymbol{\beta}},$$

where $\hat{\boldsymbol{\beta}}$ is an estimate of $\boldsymbol{\beta}$ (e.g., from ordinary least squares, posterior median, etc.). In Type-1 matching,

$$\hat{\mathrm{y}}_i = \mathbf{x}_i \hat{\boldsymbol{\beta}} \text{ and } \hat{\mathrm{y}}_{imp} = \mathbf{x}_{imp} \tilde{\boldsymbol{\beta}}$$

where $\hat{\boldsymbol{\beta}}$ is an estimate of $\boldsymbol{\beta}$ and $\tilde{\boldsymbol{\beta}}$ is a draw from the posterior distribution of $\boldsymbol{\beta}$ based on a Bayesian linear regression model (White et al., 2011; Van Buuren, 2018). Type-0 matching is

	β_1				β_2			
Approach	MBias	RMSE	S.E.	Cover95	MBias	RMSE	S.E.	Cover95
CCA	0.00	0.49	0.50	0.95	-0.00	0.26	0.27	0.95
Mean	0.59	0.62	0.19	0.16	0.59	0.61	0.15	0.06
Reg	0.00	0.47	0.09	0.31	-0.31	0.40	0.06	0.12
NN	0.04	0.51	0.24	0.65	0.32	0.45	0.13	0.38
StReg-S	0.00	0.46	0.21	0.60	0.02	0.26	0.11	0.59
PMM-3D-S-T0	0.08	0.46	0.21	0.62	0.10	0.31	0.11	0.55
Boot-S	-0.00	0.54	0.20	0.52	0.00	0.28	0.10	0.55
PMM-3D-S-T1	0.07	0.52	0.21	0.56	0.12	0.33	0.11	0.53
BReg-S	-0.01	0.53	0.21	0.57	0.05	0.31	0.11	0.53
LReg-S	0.04	0.52	0.20	0.52	0.00	0.31	0.10	0.52
RF-S	0.04	0.45	0.27	0.76	0.62	0.65	0.14	0.07
StReg-M	-0.00	0.40	0.31	0.87	0.02	0.22	0.19	0.91
PMM-3D-M-T0	0.08	0.40	0.31	0.88	0.11	0.25	0.21	0.90
Boot-M	0.00	0.40	0.41	0.95	0.00	0.22	0.23	0.95
PMM-3D-M-T1	0.07	0.39	0.41	0.96	0.12	0.26	0.24	0.94
BReg-M	-0.00	0.40	0.42	0.96	0.06	0.22	0.25	0.97
LReg-M	0.03	0.40	0.39	0.94	-0.01	0.23	0.24	0.95
RF-M	0.05	0.41	0.35	0.90	0.63	0.64	0.22	0.11
FBDA	0.00	0.38	0.39	0.95	0.04	0.22	0.24	0.95

Table S2: For the comparison of various missing data approaches for MAR missing data in the main text, missing data approach inferential performance across 2,000 independent simulation trials. Mean bias (MBias), root-mean-squared error (RMSE), mean standard error (S.E.), and 95% confidence interval coverage (Cover95) are reported separately for the β_1 and β_2 slope parameters. The Approach and Cover95 columns are bolded when the approach has proper Cover95 (i.e., between 0.94 and 0.96).

deterministic (i.e., nonrandom), while Type-1 matching is stochastic (i.e., random). The stochastic component of Type-1 matching is the random draw from the posterior distribution of β.

Fully Bayesian Data Augmentation Model Specification

Let $\mathrm{r}_i^{\mathrm{y}}$ be the observation indicator for y_i and let $\mathrm{r}_i^{\mathrm{x}_2}$ be the observation indicator for $\mathrm{x}_{2,i}$. Let $(1, \mathrm{x}_{1,i}, \mathrm{x}_{2,i})$ be the row-vector of predictors for unit i. Let $[\mathrm{y}_i \mid \mu_i, \sigma^2]$ be the (univariate) Gaussian density function corresponding with mean μ_i and variance σ^2 evaluated at y. The fully Bayesian data augmentation (FBDA) model specification used in the simulation study treats missing y_i and $\mathrm{x}_{2,i}$ as unknown parameters; thus we denote these parameters as $\dot{\mathrm{y}}_i$ and $\dot{\mathrm{x}}_{2,i}$. We assume the

	Predictions			
Approach	MPBias	RMSPE	S.E.	PCover95
CCA	-0.01	1.08	1.18	0.96
Mean	-0.21	1.24	0.99	0.88
Reg	0.00	1.12	0.46	0.57
NN	-0.02	1.17	1.20	0.96
StReg-S	-0.00	1.09	1.03	0.92
PMM-3D-S-T0	-0.02	1.10	1.05	0.93
Boot-S	-0.02	1.11	1.00	0.90
PMM-3D-S-T1	-0.02	1.12	1.06	0.92
BReg-S	0.01	1.12	1.06	0.92
LReg-S	-0.00	1.12	0.97	0.89
RF-S	-0.03	1.22	1.35	0.96
StReg-M	-0.01	1.06	1.05	0.94
PMM-3D-M-T0	-0.02	1.07	1.08	0.94
Boot-M	-0.01	1.06	1.05	0.94
PMM-3D-M-T1	-0.02	1.07	1.11	0.95
BReg-M	-0.01	1.06	1.12	0.95
LReg-M	-0.01	1.07	1.02	0.93
RF-M	-0.04	1.22	1.37	0.97
FBDA	-0.01	1.06	1.13	0.95

Table S3: For the comparison of various missing data approaches for MAR missing data in the main text, missing data approach prediction performance across 2,000 independent simulation trials. Mean prediction bias (MPBias), root-mean-squared-prediction error (RMPSE), mean prediction standard error (S.E.), and 95% prediction interval coverage (PCover95) are reported for predictions. The Approach and PCover95 columns are bolded when the approach has proper PCover95 (i.e., between 0.94 and 0.96).

following generative model for our data:

$$\begin{aligned} [\mathrm{x}_{2,i} \mid \mu_x, \sigma_x^2] &\sim \text{Gaussian}(\mu_x, \sigma_x^2) \\ [\mathrm{y}_i \mid \mathrm{x}_{1,i}, \mathrm{x}_{2,i}, \boldsymbol{\beta}, \sigma_y^2] &\sim \text{Gaussian}((1, \mathrm{x}_{1,i}, \mathrm{x}_{2,i})\boldsymbol{\beta}, \sigma_y^2) \end{aligned} \tag{S1}$$

The full set of parameters is as follows:

$$\boldsymbol{\beta}, \sigma_y^2, \sigma_x^2, \mu_x, \{\dot{\mathrm{y}}_i \mid \mathrm{r}_i^{\mathrm{y}} = 0, i = 1, \ldots, n\}, \{\dot{\mathrm{x}}_{2,i} \mid \mathrm{r}_i^{\mathrm{x}} = 0, i = 1, \ldots, n\}.$$

We subsequently use the abbreviations $\{\dot{\mathrm{y}}_i\} \equiv \{\dot{\mathrm{y}}_i \mid \mathrm{r}_i^{\mathrm{y}} = 0, i = 1, \ldots, n\}$ and $\{\dot{\mathrm{x}}_{2,i}\} \equiv \{\dot{\mathrm{x}}_{2,i} \mid \mathrm{r}_i^{\mathrm{x}} = 0, i = 1, \ldots, n\}$. Under the assumed model (1), the likelihood for the

unknown parameters $\boldsymbol{\beta}, \sigma_y^2, \sigma_x^2, \mu_x, \{\dot{\text{y}}_i\}, \{\dot{\text{x}}_{2,i}\}$ is as follows:

$$
\begin{aligned}
&\prod_{i|\text{r}_i^{\text{y}}=1,\text{r}_i^{X}=1} [\text{y}_i \mid (1, \text{x}_{1,i}, \text{x}_{2,i})\boldsymbol{\beta}, \sigma_y^2][\text{x}_{2,i} \mid \mu_x, \sigma_x^2] \\
\times &\prod_{i|\text{r}_i^{\text{y}}=1,\text{r}_i^{\text{x}}=0} [\text{y}_i \mid (1, \text{x}_{1,i}, \dot{\text{x}}_{2,i})\boldsymbol{\beta}, \sigma_y^2] \\
\times &\prod_{i|\text{r}_i^{\text{y}}=0,\text{r}_i^{\text{x}}=1} [\text{x}_{2,i} \mid \mu_x, \sigma_x^2]
\end{aligned}
$$

This likelihood coupled with the priors implied for unknown parameters $\{\dot{\text{y}}_i\}, \{\dot{\text{x}}_{2,i}\}$ and the unknown parameters $\boldsymbol{\beta}, \sigma_y^2, \mu_x, \sigma_x^2$ yields the joint posterior over the unknown parameters:

$$
\begin{aligned}
p[\boldsymbol{\beta}, \sigma_y^2, \sigma_x^2, \mu_x, \{\dot{\text{y}}_i\}, \{\dot{x}_{2,i}\} \mid \text{y}_{(1)}, \text{x}_1, \text{x}_{2(1)}] \propto &\prod_{i|\text{r}_i^{\text{y}}=1,\text{r}_i^{\text{x}}=1} [\text{y}_i \mid (1, \text{x}_{1,i}, \text{x}_{2,i})\boldsymbol{\beta}, \sigma_y^2][\text{x}_{2,i} \mid \mu_x, \sigma_x^2] \\
\times &\prod_{i|\text{r}_i^{\text{y}}=1,\text{r}_i^{\text{x}}=0} [\text{y}_i \mid (1, \text{x}_{1,i}, \dot{\text{x}}_{2,i})\boldsymbol{\beta}, \sigma_y^2][\dot{\text{x}}_{2,i} \mid \mu_x, \sigma_x^2] \\
\times &\prod_{i|\text{r}_i^{\text{y}}=0,\text{r}_i^{\text{x}}=1} [\dot{\text{y}}_i \mid (1, \text{x}_{1,i}, \text{x}_{2,i})\boldsymbol{\beta}, \sigma_y^2][\text{x}_{2,i} \mid \mu_x, \sigma_x^2] \\
\times &\prod_{i|\text{r}_i^{\text{y}}=0,\text{r}_i^{\text{x}}=0} [\dot{\text{y}}_i \mid (1, \text{x}_{1,i}, \dot{\text{x}}_{2,i})\boldsymbol{\beta}, \sigma_y^2][\dot{\text{x}}_{2,i} \mid \mu_x, \sigma_x^2] \\
\times &\, \pi[\boldsymbol{\beta}]\pi[\sigma_y][\sigma_x][\mu_x].
\end{aligned}
$$

The priors $\pi[\boldsymbol{\beta}]$, $\pi[\mu_x]$, $\pi[\sigma_y]$, and $\pi[\sigma_x]$ are `brms`'s default priors for the slope parmaeters parameters, means, and standard deviations, respectively (Bürkner, 2017).

Recovering Missing Items

In the main text, we argued the primary goal of a statistical analysis is to appropriately estimate population quantities (e.g., population mean, regression coefficient) and characterize uncertainty in those estimates. Hence, imputation methods should be evaluated on their capacity to achieve these goals by yielding estimates that are unbiased and efficient with correct confidence interval coverage. It may seem natural and intuitive to instead to evaluate imputation methods by

comparing imputed items to their true values, but recovering missing items is typically not the primary analysis goal. Schafer and Graham (2002) state that "With or without missing data, the goal of a statistical procedure should be to make valid and efficient inferences about a population of interest – not to recover missing observations ... A missing value treatment cannot be separated from the modeling, estimation, or testing procedure from which it is embedded," while Van Buuren (2018) state that "measures based on similarity between the true and imputed values do not separate valid from invalid imputation methods." Murray (2018), Yuan (2010), and Little and Rubin (2019), among others, make similar clarifications. Here, we provide a numerical example that shows how evaluating imputation methods by their ability to recover true missing items can be misleading when the statistical analysis goals are parameter estimation, prediction, and uncertainty quantification. We focus on two deterministic imputation methods, mean imputation (Mean) and and regression imputation (Reg) and two stochastic imputation methods, single bootstrap regression imputation (Boot-S) and multiple bootstrap regression imputation (Boot-M).

In each simulation trial, we computed mean bias (MBias) and root-mean-squared error of the of estimates relative to the slope coefficients, β_1 and β_2. We can also evaluate the capacity for each imputation method to recover missing $\mathbf{x}_2$ items by evaluating imputation MBias and imputation RMSE. More formally, imputation Mbias for $\mathbf{x}_2$ is

$$\text{MBias}(\text{Imp}(\mathbf{x}_2)) = \frac{1}{2000 \times J} \sum_{i=1}^{2{,}000} \sum_{j} (\tilde{\mathrm{x}}_{2,j} - \mathrm{x}_{2,j}),$$

where i indexes the simulation trials, j indexes the J missing items for $\mathbf{x}_2$ within a simulation trial (which vary from trial to trial), $\tilde{\mathrm{x}}_{2,j}$ is the imputed value and $\mathrm{x}_{2,j}$ is the true value. Similarly, imputation RMSPE for $\mathbf{x}_2$ is

$$\text{RMSE}(\text{Imp}(\mathbf{x}_2)) = \sqrt{\frac{1}{2000 \times J} \sum_{i=1}^{2{,}000} \sum_{j} (\tilde{\mathrm{x}}_{2,j} - \mathrm{x}_{2,j})^2}.$$

Table S4 shows the performance for inference regarding β_2 alongside the recovery of the missing $\mathbf{x}_2$ items used. Recall that Mean and Reg were biased and had high (poor) RMSE and low coverage for β_2. Boot-S and Boot-M were unbiased for β_2, but Boot-M was more efficient (lower RMSE) than Boot-S and Boot-M had proper coverage for β_2. For Imp($\mathbf{x}_2$), all approaches were unbiased, while Mean and Reg had lower RMSE than Boot-S and Boot-M.

	β_2			Imp($\mathbf{x}_2$)	
Approach	MBias	RMSE	Cover95	MBias	RMSE
Mean	0.59	0.61	0.06	-0.00	1.01
Reg	-0.31	0.40	0.12	-0.00	0.92
Boot-S	0.00	0.28	0.55	-0.00	1.25
Boot-M	0.00	0.22	0.95	0.00	1.25

Table S4: For the comparison of various missing data approaches for MAR missing data in the main text, missing data approach performance across 2,000 independent simulation trials for recovering missing items (via imputation) of $\mathbf{x}_2$ (Imp($\mathbf{x}_2$)). Mean bias (MBias) and root-mean-squared error (RMSE) are reported.

In each simulation trial, we computed mean bias (MBias) and root-mean-squared error of the prediction relative to y_{new}, the value of the response variable in the test data. We can also evaluate the capacity for each imputation method to recover missing $\mathbf{y}$ items (in the training data) by evaluating imputation MBias and imputation RMSE. More formally, imputation Mbias for $\mathbf{y}$ is

$$\text{MBias}(\text{Imp}(\mathbf{y})) = \frac{1}{2000 \times J} \sum_{i=1}^{2{,}000} \sum_{j} (\tilde{\mathrm{y}}_j - \mathrm{y}_j),$$

where i indexes the simulation trials, j indexes the J missing items for $\mathbf{y}$ within a simulation trial (which vary from trial to trial), $\tilde{\mathrm{y}}_j$ is the imputed value and y_j is the true value. Similarly, imputation RMSPE for $\mathbf{y}$ is

$$\text{RMSE}(\text{Imp}(\mathbf{x}_2)) = \sqrt{\frac{1}{2000 \times J} \sum_{i=1}^{2{,}000} \sum_{j} (\tilde{\mathrm{x}}_{2,j} - \mathrm{x}_{2,j})^2}.$$

Table S5 shows the performance for prediction of y_{new} (the value of the response variable in the test data) alongside the recovery of the missing $\mathbf{y}$ items used in the training data. Recall that mean imputation was biased and had high (poor) RMSE and low coverage for y_{new}. Regression

imputation was unbiased but had low coverage for y_{new}. Boot-S and Boot-M were unbiased for y_{new}, but Boot-M was more efficient (lower RMSE) than Boot-S and Boot-M had proper coverage for y_{new}. For Imp($\mathbf{y}$), all approaches were unbiased except Mean, while Mean and Reg had lower RMSE than Boot-S and Boot-M.

	Predictions			Imp($\mathbf{y}$)	
Approach	MPBias	RMSPE	PCover95	MBias	RMSE
Mean	-0.21	1.24	0.88	-0.35	1.54
Reg	0.00	1.12	0.57	0.00	1.30
Boot-S	-0.02	1.11	0.90	0.00	1.77
Boot-M	-0.01	1.06	0.94	0.01	1.77

Table S5: For the comparison of various missing data approaches for MAR missing data in the main text, missing data approach performance across 2,000 independent simulation trials for recovering missing items (via imputation) of $\mathbf{y}$ (Imp($\mathbf{y}$)). Mean bias (MBias) and root-mean-squared error (RMSE) are reported.

Notice that in Table S4 and Table S5, Mean and Reg have lower (better) RMSE for Imp($\mathbf{x}_2$) and Imp($\mathbf{y}$) compared to Boot-S and Boot-M. However, Mean and Reg have have higher (worse) RMSE for β_2 and y_{new} compared to Boot-S and Boot-M, are sometimes biased, and never have proper coverage. Together, these results showcase that evaluating imputation methods by their ability to recover true missing items can be misleading when the statistical analysis goals are parameter estimation, prediction, and uncertainty quantification.

References

Box, G.E., Tiao, G.C., 2011. Bayesian inference in statistical analysis. John Wiley & Sons.

Bürkner, P.C., 2017. brms: An R package for Bayesian multilevel models using Stan. Journal of Statistical Software 80, 1–28.

Deng, Y., Chang, C., Ido, M.S., Long, Q., 2016. Multiple imputation for general missing data patterns in the presence of high-dimensional data. Scientific Reports 6, 21689.

Little, R.J., Rubin, D.B., 2019. Statistical analysis with missing data. 3rd ed., John Wiley & Sons.

Murray, J.S., 2018. Multiple imputation: A review of practical and theoretical findings. Statistical Science 33, 142–159.

Rubin, D.B., 2004. Multiple imputation for nonresponse in surveys. John Wiley & Sons.

Schafer, J.L., Graham, J.W., 2002. Missing data: Our view of the state of the art. Psychological Methods 7, 147.

Shah, A.D., Bartlett, J.W., Carpenter, J., Nicholas, O., Hemingway, H., 2014. Comparison of random forest and parametric imputation models for imputing missing data using MICE: A CALIBER study. American journal of epidemiology 179, 764–774.

Van Buuren, S., 2018. Flexible imputation of missing data. CRC press.

White, I.R., Royston, P., Wood, A.M., 2011. Multiple imputation using chained equations: Issues and guidance for practice. Statistics in Medicine 30, 377–399.

Yuan, Y.C., 2010. Multiple imputation for missing data: Concepts and new development (version 9.0). SAS Institute Inc, Rockville, MD 49, 12.

Zhao, Y., Long, Q., 2016. Multiple imputation in the presence of high-dimensional data. Statistical Methods in Medical Research 25, 2021–2035.

Appendix S3

Missing data in ecology: Syntheses, clarifications, and considerations

Michael Dumelle, Rob Trangucci, Amanda M. Nahlik, Anthony R. Olsen, Kathryn M. Irvine, Karen Blocksom, Jay M. Ver Hoef, Claudio Fuentes

Ecological Monographs

Section S1 A Comparison of Various Missing Data Approaches for MNAR Missing Data

Inferential Performance

Table S1 provides inferential performance for all four approaches applied to the three scenarios. All approaches have some (absolute) MBias, but this should decrease as the sample size increases. MBias tends to decrease as the MNAR amount also decreases. The Heckman model always has the lowest RMSE for all MNAR amounts, while the other three approaches tend to have similar RMSE. When the MNAR amount of large, the Heckman and PM-Large have nearly proper coverage. When the MNAR amount is moderate, the Heckman, PM-Large, and PM-Medium approaches have proper or nearly proper coverage. When the MNAR amount is small, the MAR approach has proper coverage while the MNAR approaches (Heckman, PM-Large, PM-Moderate) slightly overcover.

Prediction Performance

Table S2 provides prediction performance for all four approaches applied to the three scenarios. All approaches (except Heckman with small MNAR amount) have some (absolute) MPBias, but this should decrease as the sample size increases. MPBias tends to decrease as the MNAR amount also decreases. The Heckman model always has the lowest RMPSE for all MNAR amounts, while the other three approaches tend to have similar RMPSE. When the MNAR amount

		β_1				β_2			
Approach	MNAR Amount	MBias	RMSE	S.E.	Cover95	MBias	RMSE	S.E.	Cover95
MAR	Large	-0.38	0.44	0.24	0.64	-0.38	0.41	0.14	0.24
Heckman	Large	-0.19	0.31	0.28	0.91	-0.16	0.24	0.21	0.91
PM-Large	Large	-0.38	0.44	0.42	0.92	-0.38	0.41	0.35	0.92
PM-Moderate	Large	-0.38	0.44	0.28	0.76	-0.38	0.41	0.19	0.55
MAR	Moderate	-0.22	0.34	0.28	0.89	-0.22	0.27	0.16	0.74
Heckman	Moderate	-0.14	0.29	0.32	0.96	-0.11	0.21	0.24	0.97
PM-Large	Moderate	-0.22	0.34	0.50	0.98	-0.22	0.27	0.43	0.99
PM-Moderate	Moderate	-0.22	0.34	0.32	0.93	-0.22	0.27	0.23	0.91
MAR	Small	-0.08	0.29	0.29	0.96	-0.06	0.17	0.17	0.94
Heckman	Small	-0.06	0.27	0.33	0.98	-0.02	0.17	0.25	0.99
PM-Large	Small	-0.08	0.29	0.56	1.00	-0.06	0.17	0.51	1.00
PM-Moderate	Small	-0.08	0.29	0.35	0.98	-0.06	0.17	0.26	0.99

Table S1: For the comparison of various missing data approaches for MNAR missing data in the main text, missing data approach inferential performance across 2,000 independent simulation trials. Mean bias (MBias), root-mean-squared error (RMSE) and 95% confidence interval coverage (Cover95) are reported separately for the β_1 and β_2 slope parameters.

of large, the Heckman approach has nearly proper coverage. When the MNAR amount is moderate, the Heckman and PM-Medium approaches have proper coverage. When the MNAR amount is small, the MAR approach has proper coverage while the MNAR approaches (Heckman, PM-Large, PM-Moderate) slightly overcover.

		Predictions			
Approach	MNAR Amount	MPBias	RMPSE	S.E.	PCover95
MAR	Large	0.69	1.30	0.85	0.80
Heckman	Large	0.23	1.08	1.02	0.93
PM-Large	Large	0.69	1.29	1.73	0.98
PM-Moderate	Large	0.69	1.29	0.98	0.87
MAR	Moderate	0.36	1.11	1.00	0.92
Heckman	Moderate	0.15	1.07	1.14	0.96
PM-Large	Moderate	0.36	1.11	1.68	0.99
PM-Moderate	Moderate	0.36	1.11	1.12	0.95
MAR	Small	0.05	1.03	1.05	0.96
Heckman	Small	-0.03	1.05	1.18	0.97
PM-Large	Small	0.05	1.03	1.62	0.99
PM-Moderate	Small	0.05	1.03	1.18	0.97

Table S2: For the comparison of various missing data approaches for MNAR missing data in the main text, missing data approach prediction performance across 2,000 independent simulation trials. Mean prediction bias (MPBias), root-mean-squared-prediction error (RMPSE), mean prediction standard errors (S.E.), and 95% prediction interval coverage (PCover95) are reported for predictions.

Appendix S4

Missing data in ecology: Syntheses, clarifications, and considerations

Michael Dumelle, Rob Trangucci, Amanda M. Nahlik, Anthony R. Olsen, Kathryn M. Irvine, Karen Blocksom, Jay M. Ver Hoef, Claudio Fuentes

Ecological Monographs

Section S1 Two Applications of Contingency Variables

Here we explore two of several ways contingency variables can be analyzed. First, we analyze the contingency variable of surface water presence in wetlands to estimate the proportion of wetlands in the conterminous United States (CONUS) having surface water. Second, we use the contingency variable surface water presence in the explanatory variable structure for a spatial regression model studying VMMI.

Estimating the Proportion of CONUS Wetlands with Surface Water and an Extension

The 2016 NWCA recorded whether each wetland (i.e., unit, site) had surface water present. We can leverage the 2016 NWCA survey design combined with design-based inference to estimate the relative proportion of each level of the contingency variable, surface water presence. That is, we can estimate the proportion of wetlands with and without surface water in the broader CONUS population. Using the IPW weights from the 2016 NWCA survey design, the Horvitz-Thompson estimator (Horvitz and Thompson, 1952; Cordy, 1993), the local neighborhood variance estimator (Stevens Jr and Olsen, 2003), and the `spsurvey` **R** package (Dumelle et al., 2023b), we estimated that approximately 60% of CONUS wetlands have surface water and 40% do not. Figure S1B displays these estimates alongside corresponding uncertainties.

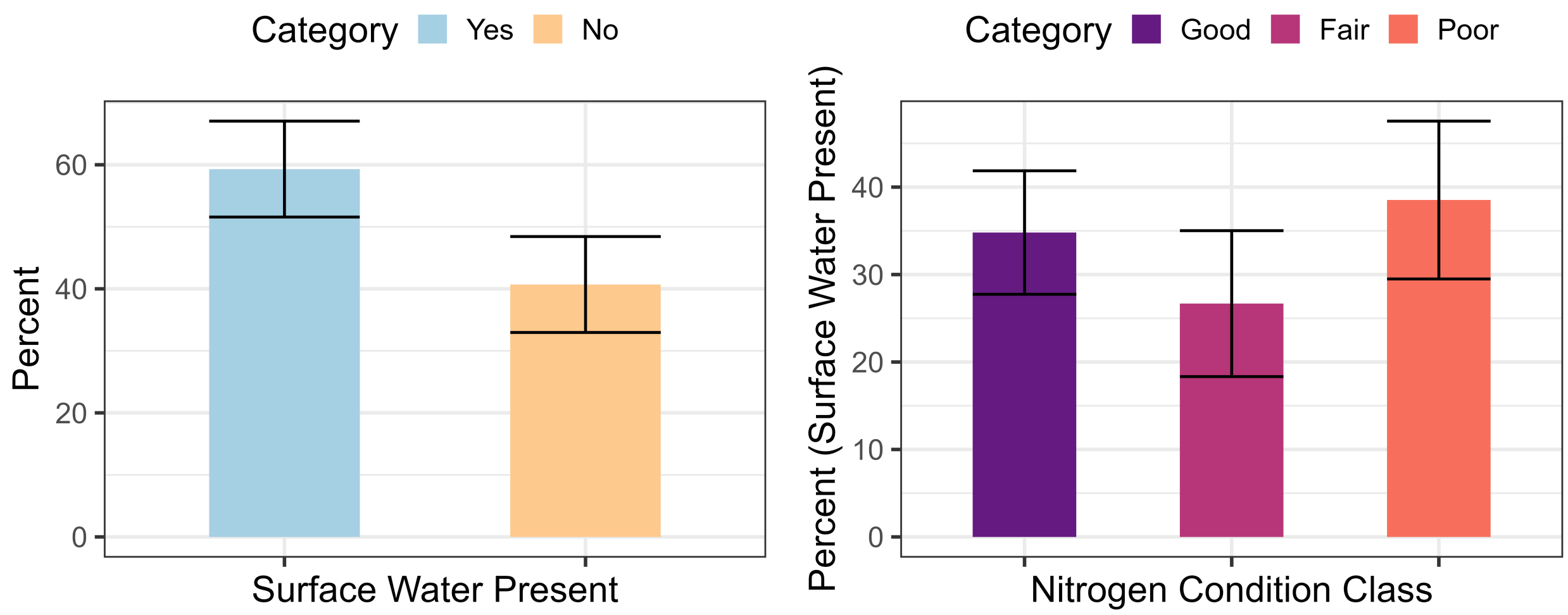

(A) (B)

Figure S1: In (A), proportion of CONUS wetlands with surface water. In (B), the proportion of CONUS wetlands with surface water that are in Good, Fair, or Poor TN condition. 95% confidence intervals for the estimates in (A) and (B) are represented by black bars.

For wetlands with surface water, the 2016 NWCA also assessed the total nitrogen (TN) condition as being Good, Fair, or Poor. TN in surface water is only measurable at wetlands with surface water, and the condition category is based on measured TN compared to a reference (i.e., undisturbed) benchmark (Stoddard et al., 2006; USEPA, 2023) at the wetland (Figure S2). Using a similar approach as with estimating surface water presence, we estimated that among CONUS wetlands with surface water, approximately 34% of wetlands are in Good condition for TN, 26% of wetlands are in Fair condition for TN, 40% of wetlands are in Poor condition for TN (Figure S1B).

In the first example (Figure S1A), we estimated the relative proportion of each level of the contingency variable in the CONUS wetland population. In the second example (Figure S1B), we estimated the relative proportion of each condition category (Good, Fair, Poor) for wetlands conditioned on one level of the contingency variable (here, that surface water was present).

A Spatial Linear Regression Model for VMMI using Surface Water Presence

Contingency variables may also be used in the explanatory variable structure of a statistical model. Recall in the main text, we used a spatial linear regression model to study the impact of

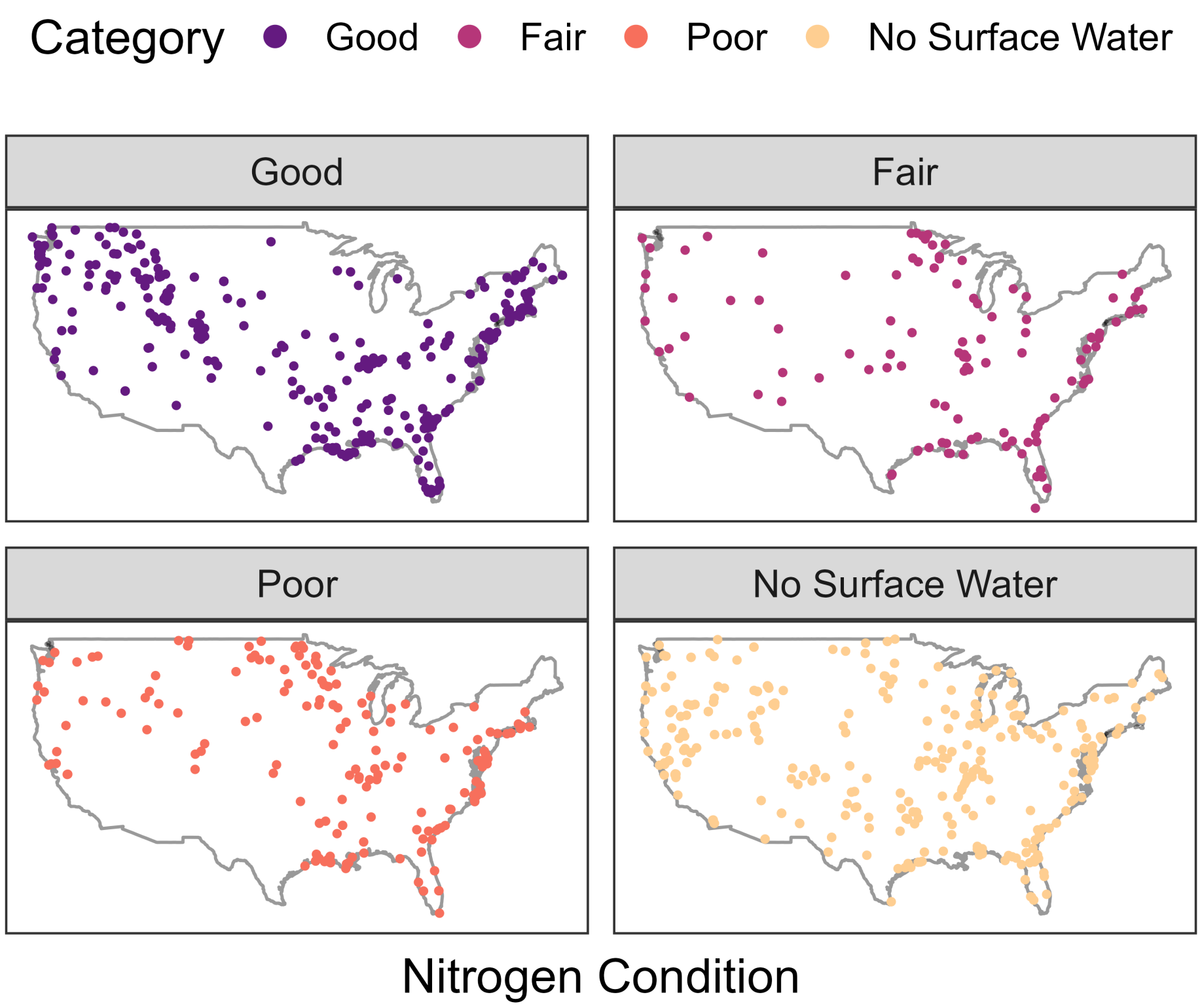

Figure S2: 2016 NWCA surface water presence and total nitrogen (TN) condition categories. Wetlands with surface water presence are grouped into Good, Fair, or Poor condition for TN.

soil hardening (SH) and wetland type (WT) on a vegetation multi-metric index (VMMI). Suppose that we want to supplement this model to account for surface water presence and total nitrogen at the wetland (when surface water is present); the model structure may look like:

$$\text{VMMI}_i = \beta_0 + \beta_1 \mathcal{I}(\text{VT}_i) + \beta_2 \text{SH}_i + \beta_3 \mathcal{I}(\text{SWP}_i) + \beta_4 \mathcal{I}(\text{SWP}_i) \log(\text{TN}_i) + \tau_i + \epsilon_i,$$

where

$$\mathcal{I}(\text{SWP}_i) = \begin{cases} 1 & \text{if surface water is present} \\ 0 & \text{if surface water is not present,} \end{cases}$$

and where

$$\mathcal{I}(\text{SWP}_i) \log(\text{TN}_i) = \begin{cases} \log(\text{TN}_i) & \text{if surface water is present} \\ 0 & \text{if surface water is not present.} \end{cases}$$

The slope coefficient β_3 captures the average effect of surface water presence on VMMI, and the slope coefficient β_4 captures the average effect of the natural logarithm of total nitrogen (a common transformation fo chemical concentrations) on VMMI at wetlands with surface water. The interaction between $\mathcal{I}(\text{SWP}_i)$ and $\log(\text{TN}_i)$ enables the model to incorporate the effect of log(TN) while still accounting for other variables like VT, SH, and SWP observable at all wetlands. This is preferred to subsetting the data to include only wetlands with surface water, because subsetting the data omits valuable information elucidating the impact of VT, SH, and SWP on VMMI.

After fitting the model in Equation 1, we found (Table S1) that 1) there is strong evidence that soil hardening is negatively associated with average VMMI (p-value $\approx$ 0.001), 2) there is strong evidence that woody wetlands are associated with a lower average VMMI than herbaceous wetlands (p-value $<$ 0.001), 3) there is moderate evidence that wetlands with surface water are

associated with a higher average VMMI than wetlands without surface water (p-value ≈ 0.04), and 4) there is little evidence that, for wetlands with surface water, total nitrogen is associated with average VMMI (p-value > 0.1). We also found that the spatial linear regression model was a much better fit to the data than its nonspatial counterpart (spatial AIC = 8237, nonspatial AIC = 8532; spatial BIC = 8252, nonspatial BIC = 8537; spatial leave-one-out RMSPE = 16.7, nonspatial leave-one-out RMSPE = 20.5). The spatial model was fit using the `spmodel` **R** package (Dumelle et al., 2023a).

Parameter	Estimate	SE	p-value
β_0	63.64	10.08	< 0.001
β_1	-0.09	0.03	0.001
β_2	-10.67	1.25	< 0.001
β_3	2.57	1.25	0.039
β_4	-0.54	0.72	0.452

Table S1: Parameter estimates, standard errors, and p-values for the spatial linear regression model.

References

Cordy, C.B., 1993. An extension of the Horvitz-Thompson theorem to point sampling from a continuous universe. Statistics & Probability Letters 18, 353–362.

Dumelle, M., Higham, M., Ver Hoef, J.M., 2023a. spmodel: Spatial statistical modeling and prediction in R. PLOS ONE 18, e0282524.

Dumelle, M., Kincaid, T., Olsen, A.R., Weber, M., 2023b. spsurvey: Spatial sampling design and analysis in R. Journal of Statistical Software 105, 1–29.

Horvitz, D.G., Thompson, D.J., 1952. A generalization of sampling without replacement from a finite universe. Journal of the American Statistical Association 47, 663–685.

Stevens Jr, D.L., Olsen, A.R., 2003. Variance estimation for spatially balanced samples of environmental resources. Environmetrics 14, 593–610.

Stoddard, J.L., Larsen, D.P., Hawkins, C.P., Johnson, R.K., Norris, R.H., 2006. Setting expectations for the ecological condition of streams: The concept of reference condition. Ecological Applications 16, 1267–1276.

USEPA, 2023. National Wetland Condition Assessment: 2016 Technical Support Document. U.S. Environmental Protection Agency. Washington, DC, USA. URL: https://www.epa.gov/system/files/documents/2023-04/NWCA%202016%20Technical%20Support%20Document_20230216.pdf.

Appendix S5

Missing data in ecology: Syntheses, clarifications, and considerations

Michael Dumelle, Rob Trangucci, Amanda M. Nahlik, Anthony R. Olsen, Kathryn M. Irvine, Karen Blocksom, Jay M. Ver Hoef, Claudio Fuentes

Ecological Monographs

Section S1 Deriving Prediction Standard Errors in Linear Regression Models

The Nonspatial Linear Regression Model

The linear regression model with independent and identically distributed (i.i.d) errors can be expressed in matrix notation as

$$\mathbf{y} = \mathbf{X}\boldsymbol{\beta} + \boldsymbol{\epsilon},$$

where $\mathbf{y}$ is an $n \times 1$ response vector, $\mathbf{X}$ is an $n \times p$ matrix of explanatory variables, $\boldsymbol{\beta}$ is a $p \times 1$ vector of slope parameters (i.e., fixed effects) that control the average impact of each explanatory variable (i.e. column of $\mathbf{X}$) on $\mathbf{y}$, and $\boldsymbol{\epsilon}$ is a vector of i.i.d. random errors with mean zero and common variance σ^2. It can be shown that the best linear unbiased estimator of $\boldsymbol{\beta}$ is given by

$$\hat{\boldsymbol{\beta}} = (\mathbf{X}^T\mathbf{X})^{-1}\mathbf{X}^T\mathbf{y},$$

where $(\cdot)^T$ is the transpose operator and $(\cdot)^{-1}$ is the inverse operator. It can be shown that the variance of $\hat{\boldsymbol{\beta}}$ is $\sigma^2(\mathbf{X}^T\mathbf{X})^{-1}$ (Rencher and Schaalje, 2008).

Suppose that $\mathbf{x}_{new}$ is a $1 \times p$ vector of explanatory variable values for a new unit and the goal is to use $\mathbf{x}_{new}$ to make a corresponding prediction of y_{new}. The best linear unbiased predictor of y_{new}

is $\hat{y}_{new} \equiv \mathbf{x}_{new}\hat{\boldsymbol{\beta}}$ with corresponding prediction variance given by (Rencher and Schaalje, 2008)

$$\text{Var}(y_{new} - \hat{y}_{new}) = \sigma^2 + \sigma^2 \mathbf{x}_{new}(\mathbf{X}^T\mathbf{X})^{-1}\mathbf{x}_{new}^T.$$

When σ^2 is unknown, σ^2 above is replace by an estimate, $\hat{\sigma}^2$.

The Spatial Linear Regression Model

The spatial linear regression model can be expressed in matrix notation as

$$\mathbf{y} = \mathbf{X}\boldsymbol{\beta} + \boldsymbol{\tau} + \boldsymbol{\epsilon},$$

where $\mathbf{y}$ is an $n \times 1$ response vector, $\mathbf{X}$ is an $n \times p$ matrix of explanatory variables, $\boldsymbol{\beta}$ is a $p \times 1$ vector of slope parameters (i.e., fixed effects) that control the average impact of each explanatory variable (i.e. column of $\mathbf{X}$) on $\mathbf{y}$, $\boldsymbol{\tau}$ is a vector of spatial random errors with mean zero and covariance matrix $\sigma_\tau^2\mathbf{R}$ where $\mathbf{R}$ is a correlation matrix, and $\boldsymbol{\epsilon}$ is a vector of i.i.d. random errors with mean zero and covariance matrix $\sigma_\epsilon^2\mathbf{I}$ where $\mathbf{I}$ is an identity matrix. The covariance of $\mathbf{y}$, equivalent to the covariance of $\boldsymbol{\tau} + \boldsymbol{\epsilon}$, is expressed in matrix form as as $\boldsymbol{\Sigma} \equiv \sigma_\tau^2\mathbf{R} + \sigma_\epsilon^2\mathbf{I}$. It can be shown that the best linear unbiased estimator of $\boldsymbol{\beta}$ is given by

$$\hat{\boldsymbol{\beta}} = (\mathbf{X}^T\boldsymbol{\Sigma}^{-1}\mathbf{X})^{-1}\mathbf{X}^T\boldsymbol{\Sigma}^{-1}\mathbf{y},$$

where $(\cdot)^T$ is the transpose operator and $(\cdot)^{-1}$ is the inverse operator. It can be shown that the variance of $\hat{\boldsymbol{\beta}}$ is $(\mathbf{X}^T\boldsymbol{\Sigma}^{-1}\mathbf{X})^{-1}$ (Zimmerman and Ver Hoef, 2024).

Suppose that $\mathbf{x}_{new}$ is a $1 \times p$ vector of explanatory variable values for a new unit and the goal is to use $\mathbf{x}_{new}$ to make a corresponding prediction of y_{new}. The best linear unbiased predictor of y_{new} is

$$\hat{y}_{new} = \mathbf{x}_{new}\hat{\boldsymbol{\beta}} + c_{new}\boldsymbol{\Sigma}^{-1}(\mathbf{y} - \mathbf{X}\hat{\boldsymbol{\beta}}),$$

where $c_{new} = \text{Cov}(y_{new}, \mathbf{y})$. The corresponding prediction variance given by (Zimmerman and

Ver Hoef, 2024)

$$\text{Var}(\text{y}_{new} - \hat{\text{y}}_{new}) = \sigma^2 - \text{c}_{new}\mathbf{\Sigma}^{-1}\text{c}_{new}^T + \mathbf{H}(\mathbf{X}^T\mathbf{\Sigma}^{-1}\mathbf{X})^{-1}\mathbf{H}^T,$$

where $\sigma^2 \equiv \sigma_\tau^2 + \sigma_\epsilon^2$ and $\mathbf{H} \equiv \mathbf{x}_{new} - \text{c}_{new}\mathbf{\Sigma}^{-1}\mathbf{X}$ When the covariance parameters, $\boldsymbol{\theta}$, are unknown, they are replaced by estimates, $\hat{\boldsymbol{\theta}}$, and the resulting covariance matrix is evaluated at these estimates. For example, $\mathbf{\Sigma}$ is evaluated at $\hat{\boldsymbol{\theta}}$ and denoted $\hat{\mathbf{\Sigma}}$.

References

Rencher, A.C., Schaalje, G.B., 2008. Linear models in statistics. John Wiley & Sons.

Zimmerman, D.L., Ver Hoef, J.M., 2024. Spatial Linear Models for Environmental Data. CRC Press.
